# Claudin-7 Is Frequently Overexpressed in Ovarian Cancer and Promotes Invasion

**DOI:** 10.1371/journal.pone.0022119

**Published:** 2011-07-15

**Authors:** Neetu Dahiya, Kevin G. Becker, William H. Wood,, Yongqing Zhang, Patrice J. Morin

**Affiliations:** 1 Laboratory of Cellular and Molecular Biology, National Institute on Aging, Baltimore, Maryland, United States of America; 2 Research Resource Branch, National Institute on Aging, Baltimore, Maryland, United States of America; 3 Department of Pathology, Johns Hopkins Medical Institutions, Baltimore, Maryland, United States of America; Roswell Park Cancer Institute, United States of America

## Abstract

**Background:**

Claudins are tight junction proteins that are involved in tight junction formation and function. Previous studies have shown that claudin-7 is frequently upregulated in epithelial ovarian cancer (EOC) along with claudin-3 and claudin-4. Here, we investigate in detail the expression patterns of claudin-7, as well as its possible functions in EOC.

**Methodology/Principal Findings:**

A total of 95 ovarian tissue samples (7 normal ovarian tissues, 65 serous carcinomas, 11 clear cell carcinomas, 8 endometrioid carcinomas and 4 mucinous carcinomas) were studied for claudin-7 expression. In real-time RT-PCR analysis, the gene for claudin-7, *CLDN7*, was found to be upregulated in all the tumor tissue samples studied. Similarly, immunohistochemical analysis and western blotting showed that claudin-7 protein was significantly overexpressed in the vast majority of EOCs. Small interfering RNA-mediated knockdown of claudin-7 in ovarian cancer cells led to significant changes in gene expression as measured by microarrays and validated by RT-PCR and immunoblotting. Analyses of the genes differentially expressed revealed that the genes altered in response to claudin-7 knockdown were associated with pathways implicated in various molecular and cellular functions such as cell cycle, cellular growth and proliferation, cell death, development, and cell movement. Through functional experiments in vitro, we found that both migration and invasion were altered in cells where *CLDN7* had been knocked down or overexpressed. Interestingly, claudin-7 expression was associated with a net increase in invasion, but also with a decrease in migration.

**Conclusion/Significance:**

Our work shows that claudin-7 is significantly upregulated in EOC and that it may be functionally involved in ovarian carcinoma invasion. *CLDN7* may therefore represent potential marker for ovarian cancer detection and a target for therapy.

## Introduction

Ovarian cancer is the sixth leading cause of cancer deaths in women worldwide [1]. Ovarian cancer is typically diagnosed as advanced disease when therapeutic options are limited and the prognosis very poor. The past several years have witnessed a number of advances in our understanding of the molecular mechanisms involved in ovarian cancer progression [2],[3], although much of the details remain unknown. Among the genes whose expression is frequently altered in ovarian cancer are those encoding the claudin family of tight junction proteins [4]–[7]. Claudins form the backbone of TJs and are therefore essential in epithelial compartmentalization and in controlling solute transfer across cell monolayers [8]–[10].

In cancer, normal TJ function and structure are typically altered and the TJs themselves are frequently disassembled [11],[12]. Recent gene expression profiling studies have reported altered expression of a number of TJ proteins in multiple cancers, including several claudin proteins [4],[6]. For example, claudin-3 and claudin-4 are elevated in ovarian, prostate, uterine and breast cancers, while claudin-1 is reduced in breast and prostate cancers.

Claudin-7 has been found downregulated in several cancers including esophageal [13],[14], head and neck [15] and prostate cancer [16]. Loss of claudin-7 was correlated with dedifferentiation in several different cancers [16]–[19]. In breast cancer, claudin-7 expression was also found to be decreased and inversely correlated with tumor grade and metastatic disease [17],[18], although there is evidence that claudin-7 may become re-expressed in metastases [20],[21]. It has therefore been suggested that the loss of claudin-7 is associated with a loss of cell adhesion and increased metastasis [14],[17]. However, claudin-7 has also been shown to be elevated in several cancers. For example, claudin-7 is known to be elevated in ovarian cancer [22]–[27] as well as other malignancies [28],[29]. The reason for the different patterns of changes observed in different cancers is currently unknown, but may be related to the exact roles of claudin-7 in different malignancies, making elucidation of these roles an important goal.

In this report, we first evaluate claudin-7 expression levels in ovarian tissue samples and cell lines using western blotting, RT-PCR, and immunohistochemistry and find that claudin-7 is widely expressed in ovarian tumors. Using microarrays, we find that many genes are differentially expressed following *CLDN7* knockdown and analysis of these genes suggests several pathways through which *CLDN7* may function in ovarian cancer. We show that *CLDN7* knockdown leads to a decrease in phospho-Erk and an increase in Raf-1 levels, suggesting a possible pathway for claudin-7 signaling in ovarian cancer. In addition, we find that *CLDN7* expression is associated with increased invasion, but decreased migration in multiple cell lines providing a functional link to *CLDN7* expression in ovarian carcinoma.

## Results

### *CLDN7* transcript and protein levels are elevated in ovarian cancer tissues

**Figure 1:**
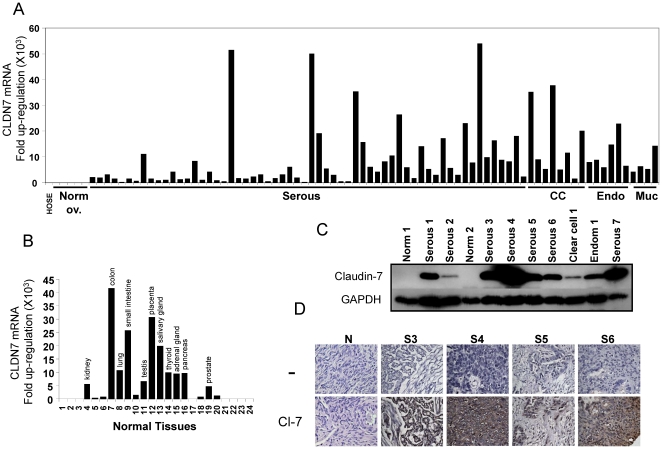
*CLDN7* expression in ovarian carcinoma. A. The indicated ovarian carcinoma subtypes were tested for *CLDN7* expression by RT-PCR and the levels are shown relative to HOSE-B cells (an ovarian surface epithelial cell line immortalized with E6 and E7 [52]). B. *CLDN7* expression in normal tissues. The following tissues were analyzed: 1, Muscle; 2, Brain; 3, Heart; 4, Kidney; 5, Spleen; 6, Liver; 7, Colon; 8, Lung; 9, Small Intestine; 10, Stomach; 11,Testis; 12, Placenta; 13, Salivary; 14, Thyroid; 15, Adrenal Gland; 16, Pancreas; 17,Uterus; 18, Ovary; 19, Prostate; 20, Skin; 21, Plasma Blood Leukocytes; 22, Bone Marrow; 23, Fetal Brain; 24, Fetal Liver. The levels are shown relative to muscle and tissues with levels higher than 4-fold are indicated on the graph. C. Immunoblot analysis of claudin-7 expression in 2 normal ovarian tissues (N1 and N2), 7 serous (S1, S2, S3, S4, S5, S6, and S7), one clear cell (C1) and one endometrioid (E1) ovarian carcinomas. A total of 29 samples were tested by immunoblotting and representative examples are shown. D. Immunohistochemistry of serous ovarian carcinomas. Selected ovarian carcinomas (S3, S4, S5, S6) and a normal ovarian sample (N) are shown. Ovarian cancer samples stained with claudin-7 antibody (Cl-7) exhibit strong staining compared to negative controls staining lacking primary antibody (-).

Recent work shows that, in addition to the *CLDN3* and *CLDN4* genes, *CLDN7* is frequently elevated in ovarian cancer [22]–[26], [30]. In order to clearly define the *CLDN7* expression patterns in ovarian cancer, microdissected ovarian tumors of various subtypes, as well as cell lines were analyzed by real-time RT-PCR. *CLDN7* mRNA was found to be highly up-regulated in all four major ovarian cancer subtypes (i.e. serous, mucinous, clear cell, and endometrioid) compared with normal ovarian tissues (Figure 1A). The level of overexpression varied tremendously and was found to be anywhere from 2-fold to 50,000-fold. Indeed, 2-fold increase over HOSE-B was observed in 2 of the normal ovary controls while 50,000-fold overexpression was seen in 3 serous ovarian cancer tissues. *CLDN7* is widely distributed in normal human tissues and its transcript was found at relatively high levels in kidney, colon, lung, small intestine, testis, placenta, salivary gland, thyroid, adrenal gland, pancreas, prostate, liver, stomach, ovary and skin (Figure 1B). On the other hand, muscle, brain, heart, spleen, uterus, blood leukocytes, bone marrow, fetal brain, and fetal liver expressed lower levels of *CLDN7* mRNA.

In order to investigate claudin-7 protein levels in ovarian cancer, a panel of 29 primary ovarian cancer samples as well as 4 normal ovarian tissues was studied by immunoblotting (Figure 1C, Table S1). Claudin-7 was absent in all the normal ovarian tissues, but was found at high/moderate levels in 66% (19/29) of the cancer samples. Claudin-7 was found expressed at low levels in 24% (7/29) of the cases, and was not detectable in 10% (3/29).

Immunohistochemistry (IHC) experiments confirmed that claudin-7 was expressed at high levels in ovarian cancer (Figure 1D). The IHC study demonstrated that claudin-7 overexpression was restricted to the cancer cells and did not involve the stroma. In addition, while certain tumors exhibited significant membrane staining (S3 and S4), others showed reduced staining at the membrane and high levels of cytoplasmic staining.

### *CLDN7* is elevated in ovarian cancer cell lines and can be located at the membrane or in the cytoplasm

**Figure 2:**
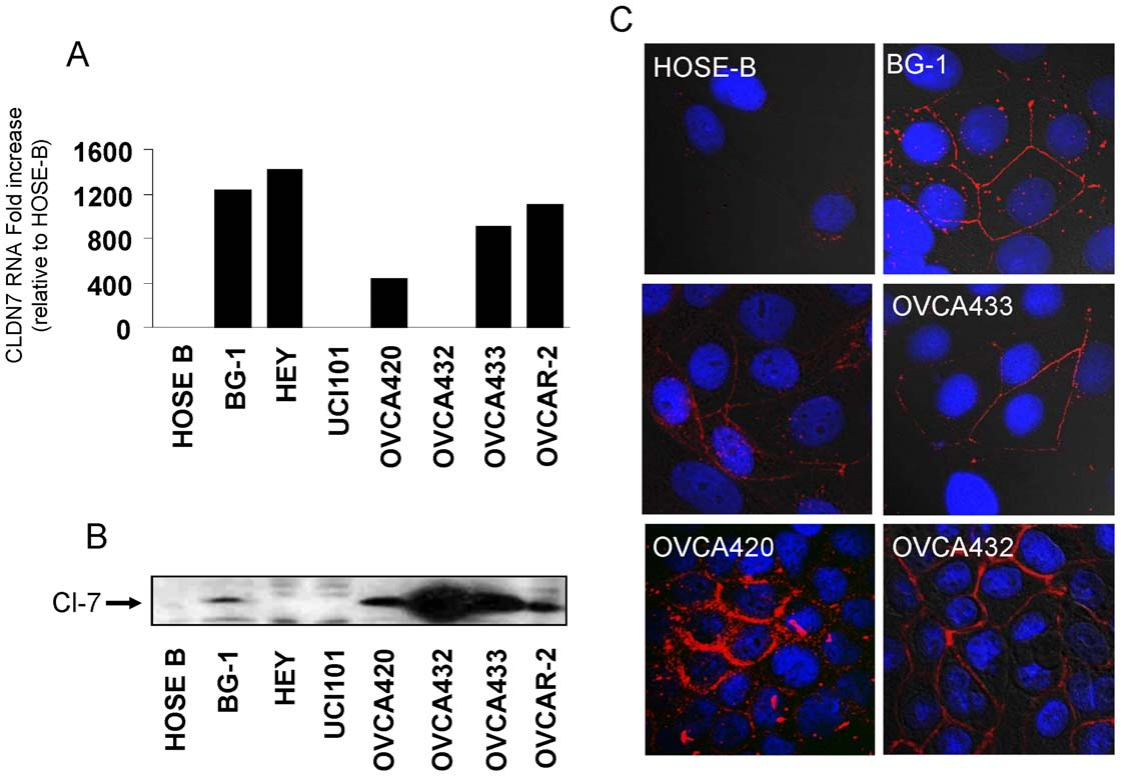
*CLDN7* expression in ovarian cancer cell lines. A. Claudin-7 immunoblotting reveals high levels of expression in the indicated ovarian cancer cell lines. B. RT-PCR analysis of *CLDN7* mRNA in cell lines. C. Immunofluorescence of selected cancer cell lines shows strong claudin-7 expression at the cell junction in BG-1, OVCA433, OVCA420 and OVCA432, as well as punctuate cytoplasmic staining in BG-1 and OVCA420. Claudin-7 was detected with an anti-claudin-7 antibody and visualized with secondary antibody conjugated to Alexa fluor. Nuclei of cells were counterstained with DAPI. Images were merged to determine correct localization.

In order to evaluate *CLDN7* expression in cell lines, 7 cell lines were evaluated by RT-PCR and immunoblotting (Figure 2A, B). The *CLDN7* transcript was not detected in the non-malignant surface epithelium line HOSE-B, but was found in all the ovarian cancer cell lines, except UCI101 and OVCA432 (Figure 2A). Similarly, claudin-7 protein was not detected in HOSE-B, but was present in most ovarian cancer cell lines (but not in HEY and UCI101) (Figure 2B). Interestingly, HEY cells showed high *CLDN7* mRNA levels, but low protein levels, while OVCA432 exhibited very low *CLDN7* transcript levels, but high claudin-7 protein expression. These data show that while claudin-7 transcription is retained in cell lines, claudin-7 protein levels may be regulated through post-transcriptional mechanisms.

Using immunofluorescence, claudin-7 staining was found localized predominantly at the cell membrane, with punctate staining in the cytoplasm of ovarian cancer cell lines BG-1, OVCA420 and OVCA433 (Figure 2C). In OVCA432, we also observed membrane staining, but little expression in the cytoplasm.

### Gene expression changes following *CLDN7* knockdown in ovarian cancer cells

**Figure 3:**
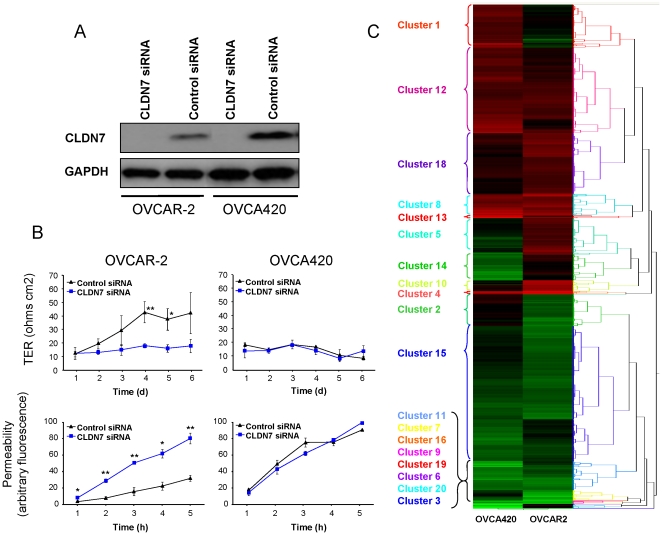
*CLDN7* knockdown and its effects on gene expression. A. Immunoblot analysis of OVCAR-2 and OVCA420 cells following *CLDN7* knockdown for 72 hrs. B. Effects of *CLDN7* knockdown on TJ and permeability. Transepithelial resistance (TER) was measured at multiple time-points following *CLDN7* knockdown. Permeability was measured by evaluating the ability of fluorescein-isothiocyanate-dextran (40 kDa) to cross the monolayer. Fluorescence measurements reflect monolayer permeability and the results mirror the TER measurements. Experiments were performed in triplicate and the results are shown as average +/− S.E.M. An asterisk (*) indicates a p-value<0.05, and a double asterisk (**), a p-value<0.01. C. Hierarchical clustering analysis of gene expression following *CLDN7* knockdown in both cell lines. The green color represents down-regulation, while red represents up-regulation. A total of 20 clusters of genes exhibiting distinct patterns were identified.

To investigate the possible roles of *CLDN7* in ovarian cancer, we examined gene expression changes following *CLDN7* knockdown. These knockdown experiments were performed in cell lines OVCAR-2 and OVCA420, both of which exhibit moderate endogenous levels of claudin-7 expression (Figure 3A). *CLDN7* siRNA treatment led to a significant reduction in claudin-7 protein 72 hours following transfection (Figure 3A) and this time point was chosen for microarray analysis. In OVCAR-2 cells, which form TJs in culture, *CLDN7* knockdown leads to a decrease in transepithelial resistance (TER, a measure of TJ tightness) and a concomitant increase in permeability across the monolayer (Figure 3B). This demonstrates that *CLDN7* knockdown has a functional effect on TJ in these cells. In OVCA420 cells, which do not form TJ in culture, TER was extremely low and remained unaffected by *CLDN7* knockdown (Figure 3B). Consistent with this finding, permeability was also unaffected by *CLDN7* knockdown.

**Figure 4:**
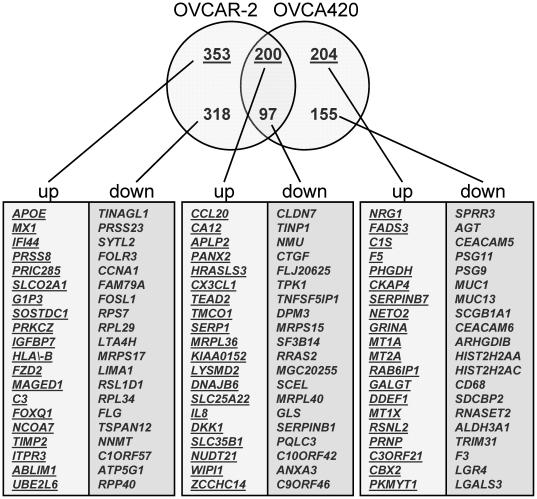
Venn diagram showing the genes up-regulated and down-regulated in OVCAR-2 and OVCA420 following *CLDN7* knockdown (absolute fold change>1.4). In the Venn diagram, numbers in red represent genes up-regulated and numbers in green represent genes downregulated. The top 20 genes in each category are indicated in the table below the Venn diagram.

Microarray analysis was used to investigate the effects of *CLDN7* knockdown on gene expression in both OVCAR-2 and OVCA420 cell lines. Clustering analysis demonstrated several patterns of changes following *CLDN7* knockdown and these patterns were represented by 20 different clusters of genes as identified by unsupervised pattern clustering using the JMP software (Figure 3C). *CLDN7* was the only gene in cluster 3 and, as expected, was highly downregulated in both cell lines. Of the 1327 genes differentially expressed in the two lines (Table S2), 297 (200 upregulated and 97 downregulated) were shared by OVCAR-2 and OVCA420 (Figure 4). This represents a 23% overlap of all the differentially expressed genes, a figure much higher than that expected by chance alone (p<0.001). In OVCAR-2, a total of 553 genes were upregulated and 415 were downregulated and in OVCA420, 404 genes were upregulated and 252 were downregulated. The top 20 genes up-regulated and down-regulated in each cell line (as well as in both simultaneously) are listed in Figure 4.

**Figure 5:**
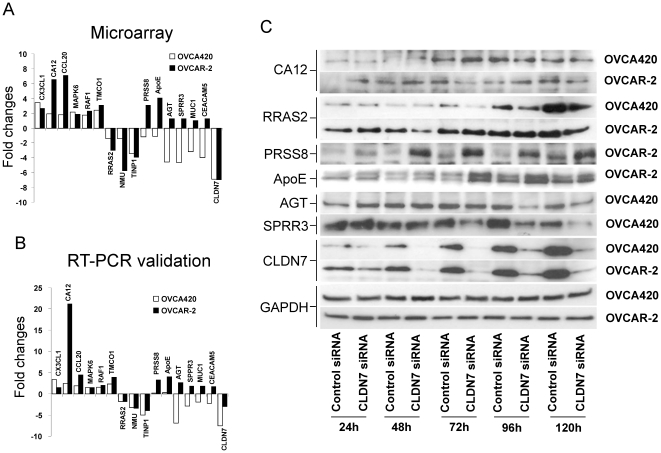
Validation of microarray data. A. Fold changes of the various indicated genes in OVCA420 and OVCAR-2 cells following *CLDN7* knockdown. B. RT-PCR validation of the genes shown in (A). C. Immunoblotting analysis of selected candidates at various time points following *CLDN7* knockdown. GAPDH control was included to confirm similar loading in all the lanes.

To validate and extend the microarray data, 15 genes that were significantly altered by *CLDN7* knockdown in one or both cell lines, were chosen for validation by RT–PCR analysis (Figure 5). While the fold expression appeared to be underestimated by microarray analysis, real-time PCR confirmed the trends for all the genes tested (Figure 5A, B). In addition, the protein levels for several of these genes (*CA12*, *RRAS2*, *PRSS8*, *APOE*, *AGT*, *SPRR3*, and *CLDN7*) were tested at different time points following *CLDN7* knockdown (Figure 5C). The results of these experiments were consistent with the changes observed by RT-PCR. Overall our data show an excellent correlation between the changes detected by microarrays and the corresponding protein expression levels.

### Pathway analysis

Using Ingenuity Pathway Analysis (IPA), we analyzed the pathways and possible functions associated with gene expression changes following *CLDN7* knockdown. Many of the top networks identified involved cancer, cellular growth and proliferation, but they were not clearly conserved between the cell lines (Table S3). Interestingly, “integrin signaling” was found in both cell lines among the most significant canonical pathways identified (Table S3). The top biological functions included a variety of functions such as cellular growth and cell death, and interestingly, cellular movement. The physiological systems identified included hematological and immune response, as well as connective tissue development (Table S3). Gene ontology analysis of altered genes identified significant categories such as “structural molecule activity”, “signal transducer activity”, “transcription regulator activity”, “translation regulator activity” and “transporter activity” (Table S4).

**Figure 6:**
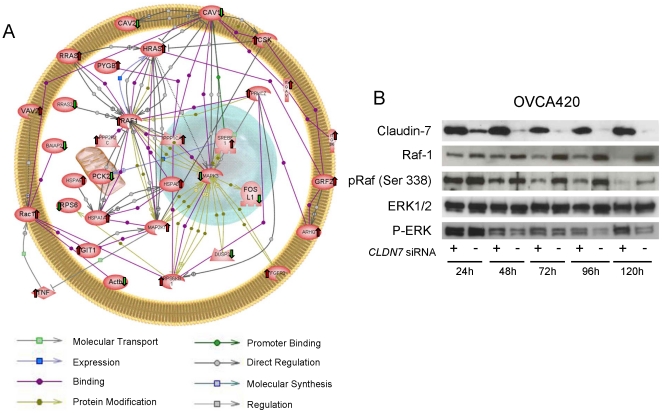
Pathways affected by *CLDN7* knockdown. A. Pathway Studio analysis. Direct interactions of 33 significantly upregulated (red upward arrow) or downregulated (green downward arrows) genes are shown. The legend for the different types of interactions is shown below the map. B. Immunoblot analysis of Erk1/2, phospho-Erk1/2 (p-Erk) and Raf-1 and phospho-Raf (Ser-338) in OVCA420 at the indicated time points following *CLDN7* knockdown.

The possible functional relationships between the various differentially expressed genes were then determined using a variety of approaches. The 5 most significant common KEGG pathways (as determined by Webgestalt) were “regulation of actin cytoskeleton”, “focal adhesion”, “MAPK signaling pathway”, and “insulin signaling pathway”. Table S5 list all the pathways identified. The top genes of the 5 most significant pathways listed above were used to search the Pathway Studio database for known protein-protein interactions (Figure 6A). Table S6 lists the protein-protein interaction details and their corresponding references. Interestingly one of the most prominent pathways identified through this approach was the RAS-RAF-MAPK pathway (Figure 6A). We then studied this pathway in OVCA420 cells by examining Erk and Raf-1 expression and phosphorylation in these cells (Figure 6B). Claudin-7 knockdown led to an increase in phosphorylated Raf-1 (Ser-338) that was mirrored by an increase in total Raf-1 at every time point, suggesting that indeed this pathway could be affected by claudin-7. Erk1/2 levels were not affected by claudin-7 knockdown, but phosphorylated Erk exhibited a slight decrease, especially at later time points.

### Claudin-7 protein expression is associated with cell invasion, but inversely correlated with migration

**Figure 7:**
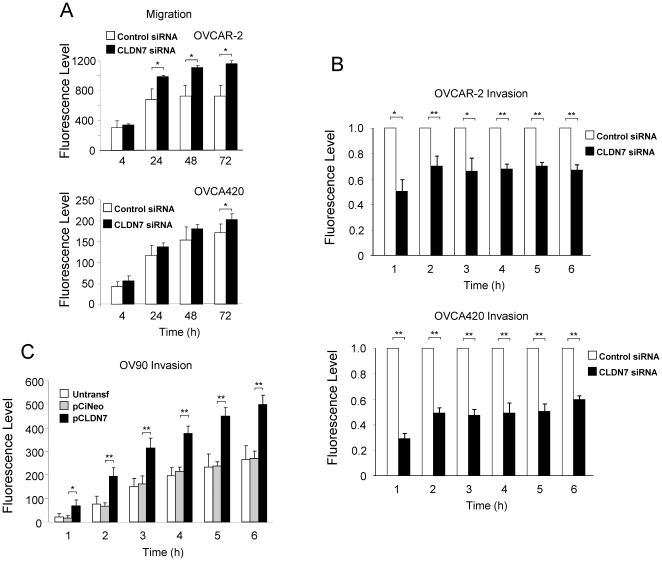
Effects of *CLDN7* on the migration and invasion of ovarian cancer cells. A. Migration assays in OVCAR-2 and OVCA420 cells. The migration of cells with *CLDN7* knocked down (dark bars) or control cells (white bars) was measured at different time point after the beginning of the assay. B. Boyden chamber invasion assay of OVCAR-2 and OVCA420 cells with and without *CLDN7* knockdown. The number of invading cells was measured every hour for 6 hours. C. Invasion assays in a model of *CLDN7* overexpression. Two stable lines, OV90-CLDN7 and OV90-CiNeo, as well as untransfected parental OV90 cells were used to test changes in invasion following *CLDN7* overexpression. For all the results shown in this figure, the experiments were performed in triplicate and the results are shown as mean +/− S.E.M. An asterisk (*) indicated a p-value<0.05, and a double asterisk (**), a p-value<0.01.

Because our gene analysis above reveals cell movement as a function possibly affected by *CLDN7*, we decided to test the possible roles of *CLDN7* in ovarian cancer cell migration and invasion. We first performed transient siRNA-mediated knockdown of *CLDN7* expression in two ovarian cancer cell lines (OVCAR-2 and OVCA420) and tested migration using the Oris cell migration assay. *CLDN7* knockdown led to a reproducible increase in cell migration in both cell lines at every time point tested (Figure 7A). Cell invasion was then evaluated using a Boyden chamber assay and, as shown in Figure 7B, inhibition of claudin-7 expression in OVCAR-2 and OVCA420 cells significantly reduced the invasive potential of these cells. To further confirm role of claudin-7 in invasion, claudin-7 was overexpressed in OV-90 cells (which lack endogenous claudin-7 expression) and found that stable overexpression of this protein led to increased invasion compared to mock-transfected cells or parental OV-90 cells (Figure 7C).

## Discussion

Our data show that *CLDN7* is elevated at both mRNA and protein levels in most ovarian cancer tissues and cell lines. By western analysis, primary ovarian tumor tissues exhibited variable expression of claudin-7 ranging from moderate to high expression in 66%, low in 24%, and undetectable in 10% tumor tissue samples. The reason for this variability is unclear, as the transcriptional regulation of *CLDN7* remains largely unknown. Interestingly, hypermethylation has be implicated in the downregulation of *CLDN7* expression in breast cancer [17] and two transcription factors, Tcf-4 and Sox9, have been shown to cooperate in *CLDN7* repression in colon cells [31]. It is unknown whether these mechanisms are involved in *CLDN7* regulation in ovarian cancer, but these possibilities are currently under investigation.

We find that *CLDN7* is elevated in all major subtypes of ovarian cancer: serous, endometrioid, clear cell and mucinous. These results are consistent with previous microarray data [22],[26] and IHC experiments [27],[30] showing that CLDN7 is typically overexpressed in epithelial ovarian cancer. Indeed, an early study showed that claudin-7 is overexpressed in all epithelial ovarian cancer subtypes, but is not overexpresssed in sex cord and stromal tumors [23]. Moreover, a detailed analysis by Tassi et al. [24] has shown that *CLDN7* is universally up-regulated in epithelial ovarian cancer at both the mRNA and protein levels. Our study also demonstrates overexpression in the most common epithelial ovarian cancer subtypes (serous, clear cell, endometrioid, and mucinous) at both the mRNA and protein levels using qRT-PCR, IHC, and immunoblotting. The use of immunoblotting and qRT-PCR allows us to demonstrate that mRNA levels and protein levels are not always correlated, suggesting post-translational regulation of claudin-7 in ovarian cancer.

Interestingly, while we found that most claudin-7 staining was localized to the membrane, several ovarian cancer samples exhibited strong cytoplasmic staining (Figure 1D). The fact that claudin proteins can be found in the cytoplasm has been reported and in particular, punctate cytoplasmic staining of claudin-7 has been observed in ovarian cancer [24]. The mechanisms and causes of claudin cytoplasmic localization remain unclear but several lines of evidence suggest that phosphorylation of claudins may affect their localization. For example, mutation of a PKA phosphorylation site in claudin-3 has been shown to lead to an increased cytoplasmic localization in ovarian cancer [32]. Similarly, claudin-4 was found to be phosphorylated by protein kinase C and this phosphorylation was found to lead to the translocation of claudin-4 from the membrane to the cytoplasm and a decrease in TJ function [32]. Claudin proteins are also known to be regulated by palmitoylation, as claudin-14 palmitoylation has been shown to be crucial for its proper localization [33]. It is currently unclear whether claudin-7 can be modified and whether modifications can affect its localization.

The overexpression of claudins in cancer suggests that claudins might have functional roles in tumorigenesis. Several groups have studied the possible roles of claudin-7 in cancer. Claudin-7 has been suggested to be involved in the regulation of prostate specific antigen (PSA) [34], possibly through interaction with junctional adhesion molecule A (JAM-A) [35]. Claudin-7 has also been reported to form a complex with EpCAM-tetraspanin which interferes with EpCAM-mediated cell adhesion properties and increases resistance to apoptosis [36]–[38]. Indeed, in colorectal carcinoma, EpCAM and claudin-7 are found in association with the tetraspanins CD9 and/or CO-029 and promote metastasis formation [37]. Additionally, EpCAM-*CLDN7* complex was suggested to have role in migration, proliferation, apoptosis resistance, metastasis, and tumorigenicity [37],[39].

In trying to gather additional clues regarding *CLDN7* function in ovarian cancer, we investigated gene expression changes following *CLDN7* knockdown. We hypothesized that downregulation of *CLDN7* may affect the downstream pathways affected by this protein and ultimately lead to expression changes in the genes regulated by these pathways. Between the two ovarian cancer cell lines studied, we identified a total of 1296 genes that were significantly altered following *CLDN7* knockdown. Among these genes, 297 were simultaneously found differentially expressed in both lines. Analysis of these genes using Ingenuity Pathway Analysis, led to the identification of several gene networks containing genes related to the functional categories “cancer”, “cell death”, “cell cycle”, “RNA trafficking”, “cellular development”, “cell signaling”, “cellular growth and proliferation”, and “cellular movement”. From the Kyoto Encyclopedia of Genes and Genomes (KEGG) pathway analysis, we selected the top 5 pathways that were altered in both OVCAR-2 and OVCA420 cell lines and generated a protein-protein interaction network map for these pathways. Using pathway Studio, we found direct interactions between 33 significantly upregulated or downregulated genes (Table S6). The interaction network showed a number of genes that have oncogenic or tumor suppressor roles. For example, RRAS2, which encode a Ras-related GTPase with transforming potential similar to H-, K- and N-Ras, is a well-known oncogene and has been implicated in the pathogenesis of human cancer. The pathways that mediate RRAS2-induced tumorigenesis are not well established, but phosphoinositide 3-kinase, p38 MAPK, and mTOR pathways have been implicated [40]. Of particular interest, RRAS2 and RRAS have been shown to promote invasion via integrin signaling in breast epithelial cells [41].

We have previously shown that ovarian epithelial cells that express claudin-3 and -4 show increased invasiveness *in vitro* [42], which was mediated through MMP-2 overexpression. Similarly, in hepatocellular carcinoma cells, claudin-10 expression promoted cell survival, motility, and invasiveness, while matrix metalloproteinase 2 (MMP2) was up-regulated [43]. However claudins have also been shown to reduce invasion as claudin-4 expression was associated with a reduction in invasiveness in pancreatic cancer cells [44]. Reduced expression of claudin-7 has been correlated with tumor invasion and metastasis in squamous cell carcinoma of the esophagus and in colorectal cancer [13],[45]. In this study we found that *CLDN7* knockdown led to changes in the expression of genes associated with various functions, including migration/invasion. In particular, following up on the pathway analysis results, we showed that Raf-1, a gene previously implicated in cell migration [46],[47], was up-regulated following *CLDN7* knockdown (Figure 6). Erk-1 was not found significantly elevated in these conditions, suggesting that Raf-1 may signal through other downstream pathways in response to *CLDN7* knockdown.

Based on our expression data, we then investigated the possible roles of *CLDN7* and found effects on invasion and migration *in vitro*. Indeed, invasion capacity was significantly reduced after *CLDN7* knockdown and, conversely, it was found increased after overexpression of *CLDN7*. Interestingly, migration was found to be increased following *CLDN7* knockdown (possibly because an increase in Raf-1), and these apparently contradictory findings (invasion vs migration) may explain previous observations in the field. Indeed, the exact roles of claudin on invasion have remained controversial, as some investigators observe positive effects of claudins on invasion and others have found the opposite. Since claudin proteins are known to activate MMPs [42], [48], but also have roles in cell-cell adhesion [9] and in migration (as shown here), the net effect observed may represent the outcome of these opposite effects on invasion. Our data suggest that while *CLDN7* overexpression can decrease migration, the increase in invasion due to other factors (such as increased MMP activation) is dominant and leads to a net positive effect on cell invasion in our system. The exact mechanisms of *CLDN7* roles in cell migration and invasion are under investigation, but our data may explain some of the discrepancies in the literature.

In summary, we find that *CLDN7* is elevated in the vast majority of ovarian cancers. We performed microarray analysis to identify changes in gene expression associated with *CLDN7* knockdown in ovarian cancer. Interestingly, we found that many genes and pathways affected by *CLDN7* expression are involved in cancer cell survival, growth, and invasion. Invasion and migration assays demonstrated clear effects of claudin-7 on the invasive behavior of ovarian cancer cells. Our microarray analysis provides several possible mechanisms by which claudin-7 can affect cell migration and invasion, and these different mechanisms are currently being investigated. In addition to their tight junction function, claudin-7 may play important roles in a number of signaling pathways involved in cancer, cellular growth, proliferation, and cell cycle. Given its important role in ovarian carcinogenesis, *CLDN7* may have significant potential in diagnostic and therapeutic applications.

## Materials and Methods

### Ethics Statement

All human tissues were collected through an IRB approved protocol (MedStar Research Institute IRB #2003-103). The tissues were collected anonymously and this protocol was exempted from the consent requirement.

### Cell Lines and Tissue Samples

Ovarian cancer lines used in this study, BG-1, UCI-101, HEY, OV90, OVCA420, OVCA432, OVCA433, OVCAR-2, OVCAR-3, and OVCAR-5 have been published [49]–[51]. All these lines were derived from serous epithelial ovarian cancers. These cell lines were cultured in McCoy's 5A growth medium (Invitrogen, Carlsbad, CA) supplemented with 10% fetal bovine serum (FBS) and antibiotics (100 units/ml penicillin and 100 mg/ml streptomycin). HOSE-B, an ovarian surface epithelial cell line immortalized with E6 and E7 [52], was cultured in RPMI1640 supplemented with 10% FBS, antibiotics and 5 ηg/ml EGF. Cell lines stably expressing *CLDN7* were generated by transfecting OV90 ovarian cancer cell line with pCIneo-*CLDN7* using Fugene-6, followed by selection in 500 µg/ml G418. Control stable lines were also generated by transfecting OV90 with the pCIneo vector. Two stable cell lines with control vector and three expressing CLDN7 cell lines were used for this study.

Forty-four primary ovarian cancer tissue samples as well as normal tissues were obtained through the Collaborative Human Tissue Network Gynecologic Oncology Group (Children's Hospital, Columbus, OH). In addition, twelve primary ovarian cancer samples (from primary ovarian cancer tissues) were obtained from the Department of Pathology at The Johns Hopkins Medical Institutions (Baltimore, MD). cDNA from 24 serous carcinomas, 6 clear cell carcinomas, 6 endometrioid carcinomas and 4 mucinous carcinomas were a generous gift from Dr. Kathleen R. Cho (University of Michigan). Histological classification and other available clinical information of the tissues used in this study can be found in Table S1. A total of 24 normal tissue cDNAs (covering the major organs) were purchased from BioChain (Hayward, CA) for RT-PCR analysis.

### Real-Time RT-PCR

RT-PCR was performed as previously described [53]. The SYBR Green I assay and the GeneAmp 5700 Sequence Detection System (Applied Biosystems, Carlsbad, California) were used for detecting real-time PCR products. The primers for *CLDN7* (forward, 5′-AGAGCACGGGGATGATGAG; reverse, 5′-CACCCATGGCTATACGGGC) and *GAPDH* (forward, 5′-GAAGGTGAAGGTCGGAGTC; reverse, 5′-GAAGATGGTGATGGGATTTC) were designed to cross intron-exon boundaries to distinguish PCR products generated from genomic *versus* cDNA template. The microarray data was validated by RT-PCR. The genes selected for validation represented a wide range of expression patterns (both in terms of direction and fold changes). The sequence of the primers used for RT-PCR validation of the microarray data is available from the authors. PCR reactions for each template were done in duplicate in 96-well plates. The comparative CT method (Applied Biosystems) was used to determine gene expression in each sample relative to the value observed in the nonmalignant HOSE-B, using *GAPDH* as normalization control.

### Antibodies

Rabbit polyclonal claudin-7 was purchased from Zymed Laboratories Inc (South San Francisco, CA). Mouse monoclonal CA12, RRAS2, SPRR3, GAPDH and Rabbit monoclonal RAF1 were obtained from Abcam (Cambridge, UK). Rabbit monoclonal anti-Ep-CAM (clone E144) antibodies were obtained from Novus Biologicals (Littleton, CO). Mouse polyclonal PRSS8 and AGT were obtained from Novus Biologicals and Mouse monoclonal ApoE was obtained from Genetex (Irvine, CA). Peroxidase-linked donkey anti-rabbit immunoglobulin and sheep anti-mouse IgG horseradish antibodies were obtained from Amersham Biosciences (GE Healthcare, Piscataway, NJ). Alexa fluor antibodies were purchased from Invitrogen.

### Immunoblotting, Immunohistochemistry, and Immunofluorescence

Immunoblotting was performed as previously described [42]. After SDS-PAGE and transfer, the PVDF membranes were blocked and then probed with anti-claudin-7 antibody (Zymed) diluted 1∶200. The blots were then washed and incubated in horseradish peroxidase-conjugated secondary antibody (anti-mouse or anti-rabbit IgG, 1∶10,000; Amersham Biosciences Corp.). For detection, enhanced chemiluminescence was carried out using the enhanced chemiluminescence kit (ECL; Amersham Biosciences Corp.). Immunohistochemistry was performed using the Ultravision detection system (Labvision, Fermont CA) according to the manufacturer's recommendations. Images were acquired by Axiovision 3.1 software on a Zeiss Axiovert S100 microscope under 40 objective lens (Carl Zeiss, Thornwood, NY). For immunofluorescence, HOSE-B, BG-1, OVCA420, OVCA432, OVCA433, and OVCAR-2 were grown to 90% confluency on glass slides and were washed with phosphate buffered saline (PBS) followed by fixation with cold methanol for 10 min at −20°C, fixed cells were washed, blocked and incubated with primary anti-claudin-7 antibodies in blocking buffer (mAb and pAb anti-claudin-7 at 1∶100) for 1 h. Slides were then washed and incubated with Alexa fluor-conjugated secondary antibodies. Cells were then incubated with DAPI for 2–3 min and then mounted in Prolong gold antifade mounting medium (Invitrogen). Fluorescent signal was examined and imaged using a LSM 510 Meta confocal microscope (Zeiss, Thornwood, NY).

### siRNA knockdown

Claudin-7 specific siRNA oligos were purchased from Ambion, Inc. (Austin, TX). Cells cultured in 6-well plates were transfected with siRNA duplexes using LipofectAMINE 2000 (Invitrogen) according to the manufacturer's instructions. Cells were treated for 72 hours to allow maximum knockdown, after which they were harvested for Western blot analysis, RNA preparation or used for other assays.

### TER Measurements and Permeability Assay

TER measurement and permeability assays were performed as previously described [42],[54]. Briefly, for TER measurements, cells were plated at a density of 1×10^5^ cells/well in 12-well Transwell filters and TER was measured using a Millicell-ERS epithelial V-ohmmeter (World Precision Instruments, New Haven, CT) as previously described [42]. For the permeability assays, the transport of fluorescein isothiocyanate-dextran (average mass 40 kDa, Sigma-Aldrich, St. Louis, MO) across a monolayer of confluent cells was monitored as previously described [54].

### Illumina Microarray

Total RNA, obtained using Trizol, was quantified and assessed using the RNA 6000 Nano Kit and 2100 Bioanalyzer (Agilent Technologies UK Ltd, West Lothian, UK). Biotinylated cRNA was prepared using the Illumina RNA Amplification Kit (Ambion, Inc., Austin, TX) according to the manufacturer's directions starting with approximately 500 ηg total RNA. Hybridization to the Sentrix HumanRef-8 Expression BeadChip (Illumina, Inc., San Diego, CA), washing and scanning were performed according to the Illumina BeadStation 5006 manual (revision C). Microarray data processing was done using Illumina Bead Studio software. Microarray analysis was performed essentially as described [55]. Raw microarray data were subjected to filtering and z-normalization. Sample quality was assessed using scatterplots and gene sample z-score-based hierarchical clustering. Expression changes for individual genes were considered significant if they met 4 criteria: z-ratio above 1.4 (or below −1.4 for down-regulated genes); false detection rate <0.30; p-value of the pairwise t-test<0.05; and mean background-corrected signal intensity z-score in each comparison group is not negative. This approach provides a good balance between sensitivity and specificity in the identification of differentially expressed genes, avoiding excessive representation of false positive and false negative regulation [56].

Hierarchical clustering analysis of the significant genes was done using the JMP 6.0.0 software. Pattern clustering was obtained by unsupervised k-mean clustering to identify gene pattern combinations. A total of 20 patterns were identified. All the microarray data are MIAME compliant and the raw data were deposited in GEO database (accession number GSE26055).

### Bioinformatics analysis

Genes differentially expressed were analyzed using Ingenuity Pathway Analysis (Redwood City, California) and the web-based webgestalt pathway analysis (http://bioinfo.vanderbilt.edu/webgestalt) [57]. The network genes associated with biological functions and/or diseases in the Ingenuity Pathways Knowledge Base were considered for the analysis. We used significant selected Human gene list as background gene list and multiple testing was controlled by both ANOVA and FDR criteria. Fischer's exact test was used to calculate a p-value determining the probability that each biological function and/or disease assigned to that network is due to chance alone.

### Invasion and migration assays

The cell invasion capabilities of siRNA-transfected cells were determined using a modified Boyden chamber invasion assay [58] essentially as described [42]. Experiments were repeated at least four times, with triplicate samples in each experiment. The migration assays were performed using the Oris Cell Migration Assay system (Platypus Technologies, Madison WI). Silicone cell stoppers were inserted in each well in a 96 well plate to prevent attachment of the cells in the center of the well (2 mm diameter). After 72 hours of transfection with control siRNA or *CLDN7* siRNA, cells were treated with 5 µmol/L calcein-AM (Molecular Probes, Eugene, OR) for 1 hour at 37°C. Cells were then trypsinized and 50,000 viable cells were plated in each well of a 96-well plate and incubated at 37°C. After overnight incubation, cell migration was measured by reading fluorescence in a Cytofluor 4000 at different time points, starting immediately after removing the stoppers (0 hour) for up to 72 hours. All assays were performed in triplicate. The data is shown as mean +/− S.E.M.

### Statistical Analysis

2-tailed t-test analysis was performed to determine significance. P values<0.05 were considered significant.

## Supporting Information

Table S1 **List of ovarian tissue samples used for the study.**
(PDF)

Table S2 **List of significantly altered genes in OVCAR2 and OVCA420 following** ***CLDN7*** **knockdown.**
(PDF)

Table S3 **Ingenuity Pathway Analysis results for genes altered following** ***CLDN7*** **knockdown.** A. Top networks. B. Top Canonical pathways. C. Top Biological functions.
(PDF)

Table S4 **GO analysis of significantly altered genes in OVCAR2 and OVCA420 following** ***CLDN7*** **knockdown.**
(PDF)

Table S5 **KEGG pathways enriched following** ***CLDN7*** **knockdown.**
(PDF)

Table S6 **Experimentally-validated interactions among the differentially regulated genes using Pathway Studio.**
(PDF)
