# Supplementary material for: Claudin-7 Is Frequently Overexpressed in Ovarian Cancer and Promotes Invasion
Source: PLoS One. 2011 Jul 15;6(7):e22119. doi: 10.1371/journal.pone.0022119 (PMC3137611; doi:10.1371/journal.pone.0022119)
Supplement: Table S1 — List of ovarian tissue samples used for the study. (PDF) [file pone.0022119.s001.pdf]

Supp. Table 1: List of ovarian tissue samples used for the study

Sample Name	General Classification	Age	Grade, Stage
HOSE B	Human Ovarian Surface Epithelium	N/A	N/A
N 1	Normal ovary	N/A	N/A
N 2	Normal ovary	N/A	N/A
N 3	Normal ovary	N/A	N/A
N 4	Normal ovary	N/A	N/A
N 5	Normal ovary	N/A	N/A
N 6	Normal ovary	N/A	N/A
N 7	Normal ovary	N/A	N/A
Tumor 1	Borderline carcinoma	57	unknown
Serous 2	Serous carcinoma low grade	55	Grade 2, stage 3
Serous 3	Serous carcinoma low grade	73	Grade 2, stage 3
Serous 4	Serous carcinoma	53	Stage 4
Serous 5	Serous carcinoma	57	unknown
Serous 6	Serous carcinoma		unknown
Serous 7	Serous carcinoma	69	unknown
Serous 8	Serous carcinoma low grade	76	Grade 3c
Serous 9	Serous carcinoma high grade	74	Grade 3
Serous 10	Serous carcinoma high grade	64	Grade 3
Serous 11	Serous carcinoma high grade	66	pT3c Nx Mx, grade 3 of 3
Serous 12	Serous carcinoma high grade	69	Grade 3c
Serous 13	Serous carcinoma high grade	48	Grade 3c
Serous 14	Serous carcinoma high grade	46	Grade 3
Serous 15	Serous carcinoma high grade	86	Grade 3
Serous 16	Serous carcinoma high grade	52	Grade 3c
Serous 17	Serous carcinoma high grade	64	unknown
Serous 18	Serous carcinoma high grade	61	Grade 3 , stage 3
Serous 19	Serous carcinoma high grade	66	ovarian CA stage 3
Serous 20	Serous carcinoma high grade	56	FIGO grade 3 of 3
Serous 21	Serous carcinoma high grade	76	Grade 3, stage 3
Serous 22	Serous carcinoma high grade	64	unknown
Serous 23	Serous carcinoma high grade	71	primary ovarian
Serous 24	Serous carcinoma high grade	63	Grade 3
Serous 25	Serous carcinoma high grade	77	unknown
Serous 26	Serous carcinoma high grade	68	AJCC stage IIIC

Serous 27	Serous carcinoma high grade	80	Grade 3
Serous 28	Serous carcinoma high grade	64	Grade 3, stage 4
Serous 29	Serous carcinoma high grade	57	Grade 3, stage 3
Serous 30	Serous carcinoma high grade	68	unknown
Serous 31	Serous carcinoma high grade	64	Grade 4
Serous 32	Serous carcinoma high grade	51	Grade 4
Serous 33	Serous carcinoma high grade	64	T3CNxM1
Serous 34	Serous carcinoma high grade	N/A	N/A
Serous 35	Serous carcinoma high grade	N/A	N/A
Serous 36	Serous carcinoma high grade	65	N/A
Serous 37	Serous carcinoma high grade	64	N/A
Serous 38	Serous carcinoma high grade	66	N/A
Serous 39	Serous carcinoma high grade	68	N/A
Serous 40	Serous carcinoma high grade	79	N/A
Serous 41	Serous carcinoma high grade	53	N/A
Serous 42	Serous carcinoma high grade	N/A	N/A
Serous 43	Serous carcinoma high grade	N/A	N/A
Serous 44	Serous cystadenocarcinoma	44	N/A
Serous 45	Serous carcinoma	N/A	cDNA panel
Serous 46	Serous carcinoma	N/A	N/A
Serous 47	Serous carcinoma	N/A	N/A
Serous 48	Serous carcinoma	N/A	N/A
Serous 49	Serous carcinoma	N/A	N/A
Serous 50	Serous carcinoma	N/A	N/A
Serous 51	Serous carcinoma	N/A	N/A
Serous 52	Serous carcinoma	N/A	N/A
Serous 53	Serous carcinoma	N/A	N/A
Serous 54	Serous carcinoma	N/A	N/A
Serous 55	Serous carcinoma	N/A	N/A
Serous 56	Serous carcinoma	N/A	N/A
Serous 57	Serous carcinoma	N/A	N/A
Serous 58	Serous carcinoma	N/A	N/A
Serous 59	Serous carcinoma	N/A	N/A
Serous 60	Serous carcinoma	N/A	N/A
Serous 61	Serous carcinoma	N/A	N/A
Serous 62	Serous carcinoma	N/A	N/A
Serous 63	Serous carcinoma	N/A	N/A

Serous 64	Serous carcinoma	N/A	N/A
Serous 65	Serous carcinoma	N/A	N/A
Serous 66	Serous carcinoma	N/A	N/A
Clear 1	Clear cell carcinoma	N/A	N/A
Clear 2	Clear cell carcinoma	N/A	N/A
Clear 3	Clear cell carcinoma	N/A	N/A
Clear 4	Clear cell carcinoma	N/A	N/A
Clear 5	Clear cell carcinoma	N/A	N/A
Clear 6	Clear cell carcinoma	N/A	N/A
Clear 7	Clear cell carcinoma	N/A	N/A
Clear 8	Clear cell carcinoma	N/A	N/A
Clear 9	Clear cell carcinoma	50	unknown
Clear 10	Clear cell carcinoma	72	2 to grade 3
Clear 11	Clear cell carcinoma	70	N/A
Endo 1	Endometrium adenocarcinoma high [illegible]	77	N/A
Endo 2	Endometrium adenocarcinoma high [illegible]	41	N/A
Endo 3	Endometrium carcinoma	N/A	N/A
Endo 4	Endometrium carcinoma	N/A	N/A
Endo 5	Endometrium carcinoma	N/A	N/A
Endo 6	Endometrium carcinoma	N/A	N/A
Endo 7	Endometrium carcinoma	N/A	N/A
Endo 8	Endometrium carcinoma	N/A	N/A
Muc 1	Mucinous carcinoma	N/A	N/A
Muc 2	Mucinous carcinoma	N/A	N/A
Muc 3	Mucinous carcinoma	N/A	N/A
Muc 4	Mucinous carcinoma	N/A	N/A
