# Supplementary material for: Claudin-7 Is Frequently Overexpressed in Ovarian Cancer and Promotes Invasion
Source: PLoS One. 2011 Jul 15;6(7):e22119. doi: 10.1371/journal.pone.0022119 (PMC3137611; doi:10.1371/journal.pone.0022119)
Supplement: Table S2 — List of significantly altered genes in OVCAR2 and OVCA420 following CLDN7 knockdown. (PDF) [file pone.0022119.s002.pdf]

Supp Table 2: List of significantly altered genes in OVCAR2 and OVCA420

Significant gene list in OVCAR2

Input ID	Fold	Gene ID	Symbol	Name
CCL20	7.06	6364	CCL20	chemokine (C-C motif) ligand 20
CA12	6.52	771	CA12	carbonic anhydrase XII
APLP2	5.01	334	APLP2	amyloid beta (A4) precursor-like protein 2
APOE	4.02	348	APOE	apolipoprotein E
HRASLS3	3.93	11145	HRASLS3	HRAS-like suppressor 3
PANX2	3.89	56666	PANX2	pannexin 2
TEAD2	3.73	8463	TEAD2	TEA domain family member 2
MX1	3.28	4599	MX1	myxovirus resistance 1, interferon-inducible protein p78
IFI44	3.25	10561	IFI44	interferon-induced protein 44
CREB3L2	3.19	64764	CREB3L2	cAMP responsive element binding protein 3-like 2
SERP1	3.19	27230	SERP1	stress-associated endoplasmic reticulum protein 1
KIAA0152	3.16	9761	KIAA0152	KIAA0152
TMCO1	3.10	54499	TMCO1	transmembrane and coiled-coil domains 1
PRSS8	3.07	5652	PRSS8	protease, serine, 8 (prostasin)
WIPI1	3.02	55062	WIPI1	WD repeat domain, phosphoinositide interacting 1
PRIC285	2.87	85441	PRIC285	peroxisomal proliferator-activated receptor A interacting complex 285
SLCO2A1	2.85	6578	SLCO2A1	solute carrier organic anion transporter family, member 2A1
G1P3	2.82	2537	G1P3	interferon, alpha-inducible protein 6
SOSTDC1	2.75	25928	SOSTDC1	sclerostin domain containing 1
SLC35B1	2.73	10237	SLC35B1	solute carrier family 35, member B1
PRKCZ	2.71	5590	PRKCZ	protein kinase C, zeta
IGFBP7	2.70	3490	IGFBP7	insulin-like growth factor binding protein 7
DAZAP2	2.69	9802	DAZAP2	DAZ associated protein 2
BID	2.68	637	BID	BH3 interacting domain death agonist
HLA B	2.68	3106	HLA B	major histocompatibility complex, class I, B
CX3CL1	2.65	6376	CX3CL1	chemokine (C-X3-C motif) ligand 1
FZD2	2.64	2535	FZD2	frizzled homolog 2 (Drosophila)
DNAJB6	2.63	10049	DNAJB6	DnaJ (Hsp40) homolog, subfamily B, member 6
LYSMD2	2.61	256586	LYSMD2	LysM, putative peptidoglycan-binding, domain containing 2
MAGED1	2.57	9500	MAGED1	melanoma antigen family D, 1
C3	2.55	718	C3	complement component 3
FOXQ1	2.55	94234	FOXQ1	forkhead box Q1
NCOA7	2.55	135112	NCOA7	nuclear receptor coactivator 7
ZNF544	2.55	27300	ZNF544	zinc finger protein 544
TIMP2	2.54	7077	TIMP2	TIMP metallopeptidase inhibitor 2
GPT2	2.50	84706	GPT2	glutamic pyruvate transaminase (alanine aminotransferase) 2
ITPR3	2.48	3710	ITPR3	inositol 1,4,5-triphosphate receptor, type 3
SET	2.47	6418	SET	SET translocation (myeloid leukemia-associated)
ABLIM1	2.46	3983	ABLIM1	actin binding LIM protein 1
TTYH3	2.46	80727	TTYH3	tweety homolog 3 (Drosophila)
ZDHHC14	2.46	79683	ZDHHC14	zinc finger, DHHC-type containing 14
CYBRD1	2.45	79901	CYBRD1	cytochrome b reductase 1

UBE2L6	2.45	9246	UBE2L6	ubiquitin-conjugating enzyme E2L 6
ZCCHC14	2.45	23174	ZCCHC14	zinc finger, CCHC domain containing 14
SURF4	2.43	6836	SURF4	surfeit 4
NUDT21	2.42	11051	NUDT21	nudix (nucleoside diphosphate linked moiety X)-type motif 21
PDIA4	2.42	9601	PDIA4	protein disulfide isomerase family A, member 4
CDH3	2.39	1001	CDH3	cadherin 3, type 1, P-cadherin (placental)
FGFR3	2.39	2261	FGFR3	dwarfism)
PIK3R2	2.39	5296	PIK3R2	phosphoinositide-3-kinase, regulatory subunit 2 (p85 beta)
WDR40A	2.39	25853	WDR40A	WD repeat domain 40A
FADS1	2.37	3992	FADS1	fatty acid desaturase 1
CXCL1	2.36	2919	CXCL1	activity, alpha)
DDIT4	2.36	54541	DDIT4	DNA-damage-inducible transcript 4
IL8	2.36	3576	IL8	interleukin 8
RKHD1	2.36	399664	RKHD1	ring finger and KH domain containing 1
TRPC6	2.35	7225	TRPC6	transient receptor potential cation channel, subfamily C, member 6
IMPA2	2.33	3613	IMPA2	inositol(myo)-1(or 4)-monophosphatase 2
PCSK1N	2.33	27344	PCSK1N	proprotein convertase subtilisin/kexin type 1 inhibitor
C20ORF160	2.32	140706	C20ORF160	chromosome 20 open reading frame 160
PPIA	2.31	5478	PPIA	peptidylprolyl isomerase A (cyclophilin A)
C1	2.31	55808	ST6GALNAC1	acetylgalactosaminide alpha-2,6-sialyltransferase 1
CYFIP2	2.29	26999	CYFIP2	cytoplasmic FMR1 interacting protein 2
LRP10	2.28	26020	LRP10	low density lipoprotein receptor-related protein 10
HMGCS1	2.27	3157	HMGCS1	3-hydroxy-3-methylglutaryl-Coenzyme A synthase 1 (soluble)
CXADR	2.27	1525	CXADR	coxsackie virus and adenovirus receptor
DNM1L	2.27	10059	DNM1L	dynamin 1-like
RAF1	2.27	5894	RAF1	v-raf-1 murine leukemia viral oncogene homolog 1
FUCA1	2.26	2517	FUCA1	fucosidase, alpha-L- 1, tissue
LTB	2.26	4050	LTB	lymphotoxin beta (TNF superfamily, member 3)
HOXA5	2.25	3202	HOXA5	homeobox A5
TRIM8	2.25	81603	TRIM8	tripartite motif-containing 8
SCHIP1	2.22	29970	SCHIP1	schwannomin interacting protein 1
IFI16	2.21	3428	IFI16	interferon, gamma-inducible protein 16
FYCO1	2.20	79443	FYCO1	FYVE and coiled-coil domain containing 1
MRPL36	2.20	64979	MRPL36	mitochondrial ribosomal protein L36
TUBB2B	2.20	347733	TUBB2B	tubulin, beta 2B
C17ORF59	2.19	54785	C17ORF59	chromosome 17 open reading frame 59
IER3	2.19	8870	IER3	immediate early response 3
A01247	2.19	22998	LIMCH1	LIM and calponin homology domains-containing protein 1
CARD9	2.18	64170	CARD9	caspase recruitment domain family, member 9
MAP1LC3B	2.18	81631	MAP1LC3B	microtubule-associated protein 1 light chain 3 beta
MFGE8	2.17	4240	MFGE8	milk fat globule-EGF factor 8 protein
C5ORF13	2.16	9315	C5ORF13	chromosome 5 open reading frame 13
LOC152485	2.16	152485	ZNF827	zinc finger protein 827
DSCR1	2.15	1827	DSCR1	Down syndrome critical region gene 1
TSPAN13	2.15	27075	TSPAN13	tetraspanin 13
SCARB2	2.14	950	SCARB2	scavenger receptor class B, member 2

STARD10	2.14	10809	STARD10	START domain containing 10
ATP6AP1	2.12	537	ATP6AP1	ATPase, H+ transporting, lysosomal accessory protein 1
SLC29A4	2.12	222962	SLC29A4	solute carrier family 29 (nucleoside transporters), member 4
SLC37A1	2.11	54020	SLC37A1	solute carrier family 37 (glycerol-3-phosphate transporter), member 1
SLC39A6	2.10	25800	SLC39A6	solute carrier family 39 (zinc transporter), member 6
DKK1	2.09	22943	DKK1	dickkopf homolog 1 (Xenopus laevis)
HLA A	2.09	3105	HLA A	major histocompatibility complex, class I, A
ACSL4	2.08	2182	ACSL4	acyl-CoA synthetase long-chain family member 4
DYNLL2	2.08	140735	DYNLL2	dynein, light chain, LC8-type 2
FBXO21	2.07	23014	FBXO21	F-box protein 21
FSCN1	2.06	6624	FSCN1	purpuratus)
IER5L	2.06	389792	IER5L	immediate early response 5-like
COL18A1	2.04	80781	COL18A1	collagen, type XVIII, alpha 1
JARID1B	2.04	10765	JARID1B	jumonji, AT rich interactive domain 1B
ST3GAL5	2.04	8869	ST3GAL5	ST3 beta-galactoside alpha-2,3-sialyltransferase 5
TNFSF10	2.04	8743	TNFSF10	tumor necrosis factor (ligand) superfamily, member 10
COPB2	2.03	9276	COPB2	coatomer protein complex, subunit beta 2 (beta prime)
PAPSS2	2.02	9060	PAPSS2	3'-phosphoadenosine 5'-phosphosulfate synthase 2
CDK2AP1	2.02	8099	CDK2AP1	CDK2-associated protein 1
DHRS3	2.02	9249	DHRS3	dehydrogenase/reductase (SDR family) member 3
PAQR8	2.02	85315	PAQR8	progestin and adipoQ receptor family member VIII
TMEM125	2.02	128218	TMEM125	transmembrane protein 125
PIK4CA	2.01	5297	PIK4CA	phosphatidylinositol 4-kinase, catalytic, alpha polypeptide
IMPAD1	2.00	54928	IMPAD1	inositol monophosphatase domain containing 1
MAN2B2	2.00	23324	MAN2B2	mannosidase, alpha, class 2B, member 2
FAM38A	1.99	9780	FAM38A	family with sequence similarity 38, member A
MXD4	1.99	10608	MXD4	MAX dimerization protein 4
MLLT11	1.99	10962	MLLT11	Drosophila); translocated to, 11
RRAS	1.99	6237	RRAS	related RAS viral (r-ras) oncogene homolog
TJP3	1.99	27134	TJP3	tight junction protein 3 (zona occludens 3)
CDC25B	1.98	994	CDC25B	cell division cycle 25B
CREG1	1.98	8804	CREG1	cellular repressor of E1A-stimulated genes 1
EPSTI1	1.97	94240	EPSTI1	epithelial stromal interaction 1 (breast)
PGBD5	1.97	79605	PGBD5	piggyBac transposable element derived 5
RAB15	1.97	376267	RAB15	RAB15, member RAS onocogene family
VANGL2	1.97	57216	VANGL2	vang-like 2 (van gogh, Drosophila)
FUT4	1.96	2526	FUT4	fucosyltransferase 4 (alpha (1,3) fucosyltransferase, myeloid-specific)
LPIN1	1.96	23175	LPIN1	lipin 1
MAP2K1	1.96	5604	MAP2K1	mitogen-activated protein kinase kinase 1
CEBPB	1.95	1051	CEBPB	CCAAT/enhancer binding protein (C/EBP), beta
KIF1A	1.95	547	KIF1A	kinesin family member 1A
C6ORF85	1.95	63027	SLC22A23	solute carrier family 22, member 23
RNF149	1.94	284996	RNF149	ring finger protein 149
CHFR	1.93	55743	CHFR	checkpoint with forkhead and ring finger domains
MGC13057	1.93	84281	C2ORF88	chromosome 2 open reading frame 88
EIF2AK2	1.93	5610	EIF2AK2	eukaryotic translation initiation factor 2-alpha kinase 2

TRIM5	1.93	85363	TRIM5	tripartite motif-containing 5
NLF2	1.92	388125	C2CD4B	C2 calcium-dependent domain containing 4B
CEBPD	1.92	1052	CEBPD	CCAAT/enhancer binding protein (C/EBP), delta
IFITM3	1.92	10410	IFITM3	interferon induced transmembrane protein 3 (1-8U)
MMP1	1.92	4312	MMP1	matrix metallopeptidase 1 (interstitial collagenase)
LOC339344	1.92	339344	MYPOP	Myb-related transcription factor, partner of profilin
TFAP2A	1.92	7020	TFAP2A	alpha)
ABR	1.91	29	ABR	active BCR-related gene
ARID1A	1.91	8289	ARID1A	AT rich interactive domain 1A (SWI- like)
HSPA1A	1.91	3303	HSPA1A	heat shock 70kDa protein 1A
NR1H2	1.91	7376	NR1H2	nuclear receptor subfamily 1, group H, member 2
TM9SF4	1.91	9777	TM9SF4	transmembrane 9 superfamily protein member 4
CXCL6	1.90	6372	CXCL6	chemokine (C-X-C motif) ligand 6 (granulocyte chemotactic protein 2)
GNAI2	1.90	2771	GNAI2	polypeptide 2
PODXL2	1.90	50512	PODXL2	podocalyxin-like 2
ALKBH7	1.89	84266	ALKBH7	alkB, alkylation repair homolog 7 (E. coli)
PJA2	1.89	9867	PJA2	praja 2, RING-H2 motif containing
VPS39	1.89	23339	VPS39	vacuolar protein sorting 39 (yeast)
2123	1.89	83637	ZMIZ2	zinc finger, MIZ-type containing 2
FLJ22471	1.88	80212	CCDC92	coiled-coil domain containing 92
EXTL3	1.88	2137	EXTL3	exostoses (multiple)-like 3
GDF15	1.88	9518	GDF15	growth differentiation factor 15
IGF2R	1.88	3482	IGF2R	insulin-like growth factor 2 receptor
FLJ25476	1.88	149076	ZNF362	zinc finger protein 362
CLTC	1.87	1213	CLTC	clathrin, heavy chain (Hc)
MAPK6	1.87	5597	MAPK6	mitogen-activated protein kinase 6
RAPGEF1	1.87	2889	RAPGEF1	Rap guanine nucleotide exchange factor (GEF) 1
ABHD8	1.86	79575	ABHD8	abhydrolase domain containing 8
ARL6IP5	1.86	10550	ARL6IP5	ADP-ribosylation-like factor 6 interacting protein 5
CXCL2	1.86	2920	CXCL2	chemokine (C-X-C motif) ligand 2
CGN	1.86	57530	CGN	cingulin
HSPA1B	1.86	3304	HSPA1B	heat shock 70kDa protein 1B
ID1	1.86	3397	ID1	inhibitor of DNA binding 1, dominant negative helix-loop-helix protein
UBE2D3	1.86	7323	UBE2D3	ubiquitin-conjugating enzyme E2D 3 (UBC4/5 homolog, yeast)
PRKD2	1.85	25865	PRKD2	protein kinase D2
RUTBC1	1.85	9905	RUTBC1	RUN and TBC1 domain containing 1
SH3GL3	1.85	6457	SH3GL3	SH3-domain GRB2-like 3
SLC25A22	1.85	79751	SLC25A22	solute carrier family 25 (mitochondrial carrier: glutamate), member 22
FCGRT	1.84	2217	FCGRT	Fc fragment of IgG, receptor, transporter, alpha
FBLN1	1.84	2192	FBLN1	fibulin 1
ORF1	1.84	55354	ORF1	hypothetical protein, clone pT-Adv JuaX22
NFIL3	1.84	4783	NFIL3	nuclear factor, interleukin 3 regulated
TNFAIP2	1.84	7127	TNFAIP2	tumor necrosis factor, alpha-induced protein 2
LMBR1L	1.83	55716	LMBR1L	limb region 1 homolog (mouse)-like
SASH1	1.83	23328	SASH1	SAM and SH3 domain containing 1
UBE2F	1.83	140739	UBE2F	ubiquitin-conjugating enzyme E2F (putative)

ARPC1A	1.82	10552	ARPC1A	actin related protein 2/3 complex, subunit 1A, 41kDa
HOXA2	1.82	3199	HOXA2	homeobox A2
IGFBP3	1.82	3486	IGFBP3	insulin-like growth factor binding protein 3
IFI35	1.82	3430	IFI35	interferon-induced protein 35
MLLT6	1.82	4302	MLLT6	Drosophila); translocated to, 6
PLXNB1	1.82	5364	PLXNB1	plexin B1
PVRL2	1.82	5819	PVRL2	poliovirus receptor-related 2 (herpesvirus entry mediator B)
P4HB	1.82	5034	P4HB	hydroxylase), beta polypeptide
RRAGA	1.82	10670	RRAGA	Ras-related GTP binding A
PLEKHH3	1.81	79990	PLEKHH3	domain) member 3
SMARCA4	1.81	6597	SMARCA4	chromatin, subfamily a, member 4
ARFGAP3	1.80	26286	ARFGAP3	ADP-ribosylation factor GTPase activating protein 3
C17ORF63	1.80	55731	C17ORF63	chromosome 17 open reading frame 63
GFPT1	1.80	2673	GFPT1	glutamine-fructose-6-phosphate transaminase 1
IFITM2	1.80	10581	IFITM2	interferon induced transmembrane protein 2 (1-8D)
HLA C	1.80	3107	HLA C	major histocompatibility complex, class I, C
MET	1.80	4233	MET	met proto-oncogene (hepatocyte growth factor receptor)
MYH10	1.80	4628	MYH10	myosin, heavy chain 10, non-muscle
PPFIBP2	1.80	8495	PPFIBP2	PTPRF interacting protein, binding protein 2 (liprin beta 2)
ANAPC1	1.79	64682	ANAPC1	anaphase promoting complex subunit 1
CEACAM1	1.79	634	CEACAM1	glycoprotein)
DDR1	1.79	780	DDR1	discoidin domain receptor family, member 1
NMD3	1.79	51068	NMD3	NMD3 homolog (S. cerevisiae)
RPS6KB1	1.79	6198	RPS6KB1	ribosomal protein S6 kinase, 70kDa, polypeptide 1
FLJ31951	1.79	153830	RNF145	ring finger protein 145
ADAR	1.78	103	ADAR	adenosine deaminase, RNA-specific
CAB39	1.78	51719	CAB39	calcium binding protein 39
GBA	1.78	2629	GBA	glucosidase, beta; acid (includes glucosylceramidase)
HERC5	1.78	51191	HERC5	hect domain and RLD 5
RDH10	1.78	157506	RDH10	retinol dehydrogenase 10 (all-trans)
PTPNS1	1.78	140885	SIRPA	signal-regulatory protein alpha
SFXN3	1.77	81855	SFXN3	sideroflexin 3
TMC6	1.77	11322	TMC6	transmembrane channel-like 6
IFITM1	1.76	8519	IFITM1	interferon induced transmembrane protein 1 (9-27)
LPHN1	1.76	22859	LPHN1	latrophilin 1
ROD1	1.76	9991	ROD1	ROD1 regulator of differentiation 1 (S. pombe)
SEPN1	1.76	57190	SEPN1	selenoprotein N, 1
TMEM47	1.76	83604	TMEM47	transmembrane protein 47
C20ORF22	1.75	26090	ABHD12	abhydrolase domain containing 12
BTG3	1.75	10950	BTG3	BTG family, member 3
MGC24665	1.75	116028	C16orf75	chromosome 16 open reading frame 75
CYB5R3	1.75	1727	CYB5R3	cytochrome b5 reductase 3
IL4I1	1.75	259307	IL4I1	interleukin 4 induced 1
IQWD1	1.75	55827	IQWD1	IQ motif and WD repeats 1
NAGLU	1.75	4669	NAGLU	N-acetylglucosaminidase, alpha- (Sanfilippo disease IIIB)
PARP10	1.75	84875	PARP10	poly (ADP-ribose) polymerase family, member 10

RAN	1.75	5901	RAN	RAN, member RAS oncogene family
2-Sep	1.75	4735	2-Sep	septin 2
SNX27	1.75	81609	SNX27	sorting nexin family member 27
TNF	1.75	7124	TNF	tumor necrosis factor (TNF superfamily, member 2)
CPVL	1.74	54504	CPVL	carboxypeptidase, vitellogenic-like
CDS2	1.74	8760	CDS2	CDP-diacylglycerol synthase (phosphatidate cytidylyltransferase) 2
CCNG1	1.74	900	CCNG1	cyclin G1
DBN1	1.74	1627	DBN1	drebrin 1
FLOT2	1.74	2319	FLOT2	flotillin 2
KIAA0195	1.74	9772	KIAA0195	KIAA0195
LAMB2	1.74	3913	LAMB2	laminin, beta 2 (laminin S)
LEPREL2	1.74	10536	LEPREL2	leprecan-like 2
SEC61A1	1.74	29927	SEC61A1	Sec61 alpha 1 subunit (S. cerevisiae)
SRP46	1.74	10929	SFRS2B	splicing factor, arginine/serine-rich 2B
TEGT	1.74	7009	TEGT	testis enhanced gene transcript (BAX inhibitor 1)
ZNF358	1.74	140467	ZNF358	zinc finger protein 358
ADD3	1.73	120	ADD3	adducin 3 (gamma)
MGC12981	1.73	84317	CCDC115	coiled-coil domain containing 115
FAM32A	1.73	26017	FAM32A	family with sequence similarity 32, member A
HPRT1	1.73	3251	HPRT1	hypoxanthine phosphoribosyltransferase 1 (Lesch-Nyhan syndrome)
NOP5	1.73	51602	NOP58	NOP58 ribonucleoprotein homolog (yeast)
PIGQ	1.73	9091	PIGQ	phosphatidylinositol glycan anchor biosynthesis, class Q
PIN1	1.73	5300	PIN1	protein (peptidylprolyl cis/trans isomerase) NIMA-interacting 1
LNK	1.73	10019	SH2B3	SH2B adaptor protein 3
TMEM59	1.73	9528	TMEM59	transmembrane protein 59
ZNF447	1.73	65982	ZNF447	zinc finger protein 447
ATG9A	1.72	79065	ATG9A	ATG9 autophagy related 9 homolog A (S. cerevisiae)
MGC16385	1.72	92806	CENPBD1	CENPB DNA-binding domains containing 1
PCGF2	1.72	7703	PCGF2	polycomb group ring finger 2
SFRP1	1.72	6422	SFRP1	secreted frizzled-related protein 1
9-Sep	1.72	10801	9-Sep	septin 9
ZNF419	1.72	79744	ZNF419	zinc finger protein 419
COL4A5	1.71	1287	COL4A5	collagen, type IV, alpha 5 (Alport syndrome)
CSK	1.71	1445	CSK	c-src tyrosine kinase
EVI5L	1.71	115704	EVI5L	ecotropic viral integration site 5-like
FAM50A	1.71	9130	FAM50A	family with sequence similarity 50, member A
FOXC1	1.71	2296	FOXC1	forkhead box C1
POR	1.71	5447	POR	P450 (cytochrome) oxidoreductase
WFS1	1.71	7466	WFS1	Wolfram syndrome 1 (wolframin)
DNM2	1.70	1785	DNM2	dynamin 2
H2AFY2	1.70	55506	H2AFY2	H2A histone family, member Y2
LLGL1	1.70	3996	LLGL1	lethal giant larvae homolog 1 (Drosophila)
NME4	1.70	4833	NME4	non-metastatic cells 4, protein expressed in
POLR3H	1.70	171568	POLR3H	polymerase (RNA) III (DNA directed) polypeptide H (22.9kD)
SEPHS2	1.70	22928	SEPHS2	selenophosphate synthetase 2
SUPT4H1	1.70	6827	SUPT4H1	suppressor of Ty 4 homolog 1 (S. cerevisiae)

TGIF2	1.70	60436	TGIF2	TGFB-induced factor 2 (TALE family homeobox)
FLJ38101	1.70	255919	TMEM188	transmembrane protein 188
UBN1	1.70	29855	UBN1	ubinuclein 1
CD81	1.69	975	CD81	CD81 molecule
HSU79303	1.69	29903	CCDC106	coiled-coil domain containing 106
CUEDC1	1.69	404093	CUEDC1	CUE domain containing 1
PHF21A	1.69	51317	PHF21A	PHD finger protein 21A
CENTD3	1.68	64411	CENTD3	centaurin, delta 3
CENPB	1.68	1059	CENPB	centromere protein B, 80kDa
FLJ11017	1.68	55286	C4orf19	chromosome 4 open reading frame 19
ID2	1.68	3398	ID2	inhibitor of DNA binding 2, dominant negative helix-loop-helix protein
ZNF219	1.68	51222	ZNF219	zinc finger protein 219
IFNGR1	1.67	3459	IFNGR1	interferon gamma receptor 1
MLF2	1.67	8079	MLF2	myeloid leukemia factor 2
PACS1	1.67	55690	PACS1	phosphofurin acidic cluster sorting protein 1
PGM1	1.67	5236	PGM1	phosphoglucomutase 1
RBP1	1.67	5947	RBP1	retinol binding protein 1, cellular
SC65	1.67	10609	SC65	synaptonemal complex protein SC65
GALNAC4S	1.66	51363	CHST15	15
CCBP2	1.66	1238	CCBP2	chemokine binding protein 2
GCC1	1.66	79571	GCC1	GRIP and coiled-coil domain containing 1
LOC613266	1.66	613266	LOC613266	hypothetical LOC613266
IL4R	1.66	3566	IL4R	interleukin 4 receptor
LIMK1	1.66	3984	LIMK1	LIM domain kinase 1
PLXNA1	1.66	5361	PLXNA1	plexin A1
ST6GAL1	1.66	6480	ST6GAL1	ST6 beta-galactosamide alpha-2,6-sialyltranferase 1
TMCO3	1.66	55002	TMCO3	transmembrane and coiled-coil domains 3
TMEM41A	1.66	90407	TMEM41A	transmembrane protein 41A
ZNF614	1.66	80110	ZNF614	zinc finger protein 614
BCKDK	1.65	10295	BCKDK	branched chain ketoacid dehydrogenase kinase
CHST3	1.65	9469	CHST3	carbohydrate (chondroitin 6) sulfotransferase 3
OSBPL2	1.65	9885	OSBPL2	oxysterol binding protein-like 2
PHF23	1.65	79142	PHF23	PHD finger protein 23
TNFRSF14	1.65	8764	TNFRSF14	entry mediator)
B3GNT6	1.65	192134	B3GNT6	(core 3 synthase)
ZNF161	1.65	7716	VEZF1	vascular endothelial zinc finger 1
FLJ20920	1.64	80221	ACSF2	acyl-CoA synthetase family member 2
ARFGEF1	1.64	10565	ARFGEF1	1(brefeldin A-inhibited)
ANXA4	1.64	307	ANXA4	annexin A4
LRAP	1.64	64167	ERAP2	endoplasmic reticulum aminopeptidase 2
FOXJ3	1.64	22887	FOXJ3	forkhead box J3
LIPA	1.64	3988	LIPA	lipase A, lysosomal acid, cholesterol esterase (Wolman disease)
MSRB2	1.64	22921	MSRB2	methionine sulfoxide reductase B2
S100A9	1.64	6280	S100A9	S100 calcium binding protein A9
STC2	1.64	8614	STC2	stanniocalcin 2
SREBF1	1.64	6720	SREBF1	sterol regulatory element binding transcription factor 1

TSPAN17	1.64	26262	TSPAN17	tetraspanin 17
TOMM34	1.64	10953	TOMM34	translocase of outer mitochondrial membrane 34
UCRC	1.64	29796	UQCR10	ubiquinol-cytochrome c reductase, complex III subunit X
UBTD1	1.64	80019	UBTD1	ubiquitin domain containing 1
ZC3H12A	1.64	80149	ZC3H12A	zinc finger CCCH-type containing 12A
AGPAT5	1.63	55326	AGPAT5	acyltransferase, epsilon)
ATP1B3	1.63	483	ATP1B3	ATPase, Na+/K+ transporting, beta 3 polypeptide
BRD3	1.63	8019	BRD3	bromodomain containing 3
CDC2L6	1.63	23097	CDC2L6	cell division cycle 2-like 6 (CDK8-like)
CTDSP2	1.63	10106	CTDSP2	small phosphatase 2
ITPK1	1.63	3705	ITPK1	inositol 1,3,4-triphosphate 5/6 kinase
PPP1CA	1.63	5499	PPP1CA	protein phosphatase 1, catalytic subunit, alpha isoform
SLC11A2	1.63	4891	SLC11A2	transporters), member 2
ZDHHC16	1.63	84287	ZDHHC16	zinc finger, DHHC-type containing 16
AP2M1	1.62	1173	AP2M1	adaptor-related protein complex 2, mu 1 subunit
AKR1C3	1.62	8644	AKR1C3	dehydrogenase, type II)
CCND2	1.62	894	CCND2	cyclin D2
KLHL5	1.62	51088	KLHL5	kelch-like 5 (Drosophila)
LYCAT	1.62	253558	LYCAT	lysocardiolipin acyltransferase
NINJ1	1.62	4814	NINJ1	ninjurin 1
ARHGEF5	1.62	7984	ARHGEF5	Rho guanine nucleotide exchange factor (GEF) 5
RHOBTB3	1.62	22836	RHOBTB3	Rho-related BTB domain containing 3
SORT1	1.62	6272	SORT1	sortilin 1
TBC1D14	1.62	57533	TBC1D14	TBC1 domain family, member 14
ACO2	1.61	50	ACO2	aconitase 2, mitochondrial
GDPD5	1.61	81544	GDPD5	glycerophosphodiester phosphodiesterase domain containing 5
GOLPH3L	1.61	55204	GOLPH3L	golgi phosphoprotein 3-like
HYOU1	1.61	10525	HYOU1	hypoxia up-regulated 1
ITM2C	1.61	81618	ITM2C	integral membrane protein 2C
KLHDC2	1.61	23588	KLHDC2	kelch domain containing 2
KIAA0350	1.61	23274	KIAA0350	KIAA0350
NRBP1	1.61	29959	NRBP1	nuclear receptor binding protein 1
PHGDHL1	1.61	337867	PHGDHL1	phosphoglycerate dehydrogenase like 1
PAFAH1B1	1.61	5048	PAFAH1B1	45kDa
P4HA1	1.61	5033	P4HA1	hydroxylase), alpha polypeptide I
PIAS3	1.61	10401	PIAS3	protein inhibitor of activated STAT, 3
RDH11	1.61	51109	RDH11	retinol dehydrogenase 11 (all-trans/9-cis/11-cis)
TNS3	1.61	64759	TNS3	tensin 3
TSPAN7	1.61	7102	TSPAN7	tetraspanin 7
UBE2Q2	1.61	92912	UBE2Q2	ubiquitin-conjugating enzyme E2Q (putative) 2
RAI17	1.61	57178	ZMIZ1	zinc finger, MIZ-type containing 1
ACSL5	1.60	51703	ACSL5	acyl-CoA synthetase long-chain family member 5
AP3D1	1.60	8943	AP3D1	adaptor-related protein complex 3, delta 1 subunit
FAM104A	1.60	84923	FAM104A	family with sequence similarity 104, member A
GRN	1.60	2896	GRN	granulin
HSPA5	1.60	3309	HSPA5	heat shock 70kDa protein 5 (glucose-regulated protein, 78kDa)

HSP90B1	1.60	7184	HSP90B1	heat shock protein 90kDa beta (Grp94), member 1
SFXN1	1.60	94081	SFXN1	sideroflexin 1
STAT5B	1.60	6777	STAT5B	signal transducer and activator of transcription 5B
SYF2	1.60	25949	SYF2	SYF2 homolog, RNA splicing factor (S. cerevisiae)
TSPAN3	1.60	10099	TSPAN3	tetraspanin 3
TSPAN6	1.60	7105	TSPAN6	tetraspanin 6
ZFP36L1	1.60	677	ZFP36L1	zinc finger protein 36, C3H type-like 1
CD74	1.59	972	CD74	chain
ELL	1.59	8178	ELL	elongation factor RNA polymerase II
HOXB4	1.59	3214	HOXB4	homeobox B4
ILF3	1.59	3609	ILF3	interleukin enhancer binding factor 3, 90kDa
TPP1	1.59	1200	TPP1	tripeptidyl peptidase I
CLDN15	1.58	24146	CLDN15	claudin 15
COQ10B	1.58	80219	COQ10B	coenzyme Q10 homolog B (S. cerevisiae)
DNAJB2	1.58	3300	DNAJB2	DnaJ (Hsp40) homolog, subfamily B, member 2
MTA3	1.58	57504	MTA3	metastasis associated 1 family, member 3
MUC20	1.58	200958	MUC20	mucin 20, cell surface associated
LOC133619	1.58	133619	PRRC1	proline-rich coiled-coil 1
SEMA4D	1.58	10507	SEMA4D	(TM) and short cytoplasmic domain, (semaphorin) 4D
SIPA1	1.58	6494	SIPA1	signal-induced proliferation-associated gene 1
TMSL3	1.58	7117	TMSL3	thymosin-like 3
TSC22D3	1.58	1831	TSC22D3	TSC22 domain family, member 3
ZZEF1	1.58	23140	ZZEF1	zinc finger, ZZ-type with EF-hand domain 1
BRPF3	1.57	27154	BRPF3	bromodomain and PHD finger containing, 3
CARD11	1.57	84433	CARD11	caspase recruitment domain family, member 11
CTSD	1.57	1509	CTSD	cathepsin D (lysosomal aspartyl peptidase)
CLASP1	1.57	23332	CLASP1	cytoplasmic linker associated protein 1
GM2A	1.57	2760	GM2A	GM2 ganglioside activator
PGRMC1	1.57	10857	PGRMC1	progesterone receptor membrane component 1
YTHDF3	1.57	253943	YTHDF3	YTH domain family, member 3
LBR	1.56	3930	LBR	lamin B receptor
LAPTM4B	1.56	55353	LAPTM4B	lysosomal associated protein transmembrane 4 beta
MAN1B1	1.56	11253	MAN1B1	mannosidase, alpha, class 1B, member 1
MGAT1	1.56	4245	MGAT1	acetylglucosaminyltransferase
MBTPS1	1.56	8720	MBTPS1	membrane-bound transcription factor peptidase, site 1
NR2F6	1.56	2063	NR2F6	nuclear receptor subfamily 2, group F, member 6
PLOD1	1.56	5351	PLOD1	procollagen-lysine 1, 2-oxoglutarate 5-dioxygenase 1
RNASEH1	1.56	246243	RNASEH1	ribonuclease H1
SIDT2	1.56	51092	SIDT2	SID1 transmembrane family, member 2
SPHK2	1.56	56848	SPHK2	sphingosine kinase 2
SPRY1	1.56	10252	SPRY1	sprouty homolog 1, antagonist of FGF signaling (Drosophila)
TST	1.56	7263	TST	thiosulfate sulfurtransferase (rhodanese)
TMEM113	1.56	80335	TMEM113	transmembrane protein 113
TSC22D4	1.56	81628	TSC22D4	TSC22 domain family, member 4
DHCR7	1.55	1717	DHCR7	7-dehydrocholesterol reductase
E2F6	1.55	1876	E2F6	E2F transcription factor 6

NDRG4	1.55	65009	NDRG4	NDRG family member 4
PTPLA	1.55	9200	PTPLA	member A
RHOQ	1.55	23433	RHOQ	ras homolog gene family, member Q
SPEN	1.55	23013	SPEN	spen homolog, transcriptional regulator (Drosophila)
DEDD2	1.54	162989	DEDD2	death effector domain containing 2
HN1	1.54	51155	HN1	hematological and neurological expressed 1
MAPRE3	1.54	22924	MAPRE3	microtubule-associated protein, RP/EB family, member 3
NCK2	1.54	8440	NCK2	NCK adaptor protein 2
STMN3	1.54	50861	STMN3	stathmin-like 3
TAF10	1.54	6881	TAF10	associated factor, 30kDa
TBL1XR1	1.54	79718	TBL1XR1	transducin (beta)-like 1X-linked receptor 1
TMEM32	1.54	93380	TMEM32	transmembrane protein 32
ANKRD33	1.53	341405	ANKRD33	ankyrin repeat domain 33
LOC388272	1.53	388272	C16orf87	chromosome 16 open reading frame 87
IRAK2	1.53	3656	IRAK2	interleukin-1 receptor-associated kinase 2
FLJ14154	1.53	79903	NAT15	N-acetyltransferase 15 (GCN5-related, putative)
NTE	1.53	10908	PNPLA6	patatin-like phospholipase domain containing 6
SNURF	1.53	8926	SNURF	SNRPN upstream reading frame
TYSND1	1.53	219743	TYSND1	trypsin domain containing 1
SLITL2	1.53	114990	VASN	vasorin
VAV2	1.53	7410	VAV2	vav 2 oncogene
DEGS1	1.52	8560	DEGS1	degenerative spermatocyte homolog 1, lipid desaturase (Drosophila)
DGCR2	1.52	9993	DGCR2	DiGeorge syndrome critical region gene 2
EVL	1.52	51466	EVL	Enah/Vasp-like
EEF1A2	1.52	1917	EEF1A2	eukaryotic translation elongation factor 1 alpha 2
ITM2B	1.52	9445	ITM2B	integral membrane protein 2B
LGALS3BP	1.52	3959	LGALS3BP	lectin, galactoside-binding, soluble, 3 binding protein
MYLIP	1.52	29116	MYLIP	myosin regulatory light chain interacting protein
PRKCD	1.52	5580	PRKCD	protein kinase C, delta
RHOU	1.52	58480	RHOU	ras homolog gene family, member U
SLC17A5	1.52	26503	SLC17A5	solute carrier family 17 (anion/sugar transporter), member 5
TMEM106C	1.52	79022	TMEM106C	transmembrane protein 106C
C7ORF28A	1.51	51622	C7orf28A	chromosome 7 open reading frame 28A
DAP	1.51	1611	DAP	death-associated protein
DPP4	1.51	1803	DPP4	protein 2)
FUT3	1.51	2525	FUT3	blood group)
IRF1	1.51	3659	IRF1	interferon regulatory factor 1
C3ORF9	1.51	56983	KTELC1	KTEL (Lys-Tyr-Glu-Leu) containing 1
KIAA1688	1.51	80728	ARHGAP39	Rho GTPase activating protein 39
TXNDC5	1.51	81567	TXNDC5	thioredoxin domain containing 5
ATAD4	1.50	79170	ATAD4	ATPase family, AAA domain containing 4
CPD	1.50	1362	CPD	carboxypeptidase D
C7ORF28B	1.50	51622	C7orf28A	chromosome 7 open reading frame 28A
KIAA0427	1.50	9811	KIAA0427	KIAA0427
LARP5	1.50	23185	LARP5	La ribonucleoprotein domain family, member 5
NIPA2	1.50	81614	NIPA2	non imprinted in Prader-Willi/Angelman syndrome 2

SCAP	1.50	22937	SCAP	SREBF chaperone
UBXD1	1.50	80700	UBXD1	UBX domain containing 1
MMD	1.49	23531	MMD	monocyte to macrophage differentiation-associated
NEK6	1.49	10783	NEK6	NIMA (never in mitosis gene a)-related kinase 6
PSAT1	1.49	29968	PSAT1	phosphoserine aminotransferase 1
PROM1	1.49	8842	PROM1	prominin 1
PPP1R16A	1.49	84988	PPP1R16A	protein phosphatase 1, regulatory (inhibitor) subunit 16A
RALGDS	1.49	5900	RALGDS	ral guanine nucleotide dissociation stimulator
SUMO3	1.49	6612	SUMO3	SMT3 suppressor of mif two 3 homolog 3 (S. cerevisiae)
SC4MOL	1.49	6307	SC4MOL	sterol-C4-methyl oxidase-like
ST14	1.49	6768	ST14	suppression of tumorigenicity 14 (colon carcinoma)
B2M	1.48	567	B2M	beta-2-microglobulin
CD63	1.48	967	CD63	CD63 molecule
CLTB	1.48	1212	CLTB	clathrin, light chain (Lcb)
EML4	1.48	27436	EML4	echinoderm microtubule associated protein like 4
HK1	1.48	3098	HK1	hexokinase 1
NOMO1	1.48	23420	NOMO1	NODAL modulator 1
RAD54L2	1.48	23132	RAD54L2	RAD54-like 2 (S. cerevisiae)
RCN2	1.48	5955	RCN2	reticulocalbin 2, EF-hand calcium binding domain
SLC16A3	1.48	9123	SLC16A3	solute carrier family 16, member 3 (monocarboxylic acid transporter 4)
XYLT2	1.48	64132	XYLT2	xylosyltransferase II
FLJ13149	1.48	60493	FASTKD5	FAST kinase domains 5
FLJ20186	1.48	54849	DEF8	Differentially expressed in FDCP 8 homolog (mouse)
LOC221955	1.48	-	-	-
CD47	1.47	961	CD47	CD47 molecule
CCND3	1.47	896	CCND3	cyclin D3
NPLOC4	1.47	55666	NPLOC4	nuclear protein localization 4 homolog (S. cerevisiae)
PHF12	1.47	57649	PHF12	PHD finger protein 12
PITPNM1	1.47	9600	PITPNM1	phosphatidylinositol transfer protein, membrane-associated 1
RABAC1	1.47	10567	RABAC1	Rab acceptor 1 (prenylated)
RPN2	1.47	6185	RPN2	ribophorin II
SSR1	1.47	6745	SSR1	signal sequence receptor, alpha
TLCD1	1.47	116238	TLCD1	TLC domain containing 1
USP39	1.47	10713	USP39	ubiquitin specific peptidase 39
WDR45L	1.47	56270	WDR45L	WDR45-like
11-Sep	1.47	55752	11-Sep	septin 11
ISGF3G	1.47	10379	IFR9	interferon-stimulated transcription factor 3, gamma
GAK	1.46	2580	GAK	cyclin G associated kinase
GULP1	1.46	51454	GULP1	GULP, engulfment adaptor PTB domain containing 1
MALL	1.46	7851	MALL	mal, T-cell differentiation protein-like
M6PR	1.46	4074	M6PR	mannose-6-phosphate receptor (cation dependent)
MAPK3	1.46	5595	MAPK3	mitogen-activated protein kinase 3
PDIA6	1.46	10130	PDIA6	protein disulfide isomerase family A, member 6
ZNF609	1.46	23060	ZNF609	zinc finger protein 609
FAM62A	1.46	23344	ESYT1	extended synaptotagmin-like protein 1
ALDH3A2	1.45	224	ALDH3A2	aldehyde dehydrogenase 3 family, member A2

CTTN	1.45	2017	CTTN	cortactin
CDK6	1.45	1021	CDK6	cyclin-dependent kinase 6
GTF3A	1.45	2971	GTF3A	general transcription factor IIIA
LACTB	1.45	114294	LACTB	lactamase, beta
NFIB	1.45	4781	NFIB	nuclear factor I/B
SCRN1	1.45	9805	SCRN1	secernin 1
STK35	1.45	140901	STK35	serine/threonine kinase 35
SERPINA3	1.45	12	SERPINA3	member 3
TXNIP	1.45	10628	TXNIP	thioredoxin interacting protein
TMED3	1.45	23423	TMED3	transmembrane emp24 protein transport domain containing 3
UCHL1	1.45	7345	UCHL1	ubiquitin carboxyl-terminal esterase L1 (ubiquitin thiolesterase)
CAPN5	1.44	726	CAPN5	calpain 5
CHURC1	1.44	91612	CHURC1	churchill domain containing 1
CORO1B	1.44	57175	CORO1B	coronin, actin binding protein, 1B
DBI	1.44	1622	DBI	Coenzyme A binding protein)
FTL	1.44	2512	FTL	ferritin, light polypeptide
HEBP2	1.44	23593	HEBP2	heme binding protein 2
JARID2	1.44	3720	JARID2	jumonji, AT rich interactive domain 2
KLHDC5	1.44	57542	KLHDC5	kelch domain containing 5
LARP6	1.44	55323	LARP6	La ribonucleoprotein domain family, member 6
MIF	1.44	4282	MIF	factor)
PFKM	1.44	5213	PFKM	phosphofructokinase, muscle
S100A14	1.44	57402	S100A14	S100 calcium binding protein A14
SHMT2	1.44	6472	SHMT2	serine hydroxymethyltransferase 2 (mitochondrial)
TMED9	1.44	54732	TMED9	transmembrane emp24 protein transport domain containing 9
LAMB1	1.43	3912	LAMB1	laminin, beta 1
MAP3K4	1.43	4216	MAP3K4	mitogen-activated protein kinase kinase kinase 4
PPL	1.43	5493	PPL	periplakin
PPP2R2C	1.43	5522	PPP2R2C	isoform
SUOX	1.43	6821	SUOX	sulfite oxidase
TNFRSF11B	1.43	4982	TNFRSF11B	tumor necrosis factor receptor superfamily, member 11b
ABHD4	1.42	63874	ABHD4	abhydrolase domain containing 4
CLNS1A	1.42	1207	CLNS1A	chloride channel, nucleotide-sensitive, 1A
CFB	1.42	629	CFB	complement factor B
GLT25D1	1.42	79709	GLT25D1	glycosyltransferase 25 domain containing 1
GNB1	1.42	2782	GNB1	guanine nucleotide binding protein (G protein), beta polypeptide 1
LCP1	1.42	3936	LCP1	lymphocyte cytosolic protein 1 (L-plastin)
NFKBIA	1.42	4792	NFKBIA	inhibitor, alpha
YWHAB	1.42	7529	YWHAB	protein, beta polypeptide
LOC203547	1.42	203547	VMA21	VMA21 vacuolar H+-ATPase homolog (S. cerevisiae)
PBEF1	1.42	10135	NAMPT	nicotinamide phosphoribosyltransferase
CAPN1	1.41	823	CAPN1	calpain 1, (mu/I) large subunit
KDELR2	1.41	11014	KDELR2	receptor 2
LCN2	1.41	3934	LCN2	lipocalin 2
PIGM	1.41	93183	PIGM	phosphatidylinositol glycan anchor biosynthesis, class M
ARHGAP27	1.41	201176	ARHGAP27	Rho GTPase activating protein 27

DNMT1	1.40	1786	DNMT1	DNA (cytosine-5-)-methyltransferase 1
SCARB1	1.40	949	SCARB1	scavenger receptor class B, member 1
SPIRE1	1.40	56907	SPIRE1	spire homolog 1 (Drosophila)
SPON1	1.40	10418	SPON1	spondin 1, extracellular matrix protein
TMEM9	1.40	252839	TMEM9	transmembrane protein 9
CCDC59	-1.40	29080	CCDC59	coiled-coil domain containing 59
CNIH	-1.40	10175	CNIH	cornichon homolog (Drosophila)
MRPS5	-1.40	64969	MRPS5	mitochondrial ribosomal protein S5
NSMCE1	-1.40	197370	NSMCE1	non-SMC element 1 homolog (S. cerevisiae)
RPS27L	-1.40	51065	RPS27L	ribosomal protein S27-like
TMPRSS4	-1.40	56649	TMPRSS4	transmembrane protease, serine 4
C2ORF4	-1.40	51072	MEMO1	mediator of cell motility 1
FLJ90586	-1.40	135932	TMEM139	transmembrane protein 139
LOC348262	-1.40	348262	FAM195B	family with sequence similarity 195, member B
CDK5RAP1	-1.41	51654	CDK5RAP1	CDK5 regulatory subunit associated protein 1
CHCHD3	-1.41	54927	CHCHD3	coiled-coil-helix-coiled-coil-helix domain containing 3
COX7A2	-1.41	1347	COX7A2	cytochrome c oxidase subunit VIIa polypeptide 2 (liver)
DHRS1	-1.41	115817	DHRS1	dehydrogenase/reductase (SDR family) member 1
FOLR1	-1.41	2348	FOLR1	folate receptor 1 (adult)
LYAR	-1.41	55646	LYAR	Ly1 antibody reactive homolog (mouse)
PAK1IP1	-1.41	55003	PAK1IP1	PAK1 interacting protein 1
RPS20	-1.41	6224	RPS20	ribosomal protein S20
RFWD2	-1.41	64326	RFWD2	ring finger and WD repeat domain 2
C14ORF32	-1.41	93487	MAPK1IP1L	mitogen-activated protein kinase 1 interacting protein 1-like
HSA9761	-1.41	27292	DIMT1L	DIM1 dimethyladenosine transferase 1-like (S. cerevisiae)
WDR68	-1.41	10238	DCAF7	DDB1 and CUL4 associated factor 7
CYP4F11	-1.42	57834	CYP4F11	cytochrome P450, family 4, subfamily F, polypeptide 11
DDX10	-1.42	1662	DDX10	DEAD (Asp-Glu-Ala-Asp) box polypeptide 10
DTYMK	-1.42	1841	DTYMK	deoxythymidylate kinase (thymidylate kinase)
GNL3L	-1.42	54552	GNL3L	guanine nucleotide binding protein-like 3 (nucleolar)-like
MXRA7	-1.42	439921	MXRA7	matrix-remodelling associated 7
MRPS23	-1.42	51649	MRPS23	mitochondrial ribosomal protein S23
RPL23A	-1.42	6147	RPL23A	ribosomal protein L23a
WBSCR22	-1.42	114049	WBSCR22	Williams Beuren syndrome chromosome region 22
WIBG	-1.42	84305	WIBG	within bgcn homolog (Drosophila)
C12ORF62	-1.42	84987	C12ORF62	chromosome 12 open reading frame 62
DNCL2A	-1.42	83658	DYNLRB1	dynein, light chain, roadblock-type 1
MAGMAS	-1.42	51025	PAM16	presequence translocase-associated motor 16 homolog (S. cerevisiae)
BCS1L	-1.43	617	BCS1L	BCS1-like (yeast)
CHMP4B	-1.43	128866	CHMP4B	chromatin modifying protein 4B
OSBPL10	-1.43	114884	OSBPL10	oxysterol binding protein-like 10
SNRPA1	-1.43	6627	SNRPA1	small nuclear ribonucleoprotein polypeptide A'
SNAPC2	-1.43	6618	SNAPC2	small nuclear RNA activating complex, polypeptide 2, 45kDa
SMARCC1	-1.43	6599	SMARCC1	chromatin, subfamily c, member 1
TRIP13	-1.43	9319	TRIP13	thyroid hormone receptor interactor 13
TMEM85	-1.43	51234	TMEM85	transmembrane protein 85

CSTF3	-1.44	1479	CSTF3	cleavage stimulation factor, 3' pre-RNA, subunit 3, 77kDa
COBLL1	-1.44	22837	COBLL1	COBL-like 1
CKB	-1.44	1152	CKB	creatine kinase, brain
CYR61	-1.44	3491	CYR61	cysteine-rich, angiogenic inducer, 61
DDT	-1.44	1652	DDT	D-dopachrome tautomerase
DYRK4	-1.44	8798	DYRK4	dual-specificity tyrosine-(Y)-phosphorylation regulated kinase 4
FN3KRP	-1.44	79672	FN3KRP	fructosamine 3 kinase related protein
GPS2	-1.44	2874	GPS2	G protein pathway suppressor 2
GPR110	-1.44	266977	GPR110	G protein-coupled receptor 110
GSTK1	-1.44	373156	GSTK1	glutathione S-transferase kappa 1
HMBS	-1.44	3145	HMBS	hydroxymethylbilane synthase
IFRD2	-1.44	7866	IFRD2	interferon-related developmental regulator 2
LTV1	-1.44	84946	LTV1	LTV1 homolog (S. cerevisiae)
NHP2L1	-1.44	4809	NHP2L1	NHP2 non-histone chromosome protein 2-like 1 (S. cerevisiae)
PROSC	-1.44	11212	PROSC	proline synthetase co-transcribed homolog (bacterial)
PSMA1	-1.44	5682	PSMA1	proteasome (prosome, macropain) subunit, alpha type, 1
QTRT1	-1.44	81890	QTRT1	queuine tRNA-ribosyltransferase 1
RNF14	-1.44	9604	RNF14	ring finger protein 14
WDR12	-1.44	55759	WDR12	WD repeat domain 12
C21ORF45	-1.44	54069	C21ORF45	chromosome 21 open reading frame 45
FLJ21908	-1.44	79657	RPAP3	RNA polymerase II associated protein 3
MRCL3	-1.44	10627	MYL12A	myosin, light chain 12A, regulatory, non-sarcomeric
ANAPC13	-1.45	25847	ANAPC13	anaphase promoting complex subunit 13
ABCE1	-1.45	6059	ABCE1	ATP-binding cassette, sub-family E (OABP), member 1
BLOC1S1	-1.45	2647	BLOC1S1	biogenesis of lysosomal organelles complex-1, subunit 1
CCNK	-1.45	8812	CCNK	cyclin K
MRPL54	-1.45	116541	MRPL54	mitochondrial ribosomal protein L54
COMMD3	-1.46	23412	COMMD3	COMM domain containing 3
FAM73A	-1.46	374986	FAM73A	family with sequence similarity 73, member A
HIGD2A	-1.46	192286	HIGD2A	HIG1 hypoxia inducible domain family, member 2A
MRPL41	-1.46	64975	MRPL41	mitochondrial ribosomal protein L41
MRPL46	-1.46	26589	MRPL46	mitochondrial ribosomal protein L46
NOLC1	-1.46	9221	NOLC1	nucleolar and coiled-body phosphoprotein 1
NOC3L	-1.46	64318	NOC3L	nucleolar complex associated 3 homolog (S. cerevisiae)
PEX5	-1.46	5830	PEX5	peroxisomal biogenesis factor 5
PIR	-1.46	8544	PIR	pirin (iron-binding nuclear protein)
PTGES	-1.46	9536	PTGES	prostaglandin E synthase
RND3	-1.46	390	RND3	Rho family GTPase 3
RPL26L1	-1.46	51121	RPL26L1	ribosomal protein L26-like 1
RPS4X	-1.46	6191	RPS4X	ribosomal protein S4, X-linked
RRN3	-1.46	54700	RRN3	RRN3 RNA polymerase I transcription factor homolog (S. cerevisiae)
TBC1D22A	-1.46	25771	TBC1D22A	TBC1 domain family, member 22A
VRK1	-1.46	7443	VRK1	vaccinia related kinase 1
C20ORF7	-1.46	79133	C20ORF7	chromosome 20 open reading frame 7
HSPC117	-1.46	51493	C22orf28	chromosome 22 open reading frame 28
MGC33212	-1.46	255758	TCTEX1D2	Tctex1 domain containing 2

CKS2	-1.47	1164	CKS2	CDC28 protein kinase regulatory subunit 2
CHCHD8	-1.47	51287	CHCHD8	coiled-coil-helix-coiled-coil-helix domain containing 8
METAP2	-1.47	10988	METAP2	methionyl aminopeptidase 2
MRPS16	-1.47	51021	MRPS16	mitochondrial ribosomal protein S16
OBFC2A	-1.47	64859	OBFC2A	oligonucleotide/oligosaccharide-binding fold containing 2A
SAV1	-1.47	60485	SAV1	salvador homolog 1 (Drosophila)
HSPC111	-1.47	51491	NOP16	NOP16 nucleolar protein homolog (yeast)
NT5C3	-1.48	51251	NT5C3	5'-nucleotidase, cytosolic III
BCCIP	-1.48	56647	BCCIP	BRCA2 and CDKN1A interacting protein
BYSL	-1.48	705	BYSL	bystin-like
CIRBP	-1.48	1153	CIRBP	cold inducible RNA binding protein
EMP1	-1.48	2012	EMP1	epithelial membrane protein 1
GLA	-1.48	2717	GLA	galactosidase, alpha
GTF2IRD2	-1.48	84163	GTF2IRD2	GTF2I repeat domain containing 2
KRT8	-1.48	3856	KRT8	keratin 8
LRRFIP2	-1.48	9209	LRRFIP2	leucine rich repeat (in FLII) interacting protein 2
MRPS21	-1.48	54460	MRPS21	mitochondrial ribosomal protein S21
NUDC	-1.48	10726	NUDC	nuclear distribution gene C homolog (A. nidulans)
SAAL1	-1.48	113174	SAAL1	serum amyloid A-like 1
SRP72	-1.48	6731	SRP72	signal recognition particle 72kDa
SNRPG	-1.48	6637	SNRPG	small nuclear ribonucleoprotein polypeptide G
VPS29	-1.48	51699	VPS29	vacuolar protein sorting 29 homolog (S. cerevisiae)
C15ORF15	-1.48	51187	RSL24D1	ribosomal L24 domain containing 1
C19ORF33	-1.48	64073	C19orf33	chromosome 19 open reading frame 33
HSPC176	-1.48	51693	TRAPPC2L	trafficking protein particle complex 2-like
PSARL	-1.48	55486	PARL	presenilin associated, rhomboid-like
BTF3	-1.49	689	BTF3	basic transcription factor 3
COPS3	-1.49	8533	COPS3	COP9 constitutive photomorphogenic homolog subunit 3 (Arabidopsis)
FJX1	-1.49	24147	FJX1	four jointed box 1 (Drosophila)
GRPEL1	-1.49	80273	GRPEL1	GrpE-like 1, mitochondrial (E. coli)
MRPL18	-1.49	29074	MRPL18	mitochondrial ribosomal protein L18
PLAU	-1.49	5328	PLAU	plasminogen activator, urokinase
RBM28	-1.49	55131	RBM28	RNA binding motif protein 28
SERPINB1	-1.49	1992	SERPINB1	serpin peptidase inhibitor, clade B (ovalbumin), member 1
LTB4DH	-1.49	22949	PTGR1	prostaglandin reductase 1
ATP5O	-1.50	539	ATP5O	(oligomycin sensitivity conferring protein)
CSNK2B	-1.50	1460	CSNK2B	casein kinase 2, beta polypeptide
C10ORF65	-1.50	112817	DHDPSL	dihydrodipicolinate synthase-like, mitochondrial
SELI	-1.50	85465	EPT1	ethanolaminephosphotransferase 1 (CDP-ethanolamine-specific)
GLRX	-1.50	2745	GLRX	glutaredoxin (thioltransferase)
IVNS1ABP	-1.50	10625	IVNS1ABP	influenza virus NS1A binding protein
KIAA0020	-1.50	9933	KIAA0020	KIAA0020
MRPL20	-1.50	55052	MRPL20	mitochondrial ribosomal protein L20
PEF1	-1.50	553115	PEF1	penta-EF-hand domain containing 1
RPL4	-1.50	6124	RPL4	ribosomal protein L4
RPL7L1	-1.50	285855	RPL7L1	ribosomal protein L7-like 1

TTC4	-1.50	7268	TTC4	tetratricopeptide repeat domain 4
TRFP	-1.50	9477	TRFP	(Drosophila)
WEE1	-1.50	7465	WEE1	WEE1 homolog (S. pombe)
C21ORF70	-1.51	85395	C21ORF70	chromosome 21 open reading frame 70
COX6B1	-1.51	1340	COX6B1	cytochrome c oxidase subunit Vib polypeptide 1 (ubiquitous)
FHL2	-1.51	2274	FHL2	four and a half LIM domains 2
HEBP1	-1.51	50865	HEBP1	heme binding protein 1
MDH1	-1.51	4190	MDH1	malate dehydrogenase 1, NAD (soluble)
6	-1.51	10200	MPHOSPH6	M-phase phosphoprotein 6
PURB	-1.51	5814	PURB	purine-rich element binding protein B
RNUXA	-1.51	51808	RNUXA	RNA U, small nuclear RNA export adaptor (phosphorylation regulated)
TRIP4	-1.51	9325	TRIP4	thyroid hormone receptor interactor 4
ZCCHC9	-1.51	84240	ZCCHC9	zinc finger, CCHC domain containing 9
ACP6	-1.52	51205	ACP6	acid phosphatase 6, lysophosphatidic
ARPC5	-1.52	10092	ARPC5	actin related protein 2/3 complex, subunit 5, 16kDa
BLES03	-1.52	83638	C11orf68	chromosome 11 open reading frame 68
GNE	-1.52	10020	GNE	kinase
IMPDH2	-1.52	3615	IMPDH2	IMP (inosine monophosphate) dehydrogenase 2
MRPL1	-1.52	65008	MRPL1	mitochondrial ribosomal protein L1
NDUFA7	-1.52	4701	NDUFA7	NADH dehydrogenase (ubiquinone) 1 alpha subcomplex, 7, 14.5kDa
RPS21	-1.52	6227	RPS21	ribosomal protein S21
TIMM9	-1.52	26520	TIMM9	translocase of inner mitochondrial membrane 9 homolog (yeast)
TOMM7	-1.52	54543	TOMM7	translocase of outer mitochondrial membrane 7 homolog (yeast)
CD55	-1.53	1604	CD55	blood group)
EIF4A2	-1.53	1974	EIF4A2	eukaryotic translation initiation factor 4A, isoform 2
KIAA0101	-1.53	9768	KIAA0101	KIAA0101
MAP3K7	-1.53	6885	MAP3K7	mitogen-activated protein kinase kinase kinase 7
MIMITIN	-1.53	91942	NDUFAF2	factor 2
NIP7	-1.53	51388	NIP7	nuclear import 7 homolog (S. cerevisiae)
PHF14	-1.53	9678	PHF14	PHD finger protein 14
RPS13	-1.53	6207	RPS13	ribosomal protein S13
TIPRL	-1.53	261726	TIPRL	TIP41, TOR signalling pathway regulator-like (S. cerevisiae)
ZFAND1	-1.53	79752	ZFAND1	zinc finger, AN1-type domain 1
ANXA2	-1.54	302	ANXA2	annexin A2
ATP5H	-1.54	10476	ATP5H	ATP synthase, H+ transporting, mitochondrial F0 complex, subunit d
ATP5I	-1.54	521	ATP5I	ATP synthase, H+ transporting, mitochondrial F0 complex, subunit E
BCL2L1	-1.54	598	BCL2L1	BCL2-like 1
C12ORF11	-1.54	55726	C12orf11	chromosome 12 open reading frame 11
C6ORF125	-1.54	84300	C6orf125	chromosome 6 open reading frame 125
FAF1	-1.54	11124	FAF1	Fas (TNFRSF6) associated factor 1
GFM1	-1.54	85476	GFM1	G elongation factor, mitochondrial 1
LSM5	-1.54	23658	LSM5	LSM5 homolog, U6 small nuclear RNA associated (S. cerevisiae)
OBFC1	-1.54	79991	OBFC1	oligonucleotide/oligosaccharide-binding fold containing 1
SUGT1	-1.54	10910	SUGT1	SGT1, suppressor of G2 allele of SKP1 (S. cerevisiae)
GALE	-1.54	2582	GALE	UDP-galactose-4-epimerase
ALDH1A3	-1.55	220	ALDH1A3	aldehyde dehydrogenase 1 family, member A3

CD24	-1.55	934	CD24	CD24 molecule
DCAKD	-1.55	79877	DCAKD	dephospho-CoA kinase domain containing
EDF1	-1.55	8721	EDF1	endothelial differentiation-related factor 1
IL6	-1.55	3569	IL6	interleukin 6 (interferon, beta 2)
KPNA3	-1.55	3839	KPNA3	karyopherin alpha 3 (importin alpha 4)
STOML2	-1.55	30968	STOML2	stomatin (EPB72)-like 2
FAM82B	-1.56	51115	FAM82B	family with sequence similarity 82, member B
IMMP2L	-1.56	83943	IMMP2L	IMP2 inner mitochondrial membrane peptidase-like (S. cerevisiae)
MELK	-1.56	9833	MELK	maternal embryonic leucine zipper kinase
NOLA2	-1.56	55651	NOLA2	nucleolar protein family A, member 2 (H/ACA small nucleolar RNPs)
POLE4	-1.56	56655	POLE4	polymerase (DNA-directed), epsilon 4 (p12 subunit)
PHB	-1.56	5245	PHB	prohibitin
RPL39L	-1.56	116832	RPL39L	ribosomal protein L39-like
TSNAX	-1.56	7257	TSNAX	translin-associated factor X
AFMID	-1.57	125061	AFMID	arylformamidase
C18ORF25	-1.57	389541	C7orf59	chromosome 7 open reading frame 59
CXORF26	-1.57	51260	CXorf26	chromosome X open reading frame 26
HFL	-1.57	3078	CFHR1	complement factor H-related 1
MGC61571	-1.57	152100	CMC1	COX assembly mitochondrial protein homolog (S. cerevisiae)
FAM82C	-1.57	55177	FAM82C	family with sequence similarity 82, member C
ICT1	-1.57	3396	ICT1	immature colon carcinoma transcript 1
ISOC1	-1.57	51015	ISOC1	isochorismatase domain containing 1
LASS2	-1.57	29956	LASS2	LAG1 homolog, ceramide synthase 2 (S. cerevisiae)
GNPTAB	-1.57	79158	GNPTAB	N-acetylglucosamine-1-phosphate transferase, alpha and beta subunits
PPIH	-1.57	10465	PPIH	peptidylprolyl isomerase H (cyclophilin H)
PCNP	-1.57	57092	PCNP	PEST proteolytic signal containing nuclear protein
PDCL3	-1.57	79031	PDCL3	phosducin-like 3
PFDN6	-1.57	10471	PFDN6	prefoldin subunit 6
RHOB	-1.57	388	RHOB	ras homolog gene family, member B
APOBEC3B	-1.58	9582	APOBEC3B	apolipoprotein B mRNA editing enzyme, catalytic polypeptide-like 3B
METTL3	-1.58	56339	METTL3	methyltransferase like 3
NACA	-1.58	4666	NACA	nascent-polypeptide-associated complex alpha polypeptide
NUP37	-1.58	79023	NUP37	nucleoporin 37kDa
PPIB	-1.58	5479	PPIB	peptidylprolyl isomerase B (cyclophilin B)
PRKRA	-1.58	8575	PRKRA	activator
RPL14	-1.58	9045	RPL14	ribosomal protein L14
LOC389541	-1.59	389541	C7orf59	chromosome 7 open reading frame 59
GLE1L	-1.59	2733	GLE1L	GLE1 RNA export mediator-like (yeast)
MRPS18C	-1.59	51023	MRPS18C	mitochondrial ribosomal protein S18C
NDUFA8	-1.59	4702	NDUFA8	NADH dehydrogenase (ubiquinone) 1 alpha subcomplex, 8, 19kDa
NSUN5	-1.59	55695	NSUN5	NOL1/NOP2/Sun domain family, member 5
TBC1D7	-1.59	51256	TBC1D7	TBC1 domain family, member 7
TOMM22	-1.59	56993	TOMM22	translocase of outer mitochondrial membrane 22 homolog (yeast)
APEX1	-1.60	328	APEX1	APEX nuclease (multifunctional DNA repair enzyme) 1
CAV1	-1.60	857	CAV1	caveolin 1, caveolae protein, 22kDa
CCDC72	-1.60	51372	CCDC72	coiled-coil domain containing 72

MAGOH	-1.60	4116	MAGOH	mago-nashi homolog, proliferation-associated (Drosophila)
MNAT1	-1.60	4331	MNAT1	menage a trois homolog 1, cyclin H assembly factor (Xenopus laevis)
MRPS30	-1.60	10884	MRPS30	mitochondrial ribosomal protein S30
MTIF2	-1.60	4528	MTIF2	mitochondrial translational initiation factor 2
RBM22	-1.60	55696	RBM22	RNA binding motif protein 22
BOLA3	-1.61	388962	BOLA3	bolA homolog 3 (E. coli)
CKLF	-1.61	51192	CKLF	chemokine-like factor
EBNA1BP2	-1.61	10969	EBNA1BP2	EBNA1 binding protein 2
EIF2A	-1.61	83939	EIF2A	eukaryotic translation initiation factor 2A, 65kDa
GANAB	-1.61	23193	GANAB	glucosidase, alpha; neutral AB
C6ORF49	-1.61	29964	PRICKLE4	ickle homolog 4 (Drosophila)
RABEPK	-1.61	10244	RABEPK	Rab9 effector protein with kelch motifs
RARS	-1.62	5917	RARS	arginyl-tRNA synthetase
ALG5	-1.62	29880	ALG5	phosphate beta-glucosyltransferase)
CBX1	-1.62	10951	CBX1	chromobox homolog 1 (HP1 beta homolog Drosophila)
CPNE8	-1.62	144402	CPNE8	copine VIII
KRT19	-1.62	3880	KRT19	keratin 19
MRPL9	-1.62	65005	MRPL9	mitochondrial ribosomal protein L9
NIT2	-1.62	56954	NIT2	nitrilase family, member 2
TRA16	-1.62	126382	NR2C2AP	nuclear receptor 2C2-associated protein
TM4SF1	-1.62	4071	TM4SF1	transmembrane 4 L six family member 1
YWHAG	-1.62	7532	YWHAG	protein, gamma polypeptide
AHNAK	-1.63	79026	AHNAK	AHNAK nucleoprotein (desmoyokin)
BXDC1	-1.63	84154	BXDC1	brix domain containing 1
C7ORF11	-1.63	136647	C7orf11	chromosome 7 open reading frame 11
C14ORF112	-1.63	51241	COX16	COX16 cytochrome c oxidase assembly homolog (S. cerevisiae)
FLJ14668	-1.63	84908	FAM136A	family with sequence similarity 136, member A
FLJ14803	-1.63	84908	FAM136A	family with sequence similarity 136, member A
MYADM	-1.63	91663	MYADM	myeloid-associated differentiation marker
POLR2G	-1.63	5436	POLR2G	polymerase (RNA) II (DNA directed) polypeptide G
WDSOF1	-1.63	25879	WDSOF1	WD repeats and SOF1 domain containing
ZFP91	-1.63	80829	ZFP91	zinc finger protein 91 homolog (mouse)
ZBED2	-1.63	79413	ZBED2	zinc finger, BED-type containing 2
C14ORF156	-1.64	81892	C14orf156	chromosome 14 open reading frame 156
DSCR2	-1.64	8624	DSCR2	Down syndrome critical region gene 2
MRPS31	-1.64	10240	MRPS31	mitochondrial ribosomal protein S31
NDUFB6	-1.64	4712	NDUFB6	NADH dehydrogenase (ubiquinone) 1 beta subcomplex, 6, 17kDa
PLSCR1	-1.64	5359	PLSCR1	phospholipid scramblase 1
PTD015	-1.65	28971	C11orf67	chromosome 11 open reading frame 67
EXOSC5	-1.65	56915	EXOSC5	exosome component 5
SLC5A6	-1.65	8884	SLC5A6	6
ATIC	-1.66	471	ATIC	formyltransferase/IMP cyclohydrolase
ACTG1	-1.66	71	ACTG1	actin, gamma 1
BAIAP2	-1.66	10458	BAIAP2	BAI1-associated protein 2
C11ORF48	-1.66	79081	C11ORF48	chromosome 11 open reading frame 48
DDX21	-1.66	9188	DDX21	DEAD (Asp-Glu-Ala-Asp) box polypeptide 21

E2F4	-1.66	1874	E2F4	E2F transcription factor 4, p107/p130-binding
MRPL51	-1.66	51258	MRPL51	mitochondrial ribosomal protein L51
MRPS22	-1.66	56945	MRPS22	mitochondrial ribosomal protein S22
NDUFB9	-1.66	4715	NDUFB9	NADH dehydrogenase (ubiquinone) 1 beta subcomplex, 9, 22kDa
PQLC3	-1.66	130814	PQLC3	PQ loop repeat containing 3
CSNK1D	-1.67	1453	CSNK1D	casein kinase 1, delta
CSTB	-1.67	1476	CSTB	cystatin B (stefin B)
RPIA	-1.67	22934	RPIA	ribose 5-phosphate isomerase A (ribose 5-phosphate epimerase)
YIPF1	-1.67	54432	YIPF1	Yip1 domain family, member 1
ACN9	-1.68	57001	ACN9	ACN9 homolog (S. cerevisiae)
C12ORF10	-1.68	60314	C12ORF10	chromosome 12 open reading frame 10
EIF2B2	-1.68	8892	EIF2B2	eukaryotic translation initiation factor 2B, subunit 2 beta, 39kDa
GAL	-1.68	51083	GAL	galanin
MRPL50	-1.68	54534	MRPL50	mitochondrial ribosomal protein L50
RPL36AL	-1.68	6166	RPL36AL	ribosomal protein L36a-like
UIP1	-1.69	55559	HAUS7	HAUS augmin-like complex, subunit 7
PGM2L1	-1.69	283209	PGM2L1	phosphoglucomutase 2-like 1
RRS1	-1.69	23212	RRS1	RRS1 ribosome biogenesis regulator homolog (S. cerevisiae)
THYN1	-1.69	29087	THYN1	thymocyte nuclear protein 1
GLS	-1.70	2744	GLS	glutaminase
UGT2B11	-1.70	10720	UGT2B11	UDP glucuronosyltransferase 2 family, polypeptide B11
ARD1A	-1.71	8260	ARD1A	ARD1 homolog A, N-acetyltransferase (S. cerevisiae)
BXDC2	-1.71	55299	BXDC2	brix domain containing 2
C21ORF25	-1.71	25966	C2CD2	C2 calcium-dependent domain containing 2
MRPL21	-1.71	219927	MRPL21	mitochondrial ribosomal protein L21
PYGL	-1.71	5836	PYGL	type VI)
PCSK5	-1.71	5125	PCSK5	proprotein convertase subtilisin/kexin type 5
PSMB5	-1.71	5693	PSMB5	proteasome (prosome, macropain) subunit, beta type, 5
B4GALT4	-1.71	8702	B4GALT4	UDP-Gal:betaGlcNAc beta 1,4- galactosyltransferase, polypeptide 4
C1ORF181	-1.71	54680	ZNHIT6	zinc finger, HIT type 6
ADI1	-1.72	55256	ADI1	acireductone dioxygenase 1
DNM3	-1.72	26052	DNM3	dynamin 3
CNN3	-1.73	1266	CNN3	calponin 3, acidic
CYCS	-1.73	54205	CYCS	cytochrome c, somatic
METTL5	-1.73	29081	METTL5	methyltransferase like 5
NOLA3	-1.73	55505	NOLA3	nucleolar protein family A, member 3 (H/ACA small nucleolar RNPs)
OSTF1	-1.73	26578	OSTF1	osteoclast stimulating factor 1
SH3BGRL2	-1.73	83699	SH3BGRL2	SH3 domain binding glutamic acid-rich protein like 2
VDAC2	-1.73	7417	VDAC2	voltage-dependent anion channel 2
CCDC23	-1.74	374969	CCDC23	coiled-coil domain containing 23
GLO1	-1.74	2739	GLO1	glyoxalase I
TMEM16A	-1.74	55107	TMEM16A	transmembrane protein 16A
UPK1B	-1.74	7348	UPK1B	uroplakin 1B
COMMD1	-1.75	150684	COMMD1	copper metabolism (Murr1) domain containing 1
DEFB4	-1.75	1673	DEFB4	defensin, beta 4
ITGB4BP	-1.75	3692	ITGB4BP	integrin beta 4 binding protein

LOC144363	-1.75	144363	LYRM5	LYR motif containing 5
NDUFA12	-1.75	55967	NDUFA12	NADH dehydrogenase (ubiquinone) 1 alpha subcomplex, 12
P4HA2	-1.75	8974	P4HA2	hydroxylase), alpha polypeptide II
PCCB	-1.76	5096	PCCB	propionyl Coenzyme A carboxylase, beta polypeptide
CXCR4	-1.77	7852	CXCR4	chemokine (C-X-C motif) receptor 4
DRG1	-1.77	4733	DRG1	developmentally regulated GTP binding protein 1
GLDC	-1.77	2731	GLDC	glycine dehydrogenase (decarboxylating)
LOC144501	-1.77	144501	KRT80	keratin 80
PLEC1	-1.77	5339	PLEC1	plectin 1, intermediate filament binding protein 500kDa
PDCD2	-1.77	5134	PDCD2	programmed cell death 2
MRPL33	-1.78	9553	MRPL33	mitochondrial ribosomal protein L33
CHCHD4	-1.79	131474	CHCHD4	coiled-coil-helix-coiled-coil-helix domain containing 4
KCNS1	-1.79	3787	KCNS1	member 1
PSMA6	-1.79	5687	PSMA6	proteasome (prosome, macropain) subunit, alpha type, 6
MKRN2	-1.80	23609	MKRN2	makorin, ring finger protein, 2
MRPL48	-1.80	51642	MRPL48	mitochondrial ribosomal protein L48
C14ORF2	-1.81	9556	C14orf2	chromosome 14 open reading frame 2
DCBLD2	-1.81	131566	DCBLD2	discoidin, CUB and LCCL domain containing 2
MRPL22	-1.81	29093	MRPL22	mitochondrial ribosomal protein L22
SF3B5	-1.81	83443	SF3B5	splicing factor 3b, subunit 5, 10kDa
CLEC2D	-1.82	29121	CLEC2D	C-type lectin domain family 2, member D
EIF3S7	-1.82	8664	EIF3S7	eukaryotic translation initiation factor 3, subunit 7 zeta, 66/67kDa
PMPCB	-1.82	9512	PMPCB	peptidase (mitochondrial processing) beta
PA2G4	-1.82	5036	PA2G4	proliferation-associated 2G4, 38kDa
A	-1.82	51330	TNFRSF12A	tumor necrosis factor receptor superfamily, member 12A
ANXA1	-1.84	301	ANXA1	annexin A1
RPS15	-1.84	6209	RPS15	ribosomal protein S15
UGT2B7	-1.84	7364	UGT2B7	UDP glucuronosyltransferase 2 family, polypeptide B7
ALDH7A1	-1.85	501	ALDH7A1	aldehyde dehydrogenase 7 family, member A1
EXOSC3	-1.85	51010	EXOSC3	exosome component 3
SRPR	-1.85	6734	SRPR	signal recognition particle receptor ('docking protein')
C1QBP	-1.87	708	C1QBP	complement component 1, q subcomponent binding protein
IRF8	-1.87	3394	IRF8	interferon regulatory factor 8
GTF2E2	-1.88	2961	GTF2E2	general transcription factor IIE, polypeptide 2, beta 34kDa
RPL24	-1.88	6152	RPL24	ribosomal protein L24
UQCRC2	-1.88	7385	UQCRC2	ubiquinol-cytochrome c reductase core protein II
CAV2	-1.89	858	CAV2	caveolin 2
S100A10	-1.89	6281	S100A10	S100 calcium binding protein A10
CDC23	-1.90	8697	CDC23	CDC23 (cell division cycle 23, yeast, homolog)
POLR3C	-1.90	10623	POLR3C	polymerase (RNA) III (DNA directed) polypeptide C (62kD)
ZMPSTE24	-1.90	10269	ZMPSTE24	zinc metallopeptidase (STE24 homolog, yeast)
ANXA3	-1.91	306	ANXA3	annexin A3
C3ORF26	-1.91	84319	C3orf26	chromosome 3 open reading frame 26
COL4A2	-1.91	1284	COL4A2	collagen, type IV, alpha 2
SNRPF	-1.91	6636	SNRPF	small nuclear ribonucleoprotein polypeptide F
STAMBP	-1.91	10617	STAMBP	STAM binding protein

SDHD	-1.91	6392	SDHD	protein
CTSL2	-1.92	1515	CTSL2	cathepsin L2
CTHRC1	-1.92	115908	CTHRC1	collagen triple helix repeat containing 1
DUSP3	-1.92	1845	DUSP3	related)
GTF2H5	-1.93	404672	GTF2H5	general transcription factor IIH, polypeptide 5
PLAC8	-1.93	51316	PLAC8	placenta-specific 8
TOMM20	-1.93	9804	TOMM20	translocase of outer mitochondrial membrane 20 homolog (yeast)
VBP1	-1.93	7411	VBP1	von Hippel-Lindau binding protein 1
LSM3	-1.94	27258	LSM3	LSM3 homolog, U6 small nuclear RNA associated (S. cerevisiae)
PTRF	-1.94	284119	PTRF	polymerase I and transcript release factor
EIF2B1	-1.97	1967	EIF2B1	eukaryotic translation initiation factor 2B, subunit 1 alpha, 26kDa
C9ORF46	-1.98	55848	C9orf46	chromosome 9 open reading frame 46
LAMA3	-1.99	3909	LAMA3	laminin, alpha 3
QPRT	-2.01	23475	QPRT	pyrophosphorylase (carboxylating))
BCAT2	-2.02	587	BCAT2	branched chain aminotransferase 2, mitochondrial
MRPL39	-2.02	54148	MRPL39	mitochondrial ribosomal protein L39
ATP5G1	-2.03	516	ATP5G1	(subunit 9)
RPP40	-2.03	10799	RPP40	ribonuclease P 40kDa subunit
ZMYM6	-2.05	9204	ZMYM6	zinc finger, MYM-type 6
C1ORF57	-2.16	84284	C1orf57	chromosome 1 open reading frame 57
DPM3	-2.17	54344	DPM3	dolichyl-phosphate mannosyltransferase polypeptide 3
NNMT	-2.17	4837	NNMT	nicotinamide N-methyltransferase
TSPAN12	-2.23	23554	TSPAN12	tetraspanin 12
FLG	-2.26	2312	FLG	filaggrin
SCEL	-2.26	8796	SCEL	sciellin
LIMA1	-2.30	51474	LIMA1	LIM domain and actin binding 1
RSL1D1	-2.30	26156	RSL1D1	ribosomal L1 domain containing 1
RPL34	-2.30	6164	RPL34	ribosomal protein L34
MGC20255	-2.33	90324	CCDC97	coiled-coil domain containing 97
C10ORF42	-2.34	90550	CCDC109A	coiled-coil domain containing 109A
MRPS17	-2.36	51373	MRPS17	mitochondrial ribosomal protein S17
LTA4H	-2.37	4048	LTA4H	leukotriene A4 hydrolase
RPL29	-2.40	6159	RPL29	ribosomal protein L29
MRPL40	-2.41	64976	MRPL40	mitochondrial ribosomal protein L40
RPS7	-2.54	6201	RPS7	ribosomal protein S7
FOSL1	-2.57	8061	FOSL1	FOS-like antigen 1
FAM79A	-2.59	127262	FAM79A	family with sequence similarity 79, member A
CCNA1	-2.62	8900	CCNA1	cyclin A1
CTGF	-2.63	1490	CTGF	connective tissue growth factor
FOLR3	-2.65	2352	FOLR3	folate receptor 3 (gamma)
MRPS15	-2.73	64960	MRPS15	mitochondrial ribosomal protein S15
TNFSF5IP1	-2.80	56984	TNFSF5IP1	tumor necrosis factor superfamily, member 5-induced protein 1
SYTL2	-2.81	54843	SYTL2	synaptotagmin-like 2
PRSS23	-2.90	11098	PRSS23	protease, serine, 23
SF3B14	-2.92	51639	SF3B14	splicing factor 3B, 14 kDa subunit
TINAGL1	-2.94	64129	TINAGL1	tubulointerstitial nephritis antigen-like 1

TPK1	-2.97	27010	TPK1	thiamin pyrophosphokinase 1
RRAS2	-2.99	22800	RRAS2	related RAS viral (r-ras) oncogene homolog 2
FLJ20625	-3.11	55004	C11orf59	chromosome 11 open reading frame 59
TINP1	-3.95	10412	NSA2	NSA2 ribosome biogenesis homolog (S. cerevisiae)
NMU	-5.74	10874	NMU	neuromedin U
CLDN7	-6.97	1366	CLDN7	claudin 7
**Significant gene list in OVCA420**				
**Input ID**	**Fold**	**Gene ID**	**Symbol**	**Name**
CX3CL1	3.44	6376	CX3CL1	chemokine (C-X3-C motif) ligand 1
PANX2	3.36	56666	PANX2	pannexin 2
SLC25A22	3.18	79751	SLC25A22	solute carrier family 25 (mitochondrial carrier: glutamate), member 22
MRPL36	3.06	64979	MRPL36	mitochondrial ribosomal protein L36
APLP2	3.02	334	APLP2	amyloid beta (A4) precursor-like protein 2
DKK1	2.87	22943	DKK1	dickkopf homolog 1 (Xenopus laevis)
AGPAT5	2.80	55326	AGPAT5	acyltransferase, epsilon)
NRG1	2.69	3084	NRG1	neuregulin 1
IL8	2.66	3576	IL8	interleukin 8
NDRG4	2.61	65009	NDRG4	NDRG family member 4
HRASLS3	2.58	11145	HRASLS3	HRAS-like suppressor 3
FADS3	2.53	3995	FADS3	fatty acid desaturase 3
LYSMD2	2.48	256586	LYSMD2	LysM, putative peptidoglycan-binding, domain containing 2
PPP2R2C	2.47	5522	PPP2R2C	gamma isoform
LOC203547	2.45	203547	VMA21	VMA21 vacuolar H+-ATPase homolog (S. cerevisiae)
TMCO1	2.43	54499	TMCO1	transmembrane and coiled-coil domains 1
DNAJB6	2.41	10049	DNAJB6	DnaJ (Hsp40) homolog, subfamily B, member 6
NUDT21	2.40	11051	NUDT21	nudix (nucleoside diphosphate linked moiety X)-type motif 21
C1S	2.36	716	C1S	complement component 1, s subcomponent
MAP1LC3B	2.34	81631	MAP1LC3B	microtubule-associated protein 1 light chain 3 beta
SCARB1	2.34	949	SCARB1	scavenger receptor class B, member 1
EEF1A2	2.29	1917	EEF1A2	eukaryotic translation elongation factor 1 alpha 2
A01247	2.27	22998	LIMCH1	LIM and calponin homology domains 1
ZCCHC14	2.27	23174	ZCCHC14	zinc finger, CCHC domain containing 14
RKHD1	2.26	399664	RKHD1	ring finger and KH domain containing 1
F5	2.23	2153	F5	coagulation factor V (proaccelerin, labile factor)
RNF149	2.23	284996	RNF149	ring finger protein 149
ZNF614	2.23	80110	ZNF614	zinc finger protein 614
PPIA	2.19	5478	PPIA	peptidylprolyl isomerase A (cyclophilin A)
PTPNS1	2.17	140885	SIRPA	signal-regulatory protein alpha
CXADR	2.15	1525	CXADR	coxsackie virus and adenovirus receptor
MAPK6	2.15	5597	MAPK6	mitogen-activated protein kinase 6
SLC35B1	2.14	10237	SLC35B1	solute carrier family 35, member B1
DNM1L	2.13	10059	DNM1L	dynamin 1-like
SERP1	2.13	27230	SERP1	stress-associated endoplasmic reticulum protein 1

C17ORF63	2.11	55731	C17orf63	chromosome 17 open reading frame 63
PHGDH	2.10	26227	PHGDH	phosphoglycerate dehydrogenase
CKAP4	2.09	10970	CKAP4	cytoskeleton-associated protein 4
PLAU	2.09	5328	PLAU	plasminogen activator, urokinase
SET	2.09	6418	SET	SET translocation (myeloid leukemia-associated)
IMPAD1	2.08	54928	IMPAD1	inositol monophosphatase domain containing 1
SERPINB7	2.07	8710	SERPINB7	serpin peptidase inhibitor, clade B (ovalbumin), member 7
NETO2	2.04	81831	NETO2	neuropilin (NRP) and tolloid (TLL)-like 2
GRINA	2.03	2907	GRINA	1 (glutamate binding)
MT1A	2.03	4489	MT1A	metallothionein 1A (functional)
KIAA0152	2.02	9761	KIAA0152	KIAA0152
MT2A	2.02	4502	MT2A	metallothionein 2A
RAN	2.02	5901	RAN	RAN, member RAS oncogene family
FYCO1	2.01	79443	FYCO1	FYVE and coiled-coil domain containing 1
RAB6IP1	2.00	23258	RAB6IP1	RAB6 interacting protein 1
SURF4	2.00	6836	SURF4	surfeit 4
GALGT	1.99	124872	B4GALNT2	beta-1,4-N-acetyl-galactosaminyl transferase 2
DBN1	1.99	1627	DBN1	drebrin 1
SLC39A6	1.99	25800	SLC39A6	solute carrier family 39 (zinc transporter), member 6
TEAD2	1.99	8463	TEAD2	TEA domain family member 2
BTG3	1.98	10950	BTG3	BTG family, member 3
OBFC2A	1.98	64859	OBFC2A	oligonucleotide/oligosaccharide-binding fold containing 2A
CREG1	1.97	8804	CREG1	cellular repressor of E1A-stimulated genes 1
IFI16	1.95	3428	IFI16	interferon, gamma-inducible protein 16
TPP1	1.95	1200	TPP1	tripeptidyl peptidase I
DDEF1	1.94	50807	DDEF1	development and differentiation enhancing factor 1
MALL	1.94	7851	MALL	mal, T-cell differentiation protein-like
CA12	1.92	771	CA12	carbonic anhydrase XII
HPRT1	1.92	3251	HPRT1	hypoxanthine phosphoribosyltransferase 1 (Lesch-Nyhan syndrome)
MT1X	1.92	4501	MT1X	metallothionein 1X
ZFP36L1	1.92	677	ZFP36L1	zinc finger protein 36, C3H type-like 1
LAMB1	1.91	3912	LAMB1	laminin, beta 1
RSNL2	1.91	79745	RSNL2	restin-like 2
SERPINA3	1.91	12	SERPINA3	member 3
FSCN1	1.89	6624	FSCN1	purpuratus)
MET	1.89	4233	MET	met proto-oncogene (hepatocyte growth factor receptor)
PRNP	1.88	5621	PRNP	Scheinker syndrome, fatal familial insomnia)
CDK6	1.87	1021	CDK6	cyclin-dependent kinase 6
CBX2	1.86	84733	CBX2	chromobox homolog 2 (Pc class homolog, Drosophila)
C3ORF21	1.86	152002	C3orf21	chromosome 3 open reading frame 21
PKMYT1	1.86	9088	PKMYT1	protein kinase, membrane associated tyrosine/threonine 1
E2F2	1.85	1870	E2F2	E2F transcription factor 2
E	1.85	27351	PPPDE2	PPPDE peptidase domain containing 2
ANAPC1	1.83	64682	ANAPC1	anaphase promoting complex subunit 1
GPT2	1.83	84706	GPT2	glutamic pyruvate transaminase (alanine aminotransferase) 2
KLHL21	1.83	9903	KLHL21	kelch-like 21 (Drosophila)

SERPINE2	1.83	5270	SERPINE2	inhibitor type 1), member 2
SLC12A8	1.83	84561	SLC12A8	solute carrier family 12 (potassium/chloride transporters), member 8
GALNT4	1.83	8693	GALNT4	acetylgalactosaminyltransferase 4 (GalNAc-T4)
GSR	1.82	2936	GSR	glutathione reductase
NOP5	1.82	51602	NOP58	NOP58 ribonucleoprotein homolog (yeast)
ASNS	1.81	440	ASNS	asparagine synthetase
GABPB2	1.80	2553	GABPB2	GA binding protein transcription factor, beta subunit 2
PIK3R2	1.80	5296	PIK3R2	phosphoinositide-3-kinase, regulatory subunit 2 (p85 beta)
PTPLA	1.79	9200	PTPLA	member A
SPIRE1	1.79	56907	SPIRE1	spire homolog 1 (Drosophila)
STS	1.79	412	STS	steroid sulfatase (microsomal), arylsulfatase C, isozyme S
DAZAP2	1.78	9802	DAZAP2	DAZ associated protein 2
GULP1	1.78	51454	GULP1	GULP, engulfment adaptor PTB domain containing 1
CDK2AP1	1.77	8099	CDK2AP1	CDK2-associated protein 1
CCL20	1.77	6364	CCL20	chemokine (C-C motif) ligand 20
CCNG1	1.77	900	CCNG1	cyclin G1
FADS1	1.77	3992	FADS1	fatty acid desaturase 1
NMD3	1.77	51068	NMD3	NMD3 homolog (S. cerevisiae)
PSAT1	1.77	29968	PSAT1	phosphoserine aminotransferase 1
SC65	1.77	10609	SC65	synaptonemal complex protein SC65
WDR4	1.77	10785	WDR4	WD repeat domain 4
NLF2	1.76	388125	C2CD4B	C2 calcium-dependent domain containing 4B
FBXO21	1.76	23014	FBXO21	F-box protein 21
LMBR1L	1.76	55716	LMBR1L	limb region 1 homolog (mouse)-like
RAPGEF1	1.76	2889	RAPGEF1	Rap guanine nucleotide exchange factor (GEF) 1
S100A8	1.76	6279	S100A8	S100 calcium binding protein A8
PRSS15	1.75	9361	LONP1	lon peptidase 1, mitochondrial
NRCAM	1.75	4897	NRCAM	neuronal cell adhesion molecule
RNASEH1	1.75	246243	RNASEH1	ribonuclease H1
UBE2D3	1.75	7323	UBE2D3	ubiquitin-conjugating enzyme E2D 3 (UBC4/5 homolog, yeast)
BRPF3	1.74	27154	BRPF3	bromodomain and PHD finger containing, 3
FAM38A	1.74	9780	FAM38A	family with sequence similarity 38, member A
MLLT6	1.74	4302	MLLT6	Drosophila); translocated to, 6
HMFN0839	1.73	84803	AGPAT9	1-acylglycerol-3-phosphate O-acyltransferase 9
C9ORF30	1.73	91283	C9ORF30	chromosome 9 open reading frame 30
CRY1	1.73	1407	CRY1	cryptochrome 1 (photolyase-like)
MLLT11	1.73	10962	MLLT11	Drosophila); translocated to, 11
TMEM38B	1.73	55151	TMEM38B	transmembrane protein 38B
ZDHHC14	1.73	79683	ZDHHC14	zinc finger, DHHC-type containing 14
HYOU1	1.72	10525	HYOU1	hypoxia up-regulated 1
KLHDC5	1.72	57542	KLHDC5	kelch domain containing 5
MLF2	1.72	8079	MLF2	myeloid leukemia factor 2
FLJ31196	1.72	146802	SLC47A2	solute carrier family 47, member 2
TMCO3	1.72	55002	TMCO3	transmembrane and coiled-coil domains 3
TUBA1	1.72	7277	TUBA1	tubulin, alpha 1
TP73L	1.72	8626	TP73L	tumor protein p73-like

RAF1	1.72	5894	RAF1	v-raf-1 murine leukemia viral oncogene homolog 1
WDR40A	1.72	25853	WDR40A	WD repeat domain 40A
WNT7A	1.72	7476	WNT7A	wingless-type MMTV integration site family, member 7A
BRD3	1.71	8019	BRD3	bromodomain containing 3
XPO5	1.71	57510	XPO5	exportin 5
KRT20	1.71	54474	KRT20	keratin 20
MAP2K1	1.71	5604	MAP2K1	mitogen-activated protein kinase kinase 1
NPLOC4	1.71	55666	NPLOC4	nuclear protein localization 4 homolog (S. cerevisiae)
RAB7L1	1.71	8934	RAB7L1	RAB7, member RAS oncogene family-like 1
C20ORF18	1.71	10616	RBCK1	RanBP-type and C3HC4-type zinc finger containing 1
SEC61A1	1.71	29927	SEC61A1	Sec61 alpha 1 subunit (S. cerevisiae)
TOMM34	1.71	10953	TOMM34	translocase of outer mitochondrial membrane 34
WIPI1	1.71	55062	WIPI1	WD repeat domain, phosphoinositide interacting 1
CBS	1.70	875	CBS	cystathionine-beta-synthase
FLOT2	1.70	2319	FLOT2	flotillin 2
LFNG	1.70	3955	LFNG	lunatic fringe homolog (Drosophila)
IGF2BP2	1.69	10644	IGF2BP2	insulin-like growth factor 2 mRNA binding protein 2
RPS6KB1	1.69	6198	RPS6KB1	ribosomal protein S6 kinase, 70kDa, polypeptide 1
COL4A5	1.68	1287	COL4A5	collagen, type IV, alpha 5 (Alport syndrome)
NAP1L1	1.68	4673	NAP1L1	nucleosome assembly protein 1-like 1
ZNF544	1.68	27300	ZNF544	zinc finger protein 544
EPHX1	1.67	2052	EPHX1	epoxide hydrolase 1, microsomal (xenobiotic)
RAC1	1.67	5879	RAC1	binding protein Rac1)
SDCCAG3	1.67	10807	SDCCAG3	serologically defined colon cancer antigen 3
WNT5A	1.67	7474	WNT5A	wingless-type MMTV integration site family, member 5A
FLJ11259	1.66	55332	DRAM1	DNA-damage regulated autophagy modulator 1
LIMK1	1.66	3984	LIMK1	LIM domain kinase 1
RRAGA	1.66	10670	RRAGA	Ras-related GTP binding A
BAMBI	1.65	25805	BAMBI	BMP and activin membrane-bound inhibitor homolog (Xenopus laevis)
CLNS1A	1.65	1207	CLNS1A	chloride channel, nucleotide-sensitive, 1A
CSK	1.65	1445	CSK	c-src tyrosine kinase
FECH	1.65	2235	FECH	ferrochelatase (protoporphyria)
FGFRL1	1.65	53834	FGFRL1	fibroblast growth factor receptor-like 1
IGF2R	1.65	3482	IGF2R	insulin-like growth factor 2 receptor
PIGM	1.65	93183	PIGM	phosphatidylinositol glycan anchor biosynthesis, class M
QSCN6L1	1.65	169714	QSCN6L1	quiescin Q6-like 1
C6ORF85	1.65	63027	SLC22A23	solute carrier family 22, member 23
ABCA1	1.64	19	ABCA1	ATP-binding cassette, sub-family A (ABC1), member 1
CTSH	1.64	1512	CTSH	cathepsin H
GJA1	1.64	2697	GJA1	gap junction protein, alpha 1, 43kDa (connexin 43)
HSPA8	1.64	3312	HSPA8	heat shock 70kDa protein 8
NINJ1	1.64	4814	NINJ1	ninjurin 1
ARHGAP21	1.64	57584	ARHGAP21	Rho GTPase activating protein 21
FJX1	1.63	24147	FJX1	four jointed box 1 (Drosophila)
SRPRB	1.63	58477	SRPRB	signal recognition particle receptor, B subunit
UGCG	1.63	7357	UGCG	UDP-glucose ceramide glucosyltransferase

GIT1	1.62	28964	GIT1	G protein-coupled receptor kinase interactor 1
LARP6	1.62	55323	LARP6	La ribonucleoprotein domain family, member 6
ALDH2	1.61	217	ALDH2	aldehyde dehydrogenase 2 family (mitochondrial)
COL17A1	1.61	1308	COL17A1	collagen, type XVII, alpha 1
SEPN1	1.61	57190	SEPN1	selenoprotein N, 1
ST3GAL5	1.61	8869	ST3GAL5	ST3 beta-galactoside alpha-2,3-sialyltransferase 5
ST13	1.61	6767	ST13	protein)
E2F3	1.60	1871	E2F3	E2F transcription factor 3
FOXJ3	1.60	22887	FOXJ3	forkhead box J3
KIAA1434	1.60	56261	GPCPD1	cerevisiae)
GLT25D1	1.60	79709	GLT25D1	glycosyltransferase 25 domain containing 1
MTHFD1L	1.60	25902	MTHFD1L	methylenetetrahydrofolate dehydrogenase (NADP+ dependent) 1-like
MSN	1.60	4478	MSN	moesin
STT3A	1.60	3703	STT3A	cerevisiae)
TUBB	1.60	203068	TUBB	tubulin, beta
ATP1B3	1.59	483	ATP1B3	ATPase, Na+/K+ transporting, beta 3 polypeptide
DDX39	1.59	10212	DDX39	DEAD (Asp-Glu-Ala-Asp) box polypeptide 39
DNASE2	1.59	1777	DNASE2	deoxyribonuclease II, lysosomal
GTF3A	1.59	2971	GTF3A	general transcription factor IIIA
GM2A	1.59	2760	GM2A	GM2 ganglioside activator
HNRPH1	1.59	3187	HNRPH1	heterogeneous nuclear ribonucleoprotein H1 (H)
ISG20L1	1.59	64782	ISG20L1	interferon stimulated exonuclease gene 20kDa-like 1
SLC38A2	1.59	54407	SLC38A2	solute carrier family 38, member 2
VANGL2	1.59	57216	VANGL2	vang-like 2 (van gogh, Drosophila)
HRAS	1.59	3265	HRAS	v-Ha-ras Harvey rat sarcoma viral oncogene homolog
CD47	1.58	961	CD47	CD47 molecule
CMKOR1	1.58	57007	CXCR7	chemokine (C-X-C motif) receptor 7
MGC14376	1.58	84981	C17orf91	chromosome 17 open reading frame 91
XPOT	1.58	11260	XPOT	exportin, tRNA (nuclear export receptor for tRNAs)
RHOQ	1.58	23433	RHOQ	ras homolog gene family, member Q
SH3BP4	1.58	23677	SH3BP4	SH3-domain binding protein 4
ZBED1	1.58	9189	ZBED1	zinc finger, BED-type containing 1
CEBPG	1.57	1054	CEBPG	CCAAT/enhancer binding protein (C/EBP), gamma
CLDN15	1.57	24146	CLDN15	claudin 15
C3ORF9	1.57	56983	KTELC1	KTEL (Lys-Tyr-Glu-Leu) containing 1
PVR	1.57	5817	PVR	poliovirus receptor
PSTPIP2	1.57	9050	PSTPIP2	proline-serine-threonine phosphatase interacting protein 2
RHOBTB3	1.57	22836	RHOBTB3	Rho-related BTB domain containing 3
UGT1A10	1.57	54575	UGT1A10	UDP glucuronosyltransferase 1 family, polypeptide A10
ZNF609	1.57	23060	ZNF609	zinc finger protein 609
RPRC1	1.56	55700	RPRC1	arginine/proline rich coiled-coil 1
E2F6	1.56	1876	E2F6	E2F transcription factor 6
EXTL3	1.56	2137	EXTL3	exostoses (multiple)-like 3
FAM104A	1.56	84923	FAM104A	family with sequence similarity 104, member A
JAG1	1.56	182	JAG1	jagged 1 (Alagille syndrome)
MEA1	1.56	4201	MEA1	male-enhanced antigen 1

POLR3H	1.56	171568	POLR3H	polymerase (RNA) III (DNA directed) polypeptide H (22.9kD)
SMARCA4	1.56	6597	SMARCA4	chromatin, subfamily a, member 4
TBL1XR1	1.56	79718	TBL1XR1	transducin (beta)-like 1X-linked receptor 1
TUBB2B	1.56	347733	TUBB2B	tubulin, beta 2B
C16ORF57	1.55	79650	C16orf57	chromosome 16 open reading frame 57
FLJ20186	1.55	54849	DEF8	differentially expressed in FDCP 8 homolog (mouse)
KIAA0427	1.55	9811	KIAA0427	KIAA0427
KIAA0690	1.55	23223	KIAA0690	KIAA0690
LACTB	1.55	114294	LACTB	lactamase, beta
PCSK1N	1.55	27344	PCSK1N	proprotein convertase subtilisin/kexin type 1 inhibitor
TMEM123	1.55	114908	TMEM123	transmembrane protein 123
ZNF219	1.55	51222	ZNF219	zinc finger protein 219
CIRH1A	1.54	84916	CIRH1A	cirrhosis, autosomal recessive 1A (cirhin)
HSPB8	1.54	26353	HSPB8	heat shock 22kDa protein 8
ITGB6	1.54	3694	ITGB6	integrin, beta 6
M6PR	1.54	4074	M6PR	mannose-6-phosphate receptor (cation dependent)
PRCP	1.54	5547	PRCP	prolylcarboxypeptidase (angiotensinase C)
KIAA1688	1.54	80728	ARHGAP39	Rho GTPase activating protein 39
TOLLIP	1.54	54472	TOLLIP	toll interacting protein
CREB3L2	1.53	64764	CREB3L2	cAMP responsive element binding protein 3-like 2
CPNE8	1.53	144402	CPNE8	copine VIII
DKC1	1.53	1736	DKC1	dyskeratosis congenita 1, dyskerin
GPC1	1.53	2817	GPC1	glypican 1
NME4	1.53	4833	NME4	non-metastatic cells 4, protein expressed in
RDH11	1.53	51109	RDH11	retinol dehydrogenase 11 (all-trans/9-cis/11-cis)
SLC38A1	1.53	81539	SLC38A1	solute carrier family 38, member 1
TRAM2	1.53	9697	TRAM2	translocation associated membrane protein 2
VAV2	1.53	7410	VAV2	vav 2 oncogene
ZDHHC16	1.53	84287	ZDHHC16	zinc finger, DHHC-type containing 16
AP2M1	1.52	1173	AP2M1	adaptor-related protein complex 2, mu 1 subunit
CALD1	1.52	800	CALD1	caldesmon 1
HMOX1	1.52	3162	HMOX1	heme oxygenase (decycling) 1
ILF3	1.52	3609	ILF3	interleukin enhancer binding factor 3, 90kDa
JARID2	1.52	3720	JARID2	jumonji, AT rich interactive domain 2
MAPRE3	1.52	22924	MAPRE3	microtubule-associated protein, RP/EB family, member 3
NFE2L3	1.52	9603	NFE2L3	nuclear factor (erythroid-derived 2)-like 3
PLEKHH3	1.52	79990	PLEKHH3	domain) member 3
SLC35E1	1.52	79939	SLC35E1	solute carrier family 35, member E1
TSPAN7	1.52	7102	TSPAN7	tetraspanin 7
TMEM113	1.52	80335	TMEM113	transmembrane protein 113
TMEM9	1.52	252839	TMEM9	transmembrane protein 9
UBN1	1.52	29855	UBN1	ubinuclein 1
ATG9A	1.51	79065	ATG9A	ATG9 autophagy related 9 homolog A (S. cerevisiae)
ATRN	1.51	8455	ATRN	attractin
BID	1.51	637	BID	BH3 interacting domain death agonist
ITGAV	1.51	3685	ITGAV	CD51)

NEO1	1.51	4756	NEO1	neogenin homolog 1 (chicken)
NFIL3	1.51	4783	NFIL3	nuclear factor, interleukin 3 regulated
CHFR	1.50	55743	CHFR	checkpoint with forkhead and ring finger domains
C22ORF16	1.50	400916	CHCHD10	coiled-coil-helix-coiled-coil-helix domain containing 10
FAM105B	1.50	90268	FAM105B	family with sequence similarity 105, member B
GPD1L	1.50	23171	GPD1L	glycerol-3-phosphate dehydrogenase 1-like
LOC339344	1.50	339344	MYPOP	Myb-related transcription factor, partner of profilin
NOL6	1.50	65083	NOL6	nucleolar protein family 6 (RNA-associated)
PYGB	1.50	5834	PYGB	phosphorylase, glycogen; brain
PTPN23	1.50	25930	PTPN23	protein tyrosine phosphatase, non-receptor type 23
RAI14	1.50	26064	RAI14	retinoic acid induced 14
SNRPN	1.50	6638	SNRPN	small nuclear ribonucleoprotein polypeptide N
SRP46	1.50	10929	SFRS2B	splicing factor, arginine/serine-rich 2B
SUPT4H1	1.50	6827	SUPT4H1	suppressor of Ty 4 homolog 1 (S. cerevisiae)
TNFAIP1	1.50	7126	TNFAIP1	tumor necrosis factor, alpha-induced protein 1 (endothelial)
ETS1	1.50	2113	ETS1	v-ets erythroblastosis virus E26 oncogene homolog 1 (avian)
ABHD4	1.49	63874	ABHD4	abhydrolase domain containing 4
AKR1B1	1.49	231	AKR1B1	aldo-keto reductase family 1, member B1 (aldose reductase)
CCNB1IP1	1.49	57820	CCNB1IP1	cyclin B1 interacting protein 1
LRPPRC	1.49	10128	LRPPRC	leucine-rich PPR-motif containing
LAPTM4B	1.49	55353	LAPTM4B	lysosomal protein transmembrane 4 beta
NOMO1	1.49	23420	NOMO1	NODAL modulator 1
SNX5	1.49	27131	SNX5	sorting nexin 5
SOX17	1.49	64321	SOX17	SRY (sex determining region Y)-box 17
ZDHHC23	1.49	254887	ZDHHC23	zinc finger, DHHC-type containing 23
11-Sep	1.49	55752	11-Sep	septin 11
IQWD1	1.49	55827	DCAF6	DDB1 and CUL4 associated factor 6
RIP	1.49	84268	RPAIN	RPA interacting protein
CLCN7	1.48	1186	CLCN7	chloride channel 7
CHPT1	1.48	56994	CHPT1	choline phosphotransferase 1
DNAJB2	1.48	3300	DNAJB2	DnaJ (Hsp40) homolog, subfamily B, member 2
GABARAPL	1.48	23710	GABARAPL1	GABA(A) receptor-associated protein like 1
HS1BP3	1.48	64342	HS1BP3	HCLS1 binding protein 3
HEXB	1.48	3074	HEXB	hexosaminidase B (beta polypeptide)
PPP1R14B	1.48	26472	PPP1R14B	protein phosphatase 1, regulatory (inhibitor) subunit 14B
PPP3R1	1.48	5534	PPP3R1	isoform
SERINC2	1.48	347735	SERINC2	serine incorporator 2
LYCAT	1.48	253558	LCLAT1	lysocardiolipin acyltransferase 1
CAV1	1.47	857	CAV1	caveolin 1, caveolae protein, 22kDa
CDCA4	1.47	55038	CDCA4	cell division cycle associated 4
EPAS1	1.47	2034	EPAS1	endothelial PAS domain protein 1
FOXA2	1.47	3170	FOXA2	forkhead box A2
NAGLU	1.47	4669	NAGLU	N-acetylglucosaminidase, alpha-
RAB15	1.47	376267	RAB15	RAB15, member RAS onocogene family
RAD54L2	1.47	23132	RAD54L2	RAD54-like 2 (S. cerevisiae)
SH3GL3	1.47	6457	SH3GL3	SH3-domain GRB2-like 3

SLC20A1	1.47	6574	SLC20A1	solute carrier family 20 (phosphate transporter), member 1
THBS1	1.47	7057	THBS1	thrombospondin 1
TTYH3	1.47	80727	TTYH3	tweety homolog 3 (Drosophila)
UBE2O	1.47	63893	UBE2O	ubiquitin-conjugating enzyme E2O
ELL	1.46	8178	ELL	elongation factor RNA polymerase II
GTF2F2	1.46	2963	GTF2F2	general transcription factor IIF, polypeptide 2, 30kDa
GNG10	1.46	2790	GNG10	guanine nucleotide binding protein (G protein), gamma 10
MTA1	1.46	9112	MTA1	metastasis associated 1
MFGE8	1.46	4240	MFGE8	milk fat globule-EGF factor 8 protein
ODC1	1.46	4953	ODC1	ornithine decarboxylase 1
PHF12	1.46	57649	PHF12	PHD finger protein 12
PODXL2	1.46	50512	PODXL2	podocalyxin-like 2
PSME4	1.46	23198	PSME4	proteasome (prosome, macropain) activator subunit 4
PUS7	1.46	54517	PUS7	pseudouridylate synthase 7 homolog (S. cerevisiae)
PDXP	1.46	57026	PDXP	pyridoxal (pyridoxine, vitamin B6) phosphatase
RANGAP1	1.46	5905	RANGAP1	Ran GTPase activating protein 1
SLC25A19	1.46	60386	SLC25A19	carrier), member 19
STOM	1.46	2040	STOM	stomatin
TAF2	1.46	6873	TAF2	associated factor, 150kDa
TNPO2	1.46	30000	TNPO2	transportin 2
XPR1	1.46	9213	XPR1	xenotropic and polytropic retrovirus receptor
TMEM32	1.46	93380	MMGT1	membrane magnesium transporter 1
AES	1.45	166	AES	amino-terminal enhancer of split
AZIN1	1.45	51582	AZIN1	antizyme inhibitor 1
CLCN3	1.45	1182	CLCN3	chloride channel 3
DAZAP1	1.45	26528	DAZAP1	DAZ associated protein 1
DDX55	1.45	57696	DDX55	DEAD (Asp-Glu-Ala-Asp) box polypeptide 55
DAP	1.45	1611	DAP	death-associated protein
DYNLL2	1.45	140735	DYNLL2	dynein, light chain, LC8-type 2
KIAA0319	1.45	9856	KIAA0319	KIAA0319
MBTPS1	1.45	8720	MBTPS1	membrane-bound transcription factor peptidase, site 1
NIPA2	1.45	81614	NIPA2	non imprinted in Prader-Willi/Angelman syndrome 2
WDR41	1.45	55255	WDR41	WD repeat domain 41
C16ORF28	1.45	64718	UNKL	unkempt homolog (Drosophila)-like
FAM62A	1.45	23344	ESYT1	extended synaptotagmin-like protein 1
HNRPR	1.45	10236	HNRNPR	heterogeneous nuclear ribonucleoprotein R
OACT5	1.45	10162	LPCAT3	lysophosphatidylcholine acyltransferase 3
ABL1	1.44	25	ABL1	c-abl oncogene 1, receptor tyrosine kinase
KLF6	1.44	1316	KLF6	Kruppel-like factor 6
PIM1	1.44	5292	PIM1	pim-1 oncogene
TRIM37	1.44	4591	TRIM37	tripartite motif-containing 37
ZNF134	1.44	7693	ZNF134	zinc finger protein 134
C20ORF121	1.44	79183	TTPAL	tocopherol (alpha) transfer protein-like
C20ORF35	1.44	55861	DBNDD2	dysbindin (dystrobrevin binding protein 1) domain containing 2
C6ORF166	1.44	55122	AKIRIN2	akirin 2
DKFZP761I2	1.44	83637	ZMIZ2	zinc finger, MIZ-type containing 2

ANKRD33	1.43	341405	ANKRD33	ankyrin repeat domain 33
CHSY1	1.43	22856	CHSY1	chondroitin sulfate synthase 1
DDX10	1.43	1662	DDX10	DEAD (Asp-Glu-Ala-Asp) box polypeptide 10
EIF4G3	1.43	8672	EIF4G3	eukaryotic translation initiation factor 4 gamma, 3
FKBP4	1.43	2288	FKBP4	FK506 binding protein 4, 59kDa
LEPROT	1.43	54741	LEPROT	leptin receptor overlapping transcript
MID1IP1	1.43	58526	MID1IP1	(zebrafish))
NDUFA6	1.43	4700	NDUFA6	NADH dehydrogenase (ubiquinone) 1 alpha subcomplex, 6, 14kDa
NECAP1	1.43	25977	NECAP1	NECAP endocytosis associated 1
NHP2L1	1.43	4809	NHP2L1	NHP2 non-histone chromosome protein 2-like 1 (S. cerevisiae)
PPP2R5E	1.43	5529	PPP2R5E	protein phosphatase 2, regulatory subunit B', epsilon isoform
SMOX	1.43	54498	SMOX	spermine oxidase
TNS3	1.43	64759	TNS3	tensin 3
UBQLN4	1.43	56893	UBQLN4	ubiquilin 4
FLJ13149	1.43	60493	FASTKD5	FAST kinase domains 5
PBEF1	1.43	10135	NAMPT	nicotinamide phosphoribosyltransferase
DHRS3	1.42	9249	DHRS3	dehydrogenase/reductase (SDR family) member 3
DDR1	1.42	780	DDR1	discoidin domain receptor tyrosine kinase 1
FST	1.42	10468	FST	follistatin
NELF	1.42	26012	NELF	nasal embryonic LHRH factor
NCBP2	1.42	22916	NCBP2	nuclear cap binding protein subunit 2, 20kDa
PLOD1	1.42	5351	PLOD1	procollagen-lysine 1, 2-oxoglutarate 5-dioxygenase 1
SYF2	1.42	25949	SYF2	SYF2 homolog, RNA splicing factor (S. cerevisiae)
TSPAN17	1.42	26262	TSPAN17	tetraspanin 17
TMEM41A	1.42	90407	TMEM41A	transmembrane protein 41A
TRIM5	1.42	85363	TRIM5	tripartite motif-containing 5
WFS1	1.42	7466	WFS1	Wolfram syndrome 1 (wolframin)
C8ORF1	1.42	734	OSGIN2	oxidative stress induced growth inhibitor family member 2
FLJ13912	1.42	64785	GINS3	GINS complex subunit 3 (Psf3 homolog)
FLJ14154	1.42	79903	NAT15	N-acetyltransferase 15 (GCN5-related, putative)
WDR39	1.42	9391	CIAO1	cytosolic iron-sulfur protein assembly 1
CLTC	1.41	1213	CLTC	clathrin, heavy chain (Hc)
H2AFY2	1.41	55506	H2AFY2	H2A histone family, member Y2
ITGA5	1.41	3678	ITGA5	integrin, alpha 5 (fibronectin receptor, alpha polypeptide)
JAK1	1.41	3716	JAK1	Janus kinase 1
MAOA	1.41	4128	MAOA	monoamine oxidase A
NFIB	1.41	4781	NFIB	nuclear factor I/B
PFKM	1.41	5213	PFKM	phosphofructokinase, muscle
PLEKHG3	1.41	26030	PLEKHG3	domain) member 3
POLR2D	1.41	5433	POLR2D	polymerase (RNA) II (DNA directed) polypeptide D
PHB2	1.41	11331	PHB2	prohibitin 2
SFXN1	1.41	94081	SFXN1	sideroflexin 1
TCEAL4	1.41	79921	TCEAL4	transcription elongation factor A (SII)-like 4
ZC3H8	1.41	84524	ZC3H8	zinc finger CCCH-type containing 8
ZNF419	1.41	79744	ZNF419	zinc finger protein 419
STX3A	1.41	6809	STX3	syntaxin 3

BOK	1.40	666	BOK	BCL2-related ovarian killer
COL4A1	1.40	1282	COL4A1	collagen, type IV, alpha 1
DNMT1	1.40	1786	DNMT1	DNA (cytosine-5-)-methyltransferase 1
ESRRA	1.40	2101	ESRRA	estrogen-related receptor alpha
TAF10	1.40	6881	TAF10	associated factor, 30kDa
UCRC	1.40	29796	UCRC	ubiquinol-cytochrome c reductase complex (7.2 kD)
USP39	1.40	10713	USP39	ubiquitin specific peptidase 39
RELB	1.40	5971	RELB	v-rel reticuloendotheliosis viral oncogene homolog B
ACSL5	-1.40	51703	ACSL5	acyl-CoA synthetase long-chain family member 5
ACOT11	-1.40	26027	ACOT11	acyl-CoA thioesterase 11
B2M	-1.40	567	B2M	beta-2-microglobulin
CLDN18	-1.40	51208	CLDN18	claudin 18
GCKR	-1.40	2646	GCKR	glucokinase (hexokinase 4) regulator
LMNA	-1.40	4000	LMNA	lamin A/C
MRPS31	-1.40	10240	MRPS31	mitochondrial ribosomal protein S31
PLAGL2	-1.40	5326	PLAGL2	pleiomorphic adenoma gene-like 2
RRAS2	-1.40	22800	RRAS2	related RAS viral (r-ras) oncogene homolog 2
RPS9	-1.40	6203	RPS9	ribosomal protein S9
ST14	-1.40	6768	ST14	suppression of tumorigenicity 14 (colon carcinoma)
C12ORF10	-1.40	60314	C12ORF10	chromosome 12 open reading frame 10
FAM82C	-1.40	55177	FAM82C	family with sequence similarity 82, member C
CARD11	-1.41	84433	CARD11	caspase recruitment domain family, member 11
C4BPB	-1.41	725	C4BPB	complement component 4 binding protein, beta
DDT	-1.41	1652	DDT	D-dopachrome tautomerase
H2BFS	-1.41	54145	H2BFS	H2B histone family, member S
IRF1	-1.41	3659	IRF1	interferon regulatory factor 1
PPIB	-1.41	5479	PPIB	peptidylprolyl isomerase B (cyclophilin B)
RPL18	-1.41	6141	RPL18	ribosomal protein L18
SLC9A3R1	-1.41	9368	SLC9A3R1	regulator 1
TOMM7	-1.41	54543	TOMM7	translocase of outer mitochondrial membrane 7 homolog (yeast)
C10ORF65	-1.41	112817	HOGA1	4-hydroxy-2-oxoglutarate aldolase 1
C1ORF116	-1.41	79098	C1orf116	chromosome 1 open reading frame 116
C20ORF54	-1.41	113278	C20orf54	chromosome 20 open reading frame 54
C9ORF16	-1.41	112817	DHDPSL	dihydrodipicolinate synthase-like, mitochondrial
FLJ11017	-1.41	55286	C4orf19	chromosome 4 open reading frame 19
SLITL2	-1.41	114990	VASN	vasorin
DECR1	-1.42	1666	DECR1	2,4-dienoyl CoA reductase 1, mitochondrial
DYNLT1	-1.42	6993	DYNLT1	dynein, light chain, Tctex-type 1
KRT13	-1.42	3860	KRT13	keratin 13
NDUFS4	-1.42	4724	NDUFS4	coenzyme Q reductase)
NDUFV1	-1.42	4723	NDUFV1	NADH dehydrogenase (ubiquinone) flavoprotein 1, 51kDa
RHOA	-1.42	387	RHOA	ras homolog gene family, member A
SAV1	-1.42	60485	SAV1	salvador homolog 1 (Drosophila)
COQ5	-1.43	84274	COQ5	coenzyme Q5 homolog, methyltransferase (S. cerevisiae)
GTF2E2	-1.43	2961	GTF2E2	general transcription factor IIE, polypeptide 2, beta 34kDa
QARS	-1.43	5859	QARS	glutaminyl-tRNA synthetase

MELK	-1.43	9833	MELK	maternal embryonic leucine zipper kinase
MRPS22	-1.43	56945	MRPS22	mitochondrial ribosomal protein S22
POLR2I	-1.43	5438	POLR2I	polymerase (RNA) II (DNA directed) polypeptide I, 14.5kDa
PPA2	-1.43	27068	PPA2	pyrophosphatase (inorganic) 2
XPC	-1.43	7508	XPC	xeroderma pigmentosum, complementation group C
C14ORF112	-1.43	51241	COX16	COX16 cytochrome c oxidase assembly homolog (S. cerevisiae)
PHACS	-1.43	84680	ACCS	1-aminocyclopropane-1-carboxylate synthase homolog
RPA3	-1.44	6119	RPA3	replication protein A3, 14kDa
SLC25A23	-1.44	79085	SLC25A23	member 23
AMY1C	-1.45	278	AMY1C	amylase, alpha 1C (salivary)
BCAT2	-1.45	587	BCAT2	branched chain aminotransferase 2, mitochondrial
CALB2	-1.45	794	CALB2	calbindin 2
CASP3	-1.45	836	CASP3	caspase 3, apoptosis-related cysteine peptidase
CDR2L	-1.45	30850	CDR2L	cerebellar degeneration-related protein 2-like
MYEOV	-1.45	26579	MYEOV	myelomas)
NMU	-1.45	10874	NMU	neuromedin U
ARHGAP27	-1.45	201176	ARHGAP27	Rho GTPase activating protein 27
SF3B5	-1.45	83443	SF3B5	splicing factor 3b, subunit 5, 10kDa
TXNIP	-1.45	10628	TXNIP	thioredoxin interacting protein
XRCC1	-1.45	7515	XRCC1	X-ray repair complementing defective repair in Chinese hamster cells 1
ZCCHC9	-1.45	84240	ZCCHC9	zinc finger, CCHC domain containing 9
AHNAK	-1.46	79026	AHNAK	AHNAK nucleoprotein
ANAPC11	-1.46	51529	ANAPC11	anaphase promoting complex subunit 11
COX7B2	-1.46	170712	COX7B2	cytochrome c oxidase subunit VIIb2
KRT15	-1.46	3866	KRT15	keratin 15
NACA	-1.46	4666	NACA	nascent polypeptide-associated complex alpha subunit
PKP2	-1.46	5318	PKP2	plakophilin 2
DNTTIP1	-1.47	116092	DNTTIP1	deoxynucleotidyltransferase, terminal, interacting protein 1
SOD1	-1.47	6647	SOD1	superoxide dismutase 1, soluble
C10ORF42	-1.47	90550	CCDC109A	coiled-coil domain containing 109A
MTHFS	-1.48	10588	MTHFS	cyclo-ligase)
CHP	-1.48	11261	CHP	calcium binding protein P22
CST1	-1.48	1469	CST1	cystatin SN
MRPL50	-1.48	54534	MRPL50	mitochondrial ribosomal protein L50
OSBPL7	-1.48	114881	OSBPL7	oxysterol binding protein-like 7
PMPCB	-1.48	9512	PMPCB	peptidase (mitochondrial processing) beta
PHYH	-1.48	5264	PHYH	phytanoyl-CoA 2-hydroxylase
S100A14	-1.48	57402	S100A14	S100 calcium binding protein A14
SLPI	-1.48	6590	SLPI	secretory leukocyte peptidase inhibitor
TLCD1	-1.48	116238	TLCD1	TLC domain containing 1
C22ORF13	-1.48	83606	C22orf13	chromosome 22 open reading frame 13
LOC389541	-1.48	389541	C7orf59	chromosome 7 open reading frame 59
MGC33212	-1.48	255758	TCTEX1D2	Tctex1 domain containing 2
AK1	-1.49	203	AK1	adenylate kinase 1
CBR4	-1.49	84869	CBR4	carbonyl reductase 4
DDAH1	-1.49	23576	DDAH1	dimethylarginine dimethylaminohydrolase 1

NP	-1.49	4860	NP	nucleoside phosphorylase
STIL	-1.49	6491	STIL	SCL/TAL1 interrupting locus
SF3B14	-1.49	51639	SF3B14	splicing factor 3B, 14 kDa subunit
ATP5H	-1.50	10476	ATP5H	ATP synthase, H+ transporting, mitochondrial F0 complex, subunit d
CSNK1D	-1.50	1453	CSNK1D	casein kinase 1, delta
CORO2A	-1.50	7464	CORO2A	coronin, actin binding protein, 2A
DUSP3	-1.50	1845	DUSP3	related)
GLE1L	-1.50	2733	GLE1L	GLE1 RNA export mediator-like (yeast)
KRT19	-1.50	3880	KRT19	keratin 19
MRPL22	-1.50	29093	MRPL22	mitochondrial ribosomal protein L22
MRPL40	-1.50	64976	MRPL40	mitochondrial ribosomal protein L40
PPL	-1.50	5493	PPL	periplakin
ST3GAL4	-1.50	6484	ST3GAL4	ST3 beta-galactoside alpha-2,3-sialyltransferase 4
SNCG	-1.50	6623	SNCG	synuclein, gamma (breast cancer-specific protein 1)
CITED2	-1.51	10370	CITED2	terminal domain, 2
C9ORF89	-1.51	84270	C9orf89	chromosome 9 open reading frame 89
CNTN1	-1.51	1272	CNTN1	contactin 1
IREB2	-1.51	3658	IREB2	iron-responsive element binding protein 2
MAPRE2	-1.51	10982	MAPRE2	microtubule-associated protein, RP/EB family, member 2
MTMR11	-1.51	10903	MTMR11	myotubularin related protein 11
RPS6	-1.51	6194	RPS6	ribosomal protein S6
S100P	-1.51	6286	S100P	S100 calcium binding protein P
SH3BGRL2	-1.51	83699	SH3BGRL2	SH3 domain binding glutamic acid-rich protein like 2
SLC22A18	-1.51	5002	SLC22A18	solute carrier family 22 (organic cation transporter), member 18
TSPAN3	-1.51	10099	TSPAN3	tetraspanin 3
CDC20	-1.52	991	CDC20	CDC20 cell division cycle 20 homolog (S. cerevisiae)
CCDC23	-1.52	374969	CCDC23	coiled-coil domain containing 23
MGC61571	-1.52	152100	CMC1	COX assembly mitochondrial protein homolog (S. cerevisiae)
GTF2H5	-1.52	404672	GTF2H5	general transcription factor IIH, polypeptide 5
KIAA0528	-1.52	9847	KIAA0528	KIAA0528
SDHD	-1.52	6392	SDHD	protein
C10ORF116	-1.53	10974	C10orf116	chromosome 10 open reading frame 116
F2RL1	-1.53	2150	F2RL1	coagulation factor II (thrombin) receptor-like 1
NANS	-1.53	54187	NANS	N-acetylneuraminic acid synthase (sialic acid synthase)
RND3	-1.53	390	RND3	Rho family GTPase 3
RPS13	-1.53	6207	RPS13	ribosomal protein S13
COMMD1	-1.54	150684	COMMD1	copper metabolism (Murr1) domain containing 1
DCAKD	-1.54	79877	DCAKD	dephospho-CoA kinase domain containing
LSM3	-1.54	27258	LSM3	LSM3 homolog, U6 small nuclear RNA associated (S. cerevisiae)
MRPL39	-1.54	54148	MRPL39	mitochondrial ribosomal protein L39
NDUFB7	-1.54	4713	NDUFB7	NADH dehydrogenase (ubiquinone) 1 beta subcomplex, 7, 18kDa
RPL39L	-1.54	116832	RPL39L	ribosomal protein L39-like
ALDH3A2	-1.55	224	ALDH3A2	aldehyde dehydrogenase 3 family, member A2
GANAB	-1.55	23193	GANAB	glucosidase, alpha; neutral AB
MYO18A	-1.55	399687	MYO18A	myosin XVIIIA
PDZK1IP1	-1.55	10158	PDZK1IP1	PDZK1 interacting protein 1

PET112L	-1.55	5188	PET112L	PET112-like (yeast)
PCK2	-1.55	5106	PCK2	phosphoenolpyruvate carboxykinase 2 (mitochondrial)
PLSCR1	-1.55	5359	PLSCR1	phospholipid scramblase 1
POLR3C	-1.55	10623	POLR3C	polymerase (RNA) III (DNA directed) polypeptide C (62kD)
RPS5	-1.55	6193	RPS5	ribosomal protein S5
BBS4	-1.56	585	BBS4	Bardet-Biedl syndrome 4
C21ORF25	-1.56	25966	C2CD2	C2 calcium-dependent domain containing 2
CHMP2A	-1.56	27243	CHMP2A	chromatin modifying protein 2A
CSTB	-1.56	1476	CSTB	cystatin B (stefin B)
C1ORF60	-1.56	65123	INTS3	integrator complex subunit 3
IL10RB	-1.56	3588	IL10RB	interleukin 10 receptor, beta
MGST3	-1.56	4259	MGST3	microsomal glutathione S-transferase 3
MRPS9	-1.56	64965	MRPS9	mitochondrial ribosomal protein S9
RPL4	-1.56	6124	RPL4	ribosomal protein L4
CTSB	-1.57	1508	CTSB	cathepsin B
FAM73A	-1.57	374986	FAM73A	family with sequence similarity 73, member A
PTGES	-1.57	9536	PTGES	prostaglandin E synthase
SPRR1A	-1.57	6698	SPRR1A	small proline-rich protein 1A
ZMYM6	-1.57	9204	ZMYM6	zinc finger, MYM-type 6
APBB3	-1.58	10307	APBB3	amyloid beta (A4) precursor protein-binding, family B, member 3
POLR3GL	-1.58	84265	POLR3GL	polymerase (RNA) III (DNA directed) polypeptide G (32kD) like
RAB26	-1.58	25837	RAB26	RAB26, member RAS oncogene family
THYN1	-1.58	29087	THYN1	thymocyte nuclear protein 1
CDC23	-1.59	8697	CDC23	CDC23 (cell division cycle 23, yeast, homolog)
GMDS	-1.59	2762	GMDS	GDP-mannose 4,6-dehydratase
MKRN2	-1.59	23609	MKRN2	makorin, ring finger protein, 2
PI3	-1.59	5266	PI3	peptidase inhibitor 3, skin-derived (SKALP)
FKSG30	-1.59	440915	POTEKP	POTE ankyrin domain family, member K, pseudogene
LOC201175	-1.59	201175	SH3D20	SH3 domain containing 20
C1ORF41	-1.60	51668	HSPB11	heat shock protein family B (small), member 11
MRCL3	-1.60	10627	MYL12A	myosin, light chain 12A, regulatory, non-sarcomeric
NMI	-1.60	9111	NMI	N-myc (and STAT) interactor
TSPAN1	-1.60	10103	TSPAN1	tetraspanin 1
CYB561	-1.61	1534	CYB561	cytochrome b-561
ELF3	-1.61	1999	ELF3	E74-like factor 3 (ets domain transcription factor, epithelial-specific)
TBC1D22A	-1.61	25771	TBC1D22A	TBC1 domain family, member 22A
WDR68	-1.61	10238	WDR68	WD repeat domain 68
C19ORF33	-1.62	64073	C19orf33	chromosome 19 open reading frame 33
EPS8L1	-1.62	54869	EPS8L1	EPS8-like 1
MDH1	-1.62	4190	MDH1	malate dehydrogenase 1, NAD (soluble)
NOLA2	-1.62	55651	NOLA2	nucleolar protein family A, member 2 (H/ACA small nucleolar RNPs)
PCNP	-1.62	57092	PCNP	PEST proteolytic signal containing nuclear protein
PPP3CB	-1.62	5532	PPP3CB	(calcineurin A beta)
PTD015	-1.63	28971	C11orf67	chromosome 11 open reading frame 67
NUDT8	-1.63	254552	NUDT8	nudix (nucleoside diphosphate linked moiety X)-type motif 8
OSTF1	-1.63	26578	OSTF1	osteoclast stimulating factor 1

S100A10	-1.63	6281	S100A10	S100 calcium binding protein A10
TP53INP2	-1.63	58476	TP53INP2	tumor protein p53 inducible nuclear protein 2
GALE	-1.63	2582	GALE	UDP-galactose-4-epimerase
M160	-1.64	283316	CD163L1	CD163 molecule-like 1
C12ORF62	-1.64	84987	C12orf62	chromosome 12 open reading frame 62
GTF2IRD2	-1.64	84163	GTF2IRD2	GTF2I repeat domain containing 2
SCNN1A	-1.64	6337	SCNN1A	sodium channel, nonvoltage-gated 1 alpha
HOXB5	-1.65	3215	HOXB5	homeobox B5
OBFC1	-1.65	79991	OBFC1	oligonucleotide/oligosaccharide-binding fold containing 1
PRSS3	-1.65	5646	PRSS3	protease, serine, 3 (mesotrypsin)
DHRS1	-1.66	115817	DHRS1	dehydrogenase/reductase (SDR family) member 1
IGFBP6	-1.66	3489	IGFBP6	insulin-like growth factor binding protein 6
NDUFB6	-1.66	4712	NDUFB6	NADH dehydrogenase (ubiquinone) 1 beta subcomplex, 6, 17kDa
TUFT1	-1.66	7286	TUFT1	tuftelin 1
LPP	-1.67	4026	LPP	LIM domain containing preferred translocation partner in lipoma
HIBCH	-1.68	26275	HIBCH	3-hydroxyisobutyryl-Coenzyme A hydrolase
FUT3	-1.68	2525	FUT3	blood group)
GLDC	-1.68	2731	GLDC	glycine dehydrogenase (decarboxylating)
SLC37A4	-1.68	2542	SLC37A4	solute carrier family 37 (glycerol-6-phosphate transporter), member 4
AVPI1	-1.69	60370	AVPI1	arginine vasopressin-induced 1
FDFT1	-1.69	2222	FDFT1	farnesyl-diphosphate farnesyltransferase 1
RALB	-1.69	5899	RALB	binding protein)
AUH	-1.70	549	AUH	AU RNA binding protein/enoyl-Coenzyme A hydratase
MTX2	-1.71	10651	MTX2	metaxin 2
PDCD6IP	-1.71	10015	PDCD6IP	programmed cell death 6 interacting protein
FLJ10986	-1.72	55277	FGGY	FGGY carbohydrate kinase domain containing
GLYCTK	-1.73	132158	GLYCTK	glycerate kinase
MAPK3	-1.73	5595	MAPK3	mitogen-activated protein kinase 3
ASS	-1.76	445	ASS1	argininosuccinate synthase 1
ALG5	-1.76	29880	ALG5	phosphate beta-glucosyltransferase)
CIB1	-1.76	10519	CIB1	calcium and integrin binding 1 (calmyrin)
LGALS3	-1.76	3958	LGALS3	lectin, galactoside-binding, soluble, 3 (galectin 3)
MRPS15	-1.76	64960	MRPS15	mitochondrial ribosomal protein S15
FLJ22662	-1.76	79887	PLBD1	phospholipase B domain containing 1
GALM	-1.77	130589	GALM	galactose mutarotase (aldose 1-epimerase)
TJP3	-1.77	27134	TJP3	tight junction protein 3 (zona occludens 3)
ZNF503	-1.77	84858	ZNF503	zinc finger protein 503
ACTB	-1.79	60	ACTB	actin, beta
CDC42EP5	-1.79	148170	CDC42EP5	CDC42 effector protein (Rho GTPase binding) 5
KRT18	-1.79	3875	KRT18	keratin 18
SCD	-1.79	6319	SCD	stearoyl-CoA desaturase (delta-9-desaturase)
C9ORF46	-1.80	55848	C9orf46	chromosome 9 open reading frame 46
ACTG1	-1.81	71	ACTG1	actin, gamma 1
IVNS1ABP	-1.81	10625	IVNS1ABP	influenza virus NS1A binding protein
KCNS1	-1.81	3787	KCNS1	member 1
CAPN5	-1.82	726	CAPN5	calpain 5

C15ORF48	-1.82	84419	C15orf48	chromosome 15 open reading frame 48
MGC20255	-1.82	90324	CCDC97	coiled-coil domain containing 97
EIF4A2	-1.83	1974	EIF4A2	eukaryotic translation initiation factor 4A, isoform 2
CYP4F12	-1.84	66002	CYP4F12	cytochrome P450, family 4, subfamily F, polypeptide 12
STAMBP	-1.84	10617	STAMBP	STAM binding protein
TNFRSF14	-1.84	8764	TNFRSF14	entry mediator)
ALDOA	-1.86	226	ALDOA	aldolase A, fructose-bisphosphate
GPR92	-1.86	57121	GPR92	G protein-coupled receptor 92
SCEL	-1.86	8796	SCEL	sciellin
SEMA3B	-1.86	7869	SEMA3B	secreted, (semaphorin) 3B
TSC22D3	-1.86	1831	TSC22D3	TSC22 domain family, member 3
ANXA3	-1.88	306	ANXA3	annexin A3
FGF19	-1.88	9965	FGF19	fibroblast growth factor 19
MVP	-1.88	9961	MVP	major vault protein
CFB	-1.91	629	CFB	complement factor B
ATAD4	-1.92	79170	ATAD4	ATPase family, AAA domain containing 4
SLC16A5	-1.92	9121	SLC16A5	solute carrier family 16, member 5 (monocarboxylic acid transporter 6)
TMEM16A	-1.92	55107	TMEM16A	transmembrane protein 16A
FXYD3	-1.94	5349	FXYD3	FXYD domain containing ion transport regulator 3
TNFSF5IP1	-1.95	56984	TNFSF5IP1	tumor necrosis factor superfamily, member 5-induced protein 1
UNC93B1	-1.97	81622	UNC93B1	unc-93 homolog B1 (C. elegans)
MMP28	-1.99	79148	MMP28	matrix metallopeptidase 28
TPK1	-2.02	27010	TPK1	thiamin pyrophosphokinase 1
LGR4	-2.06	55366	LGR4	leucine-rich repeat-containing G protein-coupled receptor 4
F3	-2.08	2152	F3	coagulation factor III (thromboplastin, tissue factor)
MUC20	-2.10	200958	MUC20	mucin 20, cell surface associated
FLJ20625	-2.11	55004	C11orf59	chromosome 11 open reading frame 59
CKB	-2.11	1152	CKB	creatine kinase, brain
GLS	-2.13	2744	GLS	glutaminase
PQLC3	-2.15	130814	PQLC3	PQ loop repeat containing 3
ALDH3A1	-2.16	218	ALDH3A1	aldehyde dehydrogenase 3 family, memberA1
TRIM31	-2.16	11074	TRIM31	tripartite motif-containing 31
RNASET2	-2.25	8635	RNASET2	ribonuclease T2
SDCBP2	-2.25	27111	SDCBP2	syndecan binding protein (syntenin) 2
CD68	-2.26	968	CD68	CD68 molecule
KRT8	-2.26	3856	KRT8	keratin 8
SERPINB1	-2.35	1992	SERPINB1	serpin peptidase inhibitor, clade B (ovalbumin), member 1
C	-2.38	8338	HIST2H2AC	histone cluster 2, H2ac
A	-2.41	8337	HIST2H2AA3	histone cluster 2, H2aa3
ARHGDIB	-2.46	397	ARHGDIB	Rho GDP dissociation inhibitor (GDI) beta
DPM3	-2.54	54344	DPM3	dolichyl-phosphate mannosyltransferase polypeptide 3
CEACAM1	-2.56	634	CEACAM1	glycoprotein)
CEACAM6	-2.56	4680	CEACAM6	specific cross reacting antigen)
SCGB1A1	-2.69	7356	SCGB1A1	secretoglobin, family 1A, member 1 (uteroglobin)
MUC13	-2.71	56667	MUC13	mucin 13, cell surface associated
CTGF	-2.77	1490	CTGF	connective tissue growth factor

MUC1	-3.19	4582	MUC1	mucin 1, cell surface associated
PSG9	-3.30	5678	PSG9	pregnancy specific beta-1-glycoprotein 9
TINP1	-3.45	10412	NSA2	NSA2 ribosome biogenesis homolog (S. cerevisiae)
PSG11	-3.46	5680	PSG11	pregnancy specific beta-1-glycoprotein 11
CEACAM5	-3.97	1048	CEACAM5	carcinoembryonic antigen-related cell adhesion molecule 5
AGT	-4.58	183	AGT	angiotensinogen (serpin peptidase inhibitor, clade A, member 8)
SPRR3	-4.66	6707	SPRR3	small proline-rich protein 3
CLDN7	-6.88	1366	CLDN7	claudin 7
