# Supplementary material for: Claudin-7 Is Frequently Overexpressed in Ovarian Cancer and Promotes Invasion
Source: PLoS One. 2011 Jul 15;6(7):e22119. doi: 10.1371/journal.pone.0022119 (PMC3137611; doi:10.1371/journal.pone.0022119)
Supplement: Table S3 — Ingenuity Pathway Analysis results for genes altered following CLDN7 knockdown. A. Top networks. B. Top Canonical pathways. C. Top Biological functions. (PDF) [file pone.0022119.s003.pdf]

Supp. Table 3(A-C) "Global functional analysis" conducted using Ingenuity Pathway Analysis

Supp. Table 3A: Top Networks

OVCAR2	
**Associated network functions**	**Score**
Amino Acid Metabolism, Post-Translational Modification, Small Molecule Biochemistry	51
Hematological System Development and Function, Immune and Lymphatic System development and Function, Tissue Morphology	41
Cellular Development, Cancer, gene Expression	37
Cell Signaling, Cell death, Cellular Growth and Proliferation	34
Lipid metabolism, Small Molecule Biochemistry, Molecular Transport	34

OVCA420	
**Associated network functions**	**Score**
Organismal Injusry and abnormalitis, cellular movement, Hematological System development and Function	49
Molecular transport, RNA trafficking, Cancer	46
Cancer, Cell death, cell cycle	37
Cancer, Cell Cycle, Gene Expression	33
Lipid metabolism, Molecular transport, Small Molecule Biochemistry	30

Supp Table 3B: Top Canonical Pathways

OVCAR2		
**Name**	**p-value**	**Ratio**
IGF-1 Signaling	3.20E-04	12/92 (0.13)
Glucocoticoid receptor Signaling	4.64E-04	24/275 (0.087)
Ceramide Signaling	1.13E-03	10/79 (0.127)
N-Glycan Biosynthesis	1.21E-03	8/87 (0.092)
Integrin Signaling	1.76E-03	18/192 (0.094)

OVCA420		
**Name**	**p-value**	**Ratio**
Integrin Signaling	6.29E-07	20/192 (0.104)
NRF2-mediated Oxidative Stress Response	4.30E-04	14/180 (0.078)
T Cell Receptor Signaling	1.37E-03	9/102 (0.088)
GM_CSF Signaling	1.52E-03	7/62 (0.113)
Actin Cytoskeleton signaling	1.73E-03	14/218 (0.064)

Supp. Table 3C: Top Biological Functions

OVCAR2			OVCA420		
**Diseases and disorders**					
**Name**	**p-value**	**#Molecules**	**Name**	**p-value**	**#Molecules**
Cancer	1.06E-06-8.96E-03	209	Cancer	9.65E-06-1.45E-02	164
Reproductive System Disorders	2.71E-05-6.89E-03	70	Gastrointestinal Disease	3.88-05-1.16E-02	65
Gastrointestinal Disease	6.59E-05-4.39E-03	85	Reproductive system disease	4.54-05-1.45E-02	49
Endocrine System Disorders	8.94E-05-8.96E-03	39	Connective tissue disorders	1.16E-04-1.33E-02	31
Metabolic Disease	8.94E-05-5.61E-03	17	Organismal Injury and Abnormalities	1.25E-04-1.43E-02	26

Molecular and Cellular Functions					
**Name**	**p-value**	**#Molecules**	**Name**	**p-value**	**#Molecules**
Cellular Growth and Proliferation	8.52E-08-8.77E-03	188	Cellular growth and Proliferation	1.18E-08-1.39E-02	125
Protein Synthesis	4.42E-06-6.42E-03	45	Cell Morphology	3.43E-07-1.43E-02	59
Cell Death	7.76E-06-8.96E-03	164	Cellular Development	5.11E-07-1.39E-02	66
Cell Cycle	1.70E-05-8.74E-03	86	Cell death	7.12E-07-1.45E-02	107
Cellular Development	2.17E-05-8.99E-03	133	Cellular Movement	9.65E-07-1.45E-02	78

Physiological System development and function					
**Name**	**p-value**	**#Molecules**	**Name**	**p-value**	**#Molecules**
Hematological System development and Function	9.14E-05-7.94E-03	96	Connective tissue development and Function	6.88E-06-1.38E-02	39
Immune Response	9.14E-05-7.59E-03	97	Tissue Development	6.88E-06-1.34E-02	39
Connective Tissue development and Function	1.06E-05-5.61E-03	54	Hematological System development and Function	2.89E-05-1.43E-02	45
Immune and Lymphatic System Development and Function	1.46E-04-7.33E-03	80	Immune Response	3.45E-05-1.43E-02	32
Cardiovascular System Development and Function	2.42E-04-8.43E-03	46	Immune and Lymphatic System development and Function	6.93E-05-1.34E-02	34

The list includes the annotation of the differentially expressed genes upon knockdown. The likelihood that the association between a set of differentially expressed genes and a given function was due to chance was calculated using the right-tailed Fisher's exact test. The biological functions or processes annotated to affected genes are listed according to their
