# Supplementary material for: Claudin-7 Is Frequently Overexpressed in Ovarian Cancer and Promotes Invasion
Source: PLoS One. 2011 Jul 15;6(7):e22119. doi: 10.1371/journal.pone.0022119 (PMC3137611; doi:10.1371/journal.pone.0022119)
Supplement: Table S4 — GO analysis of significantly altered genes in OVCAR2 and OVCA420 following CLDN7 knockdown. (PDF) [file pone.0022119.s004.pdf]

Supp. Table 4A: GO analysis of significantly altered genes in OVCAR2 and OVCA420 after knockdown of Claudin-7 using siRNA

OVCAR2

Symbol	Gene ID	Gene Name	Fold change
Genes in structural molecule activity			
MRPS30	10884	mitochondrial ribosomal protein S30	-1.6
RPS13	6207	ribosomal protein S13	-1.53
RPL36AL	6166	ribosomal protein L36a-like	-1.68
MRPL9	65005	mitochondrial ribosomal protein L9	-1.62
DNM3	26052	dynamin 3	-1.72
CLDN15	24146	claudin 15	1.58
LAMA3	3909	laminin, alpha 3	-1.99
UPK1B	7348	uroplakin 1B	-1.74
KRT19	3880	keratin 19	-1.62
RPS7	6201	ribosomal protein S7	-2.54
MRPL21	219927	mitochondrial ribosomal protein L21	-1.71
ARPC5	10092	actin related protein 2/3 complex, subunit 5, 16kDa	-1.52
RPL24	6152	ribosomal protein L24	-1.88
RPL14	9045	ribosomal protein L14	-1.58
CLDN7	1366	claudin 7	-6.97
RPL7L1	285855	ribosomal protein L7-like 1	-1.5
MRPL1	65008	mitochondrial ribosomal protein L1	-1.52
RSL1D1	26156	ribosomal L1 domain containing 1	-2.3
MRPS18C	51023	mitochondrial ribosomal protein S18C	-1.59
MRPL36	64979	mitochondrial ribosomal protein L36	2.2
LLGL1	3996	lethal giant larvae homolog 1 (Drosophila)	1.7
RPS21	6227	ribosomal protein S21	-1.52
RPL39L	116832	ribosomal protein L39-like	-1.56
RPS15	6209	ribosomal protein S15	-1.84
COL4A2	1284	collagen, type IV, alpha 2	-1.91
MRPS15	64960	mitochondrial ribosomal protein S15	-2.73
ADD3	120	adducin 3 (gamma)	1.73
MRPL33	9553	mitochondrial ribosomal protein L33	-1.78
MRPL20	55052	mitochondrial ribosomal protein L20	-1.5
CTHRC1	115908	collagen triple helix repeat containing 1	-1.92
CLTC	1213	clathrin, heavy chain (Hc)	1.87
ACTG1	71	actin, gamma 1	-1.66
COL18A1	80781	collagen, type XVIII, alpha 1	2.04
PLEC1	5339	plectin 1, intermediate filament binding protein 500kDa	-1.77
COL4A5	1287	collagen, type IV, alpha 5 (Alport syndrome)	1.71
ANXA1	301	annexin A1	-1.84
LAMB2	3913	laminin, beta 2 (laminin S)	1.74
MRPL22	29093	mitochondrial ribosomal protein L22	-1.81
RPL34	6164	ribosomal protein L34	-2.3
TINAGL1	64129	tubulointerstitial nephritis antigen-like 1	-2.94
FLG	2312	filaggrin	-2.26
MRPS17	51373	mitochondrial ribosomal protein S17	-2.36

RPL29	6159	ribosomal protein L29	-2.4
FBLN1	2192	fibulin 1	1.84
RPL4	6124	ribosomal protein L4	-1.5
CAV1	857	caveolin 1, caveolae protein, 22kDa	-1.6
Genes in signal transducer activity			
FOLR3	2352	folate receptor 3 (gamma)	-2.65
PGRMC1	10857	progesterone receptor membrane component 1	1.57
SEMA4D	10507	sema domain, immunoglobulin domain (Ig), transmembrane domain (TM) and short cytoplasmic domain, (semaphorin) 4D	1.58
DDR1	780	discoidin domain receptor family, member 1	1.79
TSPAN6	7105	tetraspanin 6	1.6
PRKCD	5580	protein kinase C, delta	1.52
TOMM22	56993	translocase of outer mitochondrial membrane 22 homolog (yeast)	-1.59
LPHN1	22859	latrophilin 1	1.76
RAF1	5894	v-raf-1 murine leukemia viral oncogene homolog 1	2.27
DEDD2	162989	death effector domain containing 2	1.54
CXCR4	7852	chemokine (C-X-C motif) receptor 4	-1.77
LRP10	26020	low density lipoprotein receptor-related protein 10	2.28
PLXNB1	5364	plexin B1	1.82
IGF2R	3482	insulin-like growth factor 2 receptor	1.88
DKK1	22943	dickkopf homolog 1 (Xenopus laevis)	2.09
PRKRA	8575	protein kinase, interferon-inducible double stranded RNA dependent activator	-1.58
NCK2	8440	NCK adaptor protein 2	1.54
LBR	3930	lamin B receptor	1.56
SRPR	6734	signal recognition particle receptor ('docking protein')	-1.85
MAP3K7	6885	mitogen-activated protein kinase kinase kinase 7	-1.53
CDK2AP1	8099	CDK2-associated protein 1	2.02
IQWD1	55827	IQ motif and WD repeats 1	1.75
FGFR3	2261	fibroblast growth factor receptor 3 (achondroplasia, thanatophoric dwarfism)	2.39
PAQR8	85315	progestin and adipoQ receptor family member VIII	2.02
LGALS3BP	3959	lectin, galactoside-binding, soluble, 3 binding protein	1.52
IL4R	3566	interleukin 4 receptor	1.66
MET	4233	met proto-oncogene (hepatocyte growth factor receptor)	1.8
NR1H2	7376	nuclear receptor subfamily 1, group H, member 2	1.91
STAT5B	6777	signal transducer and activator of transcription 5B	1.6
TNFSF10	8743	tumor necrosis factor (ligand) superfamily, member 10	2.04
FZD2	2535	frizzled homolog 2 (Drosophila)	2.64
IFNGR1	3459	interferon gamma receptor 1	1.67
MAPK6	5597	mitogen-activated protein kinase 6	1.87
NCOA7	135112	nuclear receptor coactivator 7	2.55
TNFRSF14	8764	tumor necrosis factor receptor superfamily, member 14 (herpesvirus entry mediator)	1.65
ITPR3	3710	inositol 1,4,5-triphosphate receptor, type 3	2.48
CXADR	1525	coxsackie virus and adenovirus receptor	2.27
NR2F6	2063	nuclear receptor subfamily 2, group F, member 6	1.56
SORT1	6272	sortilin 1	1.62
SCARB2	950	scavenger receptor class B, member 2	2.14
IFITM1	8519	interferon induced transmembrane protein 1 (9-27)	1.76
DGCR2	9993	DiGeorge syndrome critical region gene 2	1.52

PLXNA1	5361	plexin A1	1.66
S100A9	6280	S100 calcium binding protein A9	1.64
CCBP2	1238	chemokine binding protein 2	1.66
FCGRT	2217	Fc fragment of IgG, receptor, transporter, alpha	1.84
TNFRSF12A	51330	tumor necrosis factor receptor superfamily, member 12A	-1.82
PVRL2	5819	poliovirus receptor-related 2 (herpesvirus entry mediator B)	1.82
PRKCZ	5590	protein kinase C, zeta	2.71
LMBR1L	55716	limb region 1 homolog (mouse)-like	1.83
IRAK2	3656	interleukin-1 receptor-associated kinase 2	1.53
GNAI2	2771	guanine nucleotide binding protein (G protein), alpha inhibiting activity polypeptide 2	1.9
Genes in transcription regulator activity			
CREG1	8804	cellular repressor of E1A-stimulated genes 1	1.98
TSC22D3	1831	TSC22 domain family, member 3	1.58
CEBPD	1052	CCAAT/enhancer binding protein (C/EBP), delta	1.92
E2F4	1874	E2F transcription factor 4, p107/p130-binding	-1.66
CEBPB	1051	CCAAT/enhancer binding protein (C/EBP), beta	1.95
FOXC1	2296	forkhead box C1	1.71
IFI16	3428	interferon, gamma-inducible protein 16	2.21
FOXQ1	94234	forkhead box Q1	2.55
GTF2E2	2961	general transcription factor IIE, polypeptide 2, beta 34kDa	-1.88
LASS2	29956	LAG1 homolog, ceramide synthase 2 (S. cerevisiae)	-1.57
ID2	3398	inhibitor of DNA binding 2, dominant negative helix-loop-helix	1.68
DNM2	1785	dynamin 2	1.7
HOXB4	3214	homeobox B4	1.59
BOLA3	388962	bolA homolog 3 (E. coli)	-1.61
TRFP	9477	Trf (TATA binding protein-related factor)-proximal homolog (Drosophila)	-1.5
ID1	3397	protein	1.86
ZNF447	65982	zinc finger protein 447	1.73
FOSL1	8061	FOS-like antigen 1	-2.57
RPL7L1	285855	ribosomal protein L7-like 1	-1.5
MSRB2	22921	methionine sulfoxide reductase B2	1.64
FOXJ3	22887	forkhead box J3	1.64
MTA3	57504	metastasis associated 1 family, member 3	1.58
ELL	8178	elongation factor RNA polymerase II	1.59
IRF8	3394	interferon regulatory factor 8	-1.87
TEAD2	8463	TEA domain family member 2	3.73
CREB3L2	64764	cAMP responsive element binding protein 3-like 2	3.19
ILF3	3609	interleukin enhancer binding factor 3, 90kDa	1.59
SREBF1	6720	sterol regulatory element binding transcription factor 1	1.64
TRIP4	9325	thyroid hormone receptor interactor 4	-1.51
NR1H2	7376	nuclear receptor subfamily 1, group H, member 2	1.91
STAT5B	6777	signal transducer and activator of transcription 5B	1.6
MXD4	10608	MAX dimerization protein 4	1.99
TGIF2	60436	TGFB-induced factor 2 (TALE family homeobox)	1.7
NFIL3	4783	nuclear factor, interleukin 3 regulated	1.84
NR2F6	2063	nuclear receptor subfamily 2, group F, member 6	1.56
FHL2	2274	four and a half LIM domains 2	-1.51
RAN	5901	RAN, member RAS oncogene family	1.75
PHB	5245	prohibitin	-1.56

APEX1	328	APEX nuclease (multifunctional DNA repair enzyme) 1	-1.6
ARID1A	8289	AT rich interactive domain 1A (SWI- like)	1.91
HOXA5	3202	homeobox A5	2.25
DSCR1	1827	Down syndrome critical region gene 1	2.15
ZNF219	51222	zinc finger protein 219	1.68
SMARCA4	6597	SWI/SNF related, matrix associated, actin dependent regulator of chromatin, subfamily a, member 4	1.81
ZFP36L1	677	zinc finger protein 36, C3H type-like 1	1.6
PCGF2	7703	polycomb group ring finger 2	1.72
TAF10	6881	TAF10 RNA polymerase II, TATA box binding protein (TBP)-associated factor, 30kDa	1.54
E2F6	1876	E2F transcription factor 6	1.55
PA2G4	5036	proliferation-associated 2G4, 38kDa	-1.82
TFAP2A	7020	transcription factor AP-2 alpha (activating enhancer binding protein 2 alpha)	1.92
HOXA2	3199	homeobox A2	1.82
SUPT4H1	6827	suppressor of Ty 4 homolog 1 (S. cerevisiae)	1.7
IRF1	3659	interferon regulatory factor 1	1.51
TSC22D4	81628	TSC22 domain family, member 4	1.56
PIAS3	10401	protein inhibitor of activated STAT, 3	1.61
EDF1	8721	endothelial differentiation-related factor 1	-1.55
Genes in translation regulator activity			
EIF2A	83939	eukaryotic translation initiation factor 2A, 65kDa	-1.61
EIF3S7	8664	eukaryotic translation initiation factor 3, subunit 7 zeta, 66/67kDa	-1.82
EIF4A2	1974	eukaryotic translation initiation factor 4A, isoform 2	-1.53
MRPL9	65005	mitochondrial ribosomal protein L9	-1.62
ITGB4BP	3692	integrin beta 4 binding protein	-1.75
GFM1	85476	G elongation factor, mitochondrial 1	-1.54
EEF1A2	1917	eukaryotic translation elongation factor 1 alpha 2	1.52
EIF2B1	1967	eukaryotic translation initiation factor 2B, subunit 1 alpha, 26kDa	-1.97
EIF2B2	8892	eukaryotic translation initiation factor 2B, subunit 2 beta, 39kDa	-1.68
MTIF2	4528	mitochondrial translational initiation factor 2	-1.6
ICT1	3396	immature colon carcinoma transcript 1	-1.57
Genes in transporter activity			
UQCRC2	7385	ubiquinol-cytochrome c reductase core protein II	-1.88
KPNA3	3839	karyopherin alpha 3 (importin alpha 4)	-1.55
SEC61A1	29927	Sec61 alpha 1 subunit (S. cerevisiae)	1.74
SLC5A6	8884	solute carrier family 5 (sodium-dependent vitamin transporter), member 6	-1.65
ATP5I	521	ATP synthase, H+ transporting, mitochondrial F0 complex, subunit E	-1.54
TRPC6	7225	transient receptor potential cation channel, subfamily C, member 6	2.35
PLSCR1	5359	phospholipid scramblase 1	-1.64
COX6B1	1340	cytochrome c oxidase subunit Vib polypeptide 1 (ubiquitous)	-1.51
KCNS1	3787	member 1	-1.79
ATP5H	10476	ATP synthase, H+ transporting, mitochondrial F0 complex, subunit d	-1.54
SFXN1	94081	sideroflexin 1	1.6
TOMM7	54543	translocase of outer mitochondrial membrane 7 homolog (yeast)	-1.52
SYTL2	54843	synaptotagmin-like 2	-2.81
IGF2R	3482	insulin-like growth factor 2 receptor	1.88
SLC17A5	26503	solute carrier family 17 (anion/sugar transporter), member 5	1.52

SLC35B1	10237	solute carrier family 35, member B1	2.73
ATP6AP1	537	ATPase, H+ transporting, lysosomal accessory protein 1	2.12
SLC11A2	4891	solute carrier family 11 (proton-coupled divalent metal ion transporters), member 2	1.63
AP3D1	8943	adaptor-related protein complex 3, delta 1 subunit	1.6
ATG9A	79065	ATG9 autophagy related 9 homolog A (S. cerevisiae)	1.72
SLC25A22	79751	solute carrier family 25 (mitochondrial carrier: glutamate), member 22	1.85
TSNAX	7257	translin-associated factor X	-1.56
SLC39A6	25800	solute carrier family 39 (zinc transporter), member 6	2.1
ATP1B3	483	ATPase, Na+/K+ transporting, beta 3 polypeptide	1.63
COPB2	9276	coatomer protein complex, subunit beta 2 (beta prime)	2.03
VDAC2	7417	voltage-dependent anion channel 2	-1.73
ITPR3	3710	inositol 1,4,5-triphosphate receptor, type 3	2.48
TM9SF4	9777	transmembrane 9 superfamily protein member 4	1.91
SORT1	6272	sortilin 1	1.62
ATP5O	539	ATP synthase, H+ transporting, mitochondrial F1 complex, O subunit	-1.5
ATP5G1	516	ATP synthase, H+ transporting, mitochondrial F0 complex, subunit C1 (subunit 9)	-2.03
SLC37A1	54020	solute carrier family 37 (glycerol-3-phosphate transporter), member 1	2.11
TMCO3	55002	transmembrane and coiled-coil domains 3	1.66
SLCO2A1	6578	solute carrier organic anion transporter family, member 2A1	2.85
AP2M1	1173	adaptor-related protein complex 2, mu 1 subunit	1.62
SLC29A4	222962	solute carrier family 29 (nucleoside transporters), member 4	2.12
TOMM20	9804	translocase of outer mitochondrial membrane 20 homolog (yeast)	-1.93
APOE	348	apolipoprotein E	4.02
ARFGAP3	26286	ADP-ribosylation factor GTPase activating protein 3	1.8

OVCA420

Symbol	Gene ID	Gene Name	Fold change
		Genes in structural molecule activity	
MRPL36	64979	mitochondrial ribosomal protein L36	3.06
LAMB1	3912	laminin, beta 1	1.91
TUBA1	7277	tubulin, alpha 1	1.72
KRT20	54474	keratin 20	1.71
COL4A5	1287	collagen, type IV, alpha 5 (Alport syndrome)	1.68
COL17A1	1308	collagen, type XVII, alpha 1	1.61
TUBB	203068	tubulin, beta	1.6
MSN	4478	moesin	1.6
CLDN15	24146	claudin 15	1.57
JAG1	182	jagged 1 (Alagille syndrome)	1.56
MRPL22	29093	mitochondrial ribosomal protein L22	-1.5
KRT19	3880	keratin 19	-1.5
PPL	5493	periplakin	-1.5
RPS6	6194	ribosomal protein S6	-1.51
RPS13	6207	ribosomal protein S13	-1.53
RPL39L	116832	ribosomal protein L39-like	-1.54
RPS5	6193	ribosomal protein S5	-1.55

MRPS9	64965	mitochondrial ribosomal protein S9	-1.56
RPL4	6124	ribosomal protein L4	-1.56
SPRR1A	6698	small proline-rich protein 1A	-1.57
TUFT1	7286	tuftelin 1	-1.66
MRPS15	64960	mitochondrial ribosomal protein S15	-1.76
KRT18	3875	keratin 18	-1.79
ACTB	60	actin, beta	-1.79
ACTG1	71	actin, gamma 1	-1.81
KRT8	3856	keratin 8	-2.26
SPRR3	6707	small proline-rich protein 3	-4.66
CLDN7	1366	claudin 7	-6.88
Genes in signal transducer activity			
SH3BP4	23677	SH3-domain binding protein 4	1.58
DKK1	22943	dickkopf homolog 1 (Xenopus laevis)	2.87
FGFRL1	53834	fibroblast growth factor receptor-like 1	1.65
ATRN	8455	attractin	1.51
SCARB1	949	scavenger receptor class B, member 1	2.34
TMEM123	114908	transmembrane protein 123	1.55
ITGAV	3685	integrin, alpha V (vitronectin receptor, alpha polypeptide, antigen	1.51
IGF2R	3482	insulin-like growth factor 2 receptor	1.65
TOLLIP	54472	toll interacting protein	1.54
SEMA3B	7869	sema domain, immunoglobulin domain (Ig), short basic domain, secreted, (semaphorin) 3B	-1.86
MAPK6	5597	mitogen-activated protein kinase 6	2.15
SRPRB	58477	signal recognition particle receptor, B subunit	1.63
M6PR	4074	mannose-6-phosphate receptor (cation dependent)	1.54
F3	2152	coagulation factor III (thromboplastin, tissue factor)	-2.08
MAPK3	5595	mitogen-activated protein kinase 3	-1.73
CDK2AP1	8099	CDK2-associated protein 1	1.77
GJA1	2697	gap junction protein, alpha 1, 43kDa (connexin 43)	1.64
PDCD6IP	10015	programmed cell death 6 interacting protein	-1.71
WNT7A	7476	wingless-type MMTV integration site family, member 7A	1.72
CRY1	1407	cryptochrome 1 (photolyase-like)	1.73
NEO1	4756	neogenin homolog 1 (chicken)	1.51
IL10RB	3588	interleukin 10 receptor, beta	-1.56
CXADR	1525	coxsackie virus and adenovirus receptor	2.15
MET	4233	met proto-oncogene (hepatocyte growth factor receptor)	1.89
GPR92	57121	G protein-coupled receptor 92	-1.86
TNFRSF14	8764	tumor necrosis factor receptor superfamily, member 14 (herpesvirus entry mediator)	-1.84
C1S	716	complement component 1, s subcomponent	2.36
WNT5A	7474	wingless-type MMTV integration site family, member 5A	1.67
NETO2	81831	neuropilin (NRP) and tolloid (TLL)-like 2	2.04
HMOX1	3162	heme oxygenase (decycling) 1	1.52
ITGB6	3694	integrin, beta 6	1.54
RAF1	5894	v-raf-1 murine leukemia viral oncogene homolog 1	1.72
PVR	5817	poliovirus receptor	1.57
LGR4	55366	leucine-rich repeat-containing G protein-coupled receptor 4	-2.06
LMBR1L	55716	limb region 1 homolog (mouse)-like	1.76
GRINA	2907	glutamate receptor, ionotropic, N-methyl D-asparate-associated protein 1 (glutamate binding)	2.03

F2RL1	2150	coagulation factor II (thrombin) receptor-like 1	-1.53
Genes in transcription regulator activity			
NRG1	3084	neuregulin 1	2.69
RAN	5901	RAN, member RAS oncogene family	2.02
TEAD2	8463	TEA domain family member 2	1.99
CREG1	8804	cellular repressor of E1A-stimulated genes 1	1.97
IFI16	3428	interferon, gamma-inducible protein 16	1.95
ZFP36L1	677	zinc finger protein 36, C3H type-like 1	1.92
CBX2	84733	chromobox homolog 2 (Pc class homolog, Drosophila)	1.86
E2F2	1870	E2F transcription factor 2	1.85
GABPB2	2553	GA binding protein transcription factor, beta subunit 2	1.8
TP73L	8626	tumor protein p73-like	1.72
E2F3	1871	E2F transcription factor 3	1.6
FOXJ3	22887	forkhead box J3	1.6
GTF3A	2971	general transcription factor IIIA	1.59
CEBPG	1054	CCAAT/enhancer binding protein (C/EBP), gamma	1.57
SMARCA4	6597	SWI/SNF related, matrix associated, actin dependent regulator of chromatin, subfamily a, member 4	1.56
E2F6	1876	E2F transcription factor 6	1.56
ZNF219	51222	zinc finger protein 219	1.55
CREB3L2	64764	cAMP responsive element binding protein 3-like 2	1.53
NFE2L3	9603	nuclear factor (erythroid-derived 2)-like 3	1.52
ILF3	3609	interleukin enhancer binding factor 3, 90kDa	1.52
NFIL3	4783	nuclear factor, interleukin 3 regulated	1.51
SUPT4H1	6827	suppressor of Ty 4 homolog 1 (S. cerevisiae)	1.5
ETS1	2113	v-ets erythroblastosis virus E26 oncogene homolog 1 (avian)	1.5
CITED2	10370	Cbp/p300-interacting transactivator, with Glu/Asp-rich carboxy-terminal domain, 2	-1.51
NMI	9111	N-myc (and STAT) interactor	-1.6
ELF3	1999	E74-like factor 3 (ets domain transcription factor, epithelial-specific)	-1.61
HOXB5	3215	homeobox B5	-1.65
TSC22D3	1831	TSC22 domain family, member 3	-1.86
Genes in translation regulator activity			
EEF1A2	1917	eukaryotic translation elongation factor 1 alpha 2	2.29
PET112L	5188	PET112-like (yeast)	-1.55
EIF4A2	1974	eukaryotic translation initiation factor 4A, isoform 2	-1.83
Genes in transporter activity			
TNFAIP1	7126	tumor necrosis factor, alpha-induced protein 1 (endothelial)	1.5
SLC22A18	5002	solute carrier family 22 (organic cation transporter), member 18	-1.51
PLSCR1	5359	phospholipid scramblase 1	-1.55
SLC16A5	9121	solute carrier family 16, member 5 (monocarboxylic acid transporter	-1.92
AP2M1	1173	adaptor-related protein complex 2, mu 1 subunit	1.52
SCNN1A	6337	sodium channel, nonvoltage-gated 1 alpha	-1.64
SLC25A22	79751	solute carrier family 25 (mitochondrial carrier: glutamate), member 22	3.18
TMCO3	55002	transmembrane and coiled-coil domains 3	1.72
KIAA0528	9847	KIAA0528	-1.52
PHGDH	26227	phosphoglycerate dehydrogenase	2.1
SCARB1	949	scavenger receptor class B, member 1	2.34
IGF2R	3482	insulin-like growth factor 2 receptor	1.65
ABCA1	19	ATP-binding cassette, sub-family A (ABC1), member 1	1.64
SEC61A1	29927	Sec61 alpha 1 subunit (S. cerevisiae)	1.71

GJA1	2697	gap junction protein, alpha 1, 43kDa (connexin 43)	1.64
M6PR	4074	mannose-6-phosphate receptor (cation dependent)	1.54
FXYD3	5349	FXYD domain containing ion transport regulator 3	-1.94
SLC38A1	81539	solute carrier family 38, member 1	1.53
ATP5H	10476	ATP synthase, H+ transporting, mitochondrial F0 complex, subunit d	-1.5
ATP1B3	483	ATPase, Na+/K+ transporting, beta 3 polypeptide	1.59
SLC35B1	10237	solute carrier family 35, member B1	2.14
ATG9A	79065	ATG9 autophagy related 9 homolog A (S. cerevisiae)	1.51
SLC37A4	2542	solute carrier family 37 (glycerol-6-phosphate transporter), member 4	-1.68
KCNS1	3787	potassium voltage-gated channel, delayed-rectifier, subfamily S,	-1.81
SLC39A6	25800	solute carrier family 39 (zinc transporter), member 6	1.99
