# Supplementary material for: Claudin-7 Is Frequently Overexpressed in Ovarian Cancer and Promotes Invasion
Source: PLoS One. 2011 Jul 15;6(7):e22119. doi: 10.1371/journal.pone.0022119 (PMC3137611; doi:10.1371/journal.pone.0022119)
Supplement: Table S5 — KEGG pathways enriched following CLDN7 knockdown. (PDF) [file pone.0022119.s005.pdf]

Supp Table 5: Webgestalt analysis

OVCAR2

KEGG pathway	Gene number	Enrichment
Regulation of actin cytoskeleton	16	O=16;E=3.9463;R=4.0544;P=5.54e-6
Cytokine-cytokine receptor interaction	16	O=16;E=4.8478;R=3.3005;P=5.87e-5
Focal adhesion	14	O=14;E=3.8061;R=3.6783;P=5.70e-5
Oxidative phosphorylation	13	O=13;E=2.2636;R=5.7431;P=1.29e-6
Tight junction	12	O=12;E=2.2436;R=5.3485;P=6.42e-6
MAPK signaling pathway	12	O=12;E=5.3285;R=2.252;P=9.39e-3
Insulin signaling pathway	11	O=11;E=2.6042;R=4.2239;P=1.13e-4
Ribosome	10	O=10;E=1.9231;R=5.1999;P=4.68e-5
Jak-STAT signaling pathway	9	O=9;E=2.8646;R=3.1418;P=3.22e-3
Wnt signaling pathway	9	O=9;E=2.8646;R=3.1418;P=3.22e-3

OVCA420

KEGG pathway	Gene number	Enrichment
Regulation of actin cytoskeleton	15	O=15;E=2.4977;R=6.0055;P=8.74e-8
Focal adhesion	14	O=14;E=2.409;R=5.8115;P=3.42e-7
Insulin signaling pathway	12	O=12;E=1.6483;R=7.2802;P=2.55e-7
MAPK signaling pathway	9	O=9;E=3.3726;R=2.6686;P=8.22e-3
Leukocyte transendothelial migration	9	O=9;E=1.42;R=6.338;P=2.28e-5
Natural killer cell mediated cytotoxicity	9	O=9;E=1.5088;R=5.965;P=3.56e-5
Gap junction	9	O=9;E=1.0777;R=8.3511;P=2.89e-6
Wnt signaling pathway	8	O=8;E=1.8131;R=4.4123;P=6.62e-4
Pancreatic cancer	8	O=8;E=0.9002;R=8.8869;P=6.78e-6
Cell adhesion molecules (CAMs)	7	O=7;E=1.5722;R=4.4524;P=1.35e-3
