# Supplementary material for: Claudin-7 Is Frequently Overexpressed in Ovarian Cancer and Promotes Invasion
Source: PLoS One. 2011 Jul 15;6(7):e22119. doi: 10.1371/journal.pone.0022119 (PMC3137611; doi:10.1371/journal.pone.0022119)
Supplement: Table S6 — Experimentally-validated interactions among the differentially regulated genes using Pathway Studio. (PDF) [file pone.0022119.s006.pdf]

Supp. Table 6: Experimentally proven interactions among differentially regulated genes in our study

Interaction Type	Relation	MedLine Reference	Connectivity	# of References
Expression	RAF1 ---> HRAS	8888693:1124	2	51
Expression	SREBF1 --+> PCK2	12867535:1191	2	10
Regulation	HRAS ---> MAPK3	12839994:1194	2	37
Regulation	VAV2 --+> Rac1	10982832:12	2	38
MolTransport	TNF --+> Rac1	15242860:12	2	4
MolTransport	MAP2K1 ---| TNF	10026206:4	2	8
MolTransport	PRKCZ --+> MAPK3	10614633:7	2	6
ProtModification	RAF1 --+> MAP2K1	9677429:3	2	661
ProtModification	HRAS ---> RAF1	9611107:1238	2	1
ProtModification	MAP2K1 --+> MAPK3	11496823:2	2	593
ProtModification	RAF1 --+> MAPK3	9395450:1214	2	154
ProtModification	MAPK3 ---> MAP2K1	9361191:3	2	64
ProtModification	MAPK3 --+> RAF1	9804618:4	2	28
ProtModification	RPS6KB1 ---> MAPK3	12832467:1179	2	1
ProtModification	MAPK3 <++> RPS6KB1	8001266:1248	2	1
ProtModification	RAF1 ---| MAPK3	10952668:10	2	64
ProtModification	RAF1 ---> MAP2K1	11018021:2	2	90
ProtModification	MAPK3 --+> SREBF1	9712704:3	2	6
ProtModification	MAP2K1 ---> MAPK3	9353338:1021	2	102
ProtModification	MAPK3 ---> RAF1	10807911:5	2	14
ProtModification	FGFR3 --+> MAPK3	9045692:1266	2	1
ProtModification	RPS6KB1 ---> RPS6	8663120:0	2	105
ProtModification	CSK ---> CAV1	11805080:1088	2	16
ProtModification	PRKCZ --+> RAF1	10891480:3	2	5
ProtModification	RPS6KB1 --+> MAPK3	15696050:8	2	12
ProtModification	MAPK3 ---| RPS6KB1	11241670:6	2	8
ProtModification	FGFR3 ---> MAPK3	9045692:1266	2	2
ProtModification	MAPK3 ---> FOSL1	12414630:5	2	6
ProtModification	MAPK3 ---> SREBF1	9712704:3	2	3
ProtModification	DUSP3 ---> MAPK3	7836374:1027	2	3
ProtModification	DUSP3 ---| MAPK3	7836374:1027	2	3
Binding	HRAS ---- RAF1	15664191	2	59
Binding	ITGAV ---- ITGB6	8798654	2	14
Binding	CAV1 ---- Rac1	15263006:7	2	3
Binding	Rac1 ---- RPS6KB1	11713255:1053	2	1
Binding	Actb ---- Rac1	11027608:2	2	1
Binding	HSPA1A ---- MAP2K1	12755708:1263	2	1
Binding	CAV1 ---- ITGAV	11683383:1142	2	1
Binding	GIT1 ---- MAP2K1	15923189:1	2	2
Binding	CSK ---- GRF2	12865333:1238	2	1
Binding	GRF2 ---- HRAS	10514505:1220	2	1
Binding	GIT1 ---- Rac1	12686588:1032	2	1
Binding	MAPK3 ---- PPP1CA	12624094:1152	2	1
Binding	CAV1 ---- PPP1CA	14645548:1219	2	7
Binding	PPP1CA ---- RPS6KB1	12036952:1149	2	1
Binding	RAF1 ---- RRAS	8999998:1	2	13
Binding	PPP1CA ---- RAF1	12374792:1020	2	1

Binding	HSPA8 ---- RAF1	12721288:1270	2	1
Binding	HSPA1A ---- HSPA8	12944486:1	2	21
Binding	PPP1CA ---- PYGB	9013623:1221	2	1
Binding	ARHQ ---- GRF2	15581361:0	2	1
Binding	BAIAP2 ---- Rac1	14506233:1030	2	2
Binding	CAV1 ---- MAPK3	12969891:1245	2	1
Binding	HSPA1A ---- RAF1	15625278:3	2	3
Binding	CAV1 ---- CSK	11805080:1088	2	3
Binding	PRKCZ ---- RAF1	12551925:1268	2	1
Binding	ARHQ ---- PRKCZ	3952493131	2	2
Binding	HRAS ---- MAP2K1	8822208:1	2	1
Binding	PRKCZ ---- RPS6KB1	10082559:10	2	1
Binding	MAPK3 ---- RPS6KB1	15501927:9	2	1
Binding	PPP2R2C ---- RAF1	4152281798	2	6
PromoterBinding	CAV1 ---- SREBF1	15531587:1	2	7
DirectRegulation	HRAS ---> RAF1	12077341:4	2	47
DirectRegulation	RRAS ---> RAF1	8620503:3	2	9
DirectRegulation	HRAS --+> MAP2K1	9397154:4	2	2
DirectRegulation	HRAS ---| CAV1	8621645:1247	2	9
DirectRegulation	CAV1 ---| HRAS	9488658:1040	2	14
DirectRegulation	CAV1 --+> CAV2	14713860:5	2	17
DirectRegulation	PRKCZ --+> RPS6KB1	11980660:8	2	3
DirectRegulation	HSPA1A ---| RAF1	15625278:3	2	6
DirectRegulation	RAF1 --+> HSPA1A	15831476:1	2	4
DirectRegulation	MAP2K1 ---> HRAS	8670897:9	2	2
DirectRegulation	CSK ---- MAP2K1	11964302:1140	2	2
DirectRegulation	MAP2K1 --+> RAF1	9690518:4	2	46
DirectRegulation	CAV1 ---> CSK	15471980:5	2	5
DirectRegulation	HRAS --+> RAF1	9115221:1030	2	88
DirectRegulation	RAF1 ---| CAV1	11953426:1394	2	2
DirectRegulation	CAV1 ---| MAPK3	11948420:10	2	7
DirectRegulation	CSK ---| HRAS	12695509:1245	2	1
DirectRegulation	MAPK3 ---> CAV1	12388423:6	2	2
DirectRegulation	HSPA5 ---> HSPA1A	8200354:2	2	4
DirectRegulation	RRAS --+> RAF1	8999998:1	2	24
DirectRegulation	RRAS --+> HRAS	9852038:1195	2	7
DirectRegulation	HSPA1A --+> HSPA8	10671488:1132	2	3
DirectRegulation	RRAS2 --+> RAF1	10557073:6	2	10
DirectRegulation	GRF2 --+> ARHQ	11309621:6	2	9
DirectRegulation	PPP1CA ---> RAF1	12374792:8	2	1
DirectRegulation	HSPA8 --+> HSPA1A	9148760:2	2	26
DirectRegulation	GIT1 ---> MAP2K1	15923189:1	2	3
DirectRegulation	RAF1 ---| HRAS	10777480:1229	2	42
DirectRegulation	CAV2 --+> CAV1	10373486:1183	2	17
